# Disease‐linked TDP‐43 hyperphosphorylation suppresses TDP‐43 condensation and aggregation

**DOI:** 10.15252/embj.2021108443

**Published:** 2022-02-03

**Authors:** Lara A Gruijs da Silva, Francesca Simonetti, Saskia Hutten, Henrick Riemenschneider, Erin L Sternburg, Lisa M Pietrek, Jakob Gebel, Volker Dötsch, Dieter Edbauer, Gerhard Hummer, Lukas S Stelzl, Dorothee Dormann

**Affiliations:** ^1^ Biocenter Institute of Molecular Physiology Johannes Gutenberg‐Universität (JGU) Mainz Germany; ^2^ Graduate School of Systemic Neurosciences (GSN) Planegg‐Martinsried Germany; ^3^ German Center for Neurodegenerative Diseases (DZNE) Munich Germany; ^4^ Department of Theoretical Biophysics Max Planck Institute of Biophysics Frankfurt am Main Germany; ^5^ Institute for Biophysical Chemistry Goethe‐Universität Frankfurt am Main Germany; ^6^ Munich Cluster for Systems Neurology (SyNergy) Munich Munich Germany; ^7^ Institute for Biophysics Goethe‐Universität Frankfurt am Main Germany; ^8^ KOMET1 Institute of Physics Johannes Gutenberg‐Universität (JGU) Mainz Germany; ^9^ Institute of Molecular Biology (IMB) Mainz Germany

**Keywords:** neurodegeneration, phase separation, phosphorylation, RNA‐binding protein, TDP‐43, Neuroscience, RNA Biology

## Abstract

Post‐translational modifications (PTMs) have emerged as key modulators of protein phase separation and have been linked to protein aggregation in neurodegenerative disorders. The major aggregating protein in amyotrophic lateral sclerosis and frontotemporal dementia, the RNA‐binding protein TAR DNA‐binding protein (TDP‐43), is hyperphosphorylated in disease on several C‐terminal serine residues, a process generally believed to promote TDP‐43 aggregation. Here, we however find that Casein kinase 1δ‐mediated TDP‐43 hyperphosphorylation or C‐terminal phosphomimetic mutations reduce TDP‐43 phase separation and aggregation, and instead render TDP‐43 condensates more liquid‐like and dynamic. Multi‐scale molecular dynamics simulations reveal reduced homotypic interactions of TDP‐43 low‐complexity domains through enhanced solvation of phosphomimetic residues. Cellular experiments show that phosphomimetic substitutions do not affect nuclear import or RNA regulatory functions of TDP‐43, but suppress accumulation of TDP‐43 in membrane‐less organelles and promote its solubility in neurons. We speculate that TDP‐43 hyperphosphorylation may be a protective cellular response to counteract TDP‐43 aggregation.

## Graphical abstract

C‐terminal TDP‐43 hyperphosphorylation may be a protective cellular response to counteract TDP‐43 aggregation, rather than a driver of neuropathology as previously assumed.

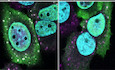

## Introduction

TAR DNA‐binding protein (TDP‐43) is the major aggregating protein in amyotrophic lateral sclerosis (ALS) and frontotemporal dementia (FTD) patients and also forms pathological aggregates in up to 50% of Alzheimer's disease patients (Neumann *et al*, 2006; Josephs *et al*, 2014). It is a ubiquitously expressed RNA‐binding protein (RBP) with key functions in RNA processing, e.g., regulation of alternative splicing and polyadenylation, miRNA processing, mRNA stability and localization (Ratti & Buratti, 2016). In the affected brain regions of ALS and FTD patients, the physiological diffuse nuclear localization of TDP‐43 is lost. Instead the protein forms cytoplasmic and occasionally nuclear inclusions in neurons and glial cells (Mackenzie *et al*, 2010). TDP‐43 pathology closely correlates with neurodegeneration, and both loss‐of‐function mechanisms, e.g., misregulation of nuclear RNA targets, and gain‐of‐function mechanisms, e.g., aberrant interactions of the TDP‐43 aggregates, are believed to contribute to neuronal dysfunction and eventually neurodegeneration (Ling *et al*, 2013; Tziortzouda *et al*, 2021).

Similar to other prion‐like RBPs, TDP‐43 is thought to aggregate through aberrant liquid–liquid phase separation (LLPS), i.e., the transition of liquid‐like RBP condensates into a solid‐like state (Nedelsky & Taylor, 2019). Aberrant phase transitions may occur in stress granules (SGs) or other membrane‐less organelles (MLOs), where aggregation‐prone RBPs are highly concentrated and exceed the critical concentration for LLPS (Alberti & Dormann, 2019; Alberti & Hyman, 2021). Subsequent liquid‐to‐solid phase transition, as demonstrated for various disease‐linked RBPs *in vitro* (Molliex *et al*, 2015; Patel *et al*, 2015), may then cause formation of pathological RBP inclusions. LLPS is often driven by intrinsically disordered low complexity domains (LCDs), that tend to engage in weak multivalent interactions with other molecules (Alberti, 2017). TDP‐43 harbors a long C‐terminal LCD enriched in glycine, serine, asparagine and glutamine residues, which drives intermolecular TDP‐43 interactions and assembly by phase separation (Conicella *et al*, 2016; Babinchak *et al*, 2019). The LCD is also the region that harbors numerous ALS‐linked point mutations (Buratti, 2015), suggesting that small chemical changes to the TDP‐43 LCD can cause neurodegeneration.

Liquid–liquid phase separation and MLO dynamics are often regulated by post‐translational modifications (PTMs) in LCDs, as the introduction of small chemical groups or proteins changes the chemical nature of amino acids, e.g., their charge or hydrophobicity, which can alter their molecular interactions and LLPS behavior (Bah & Forman‐Kay, 2016; Hofweber & Dormann, 2019). A highly disease‐specific PTM on deposited TDP‐43 inclusions is hyperphosphorylation on C‐terminal serine residues in the LCD (Hasegawa *et al*, 2008; Inukai *et al*, 2008; Neumann *et al*, 2009; Kametani *et al*, 2016). Antibodies specific for C‐terminal TDP‐43 phosphorylation sites (e.g., S409/S410 and S403/S404) detect inclusion pathology in patients, without cross‐reactivity with physiological nuclear TDP‐43. Therefore, C‐terminal TDP‐43 hyperphosphorylation is considered a pathological hallmark and is generally believed to promote TDP‐43 aggregation (Buratti, 2018). This view is largely based on the observations that C‐terminal TDP‐43 phosphorylation correlates with inclusion pathology and that overexpression of kinases that can phosphorylate TDP‐43 enhance TDP‐43 aggregation and neurotoxicity (Choksi *et al*, 2014; Liachko *et al*, 2014; Nonaka *et al*, 2016; Taylor *et al*, 2018). Based on these studies, inhibition of TDP‐43 phosphorylation by specific kinase inhibitors has even been proposed as a potential therapeutic strategy for ALS (Liachko *et al*, 2013; Salado *et al*, 2014; Martinez‐Gonzalez *et al*, 2020). However, the molecular consequences of this disease‐linked PTM are still poorly understood, and its effects on TDP‐43 LLPS and aggregation are still unknown.

Using *in vitro*, *in silico* and cellular experiments, we now demonstrate that disease‐linked C‐terminal hyperphosphorylation of TDP‐43 suppresses TDP‐43 condensation and insolubility. We show this through (i) *in vitro* phase separation and aggregation assays with recombinant, full‐length TDP‐43; (ii) coarse‐grained and atomistic molecular dynamics (MD) simulations of condensates of TDP‐43 LCDs, elucidating molecular driving forces; and (iii) experiments in HeLa cells, stable inducible U2OS cells and primary rat neurons, where C‐terminal phosphomimetic mutations do not disturb nuclear import or RNA processing functions of TDP‐43, but abrogate TDP‐43 condensation into MLOs and enhance its solubility. Based on our findings, we speculate that C‐terminal TDP‐43 hyperphosphorylation may be a protective cellular response to counteract TDP‐43 solidification, rather than being a driver of TDP‐43 pathology, as has so far been assumed.

## Results

### *In vitro* phosphorylation with Casein kinase 1δ reduces condensation of TDP‐43

**Figure EV1:**
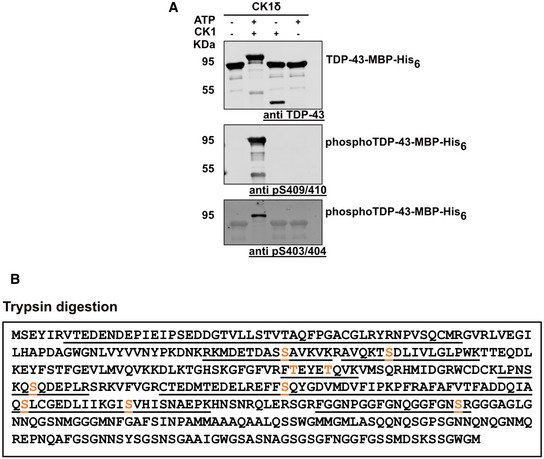
Identification of TDP‐43‐MBP‐His_6_ phospho‐sites after *in vitro* phosphorylation with CK1δ Identification of TDP‐43 phospho‐sites on *in vitro* phosphorylated TDP‐43 (+CK1δ, +ATP) in comparison to controls (−CK1δ −ATP; CK1δ only; ATP only) by Western blot. Samples were analyzed by SDS–PAGE and Western blot using a rabbit anti‐TDP‐43 N‐term antibody (Proteintech) to detect total TDP‐43, rat anti‐TDP‐43‐phospho Ser409/410 (clone 1D3, Helmholtz Center Munich) and mouse anti‐TDP‐43‐phospho Ser403/404 (Proteintech, Cat. No.: 66079‐1‐Ig) antibodies.Schematic diagrams showing sequence coverage in mass spectrometry after trypsin digest (underlined) and phosphorylated serine/threonine residues (orange) of *in vitro* phosphorylated TDP‐43‐MBP‐His_6_ with CK1δ + ATP (one out of two representative experiments is shown). Source data are available online for this figure.

**Figure 1:**
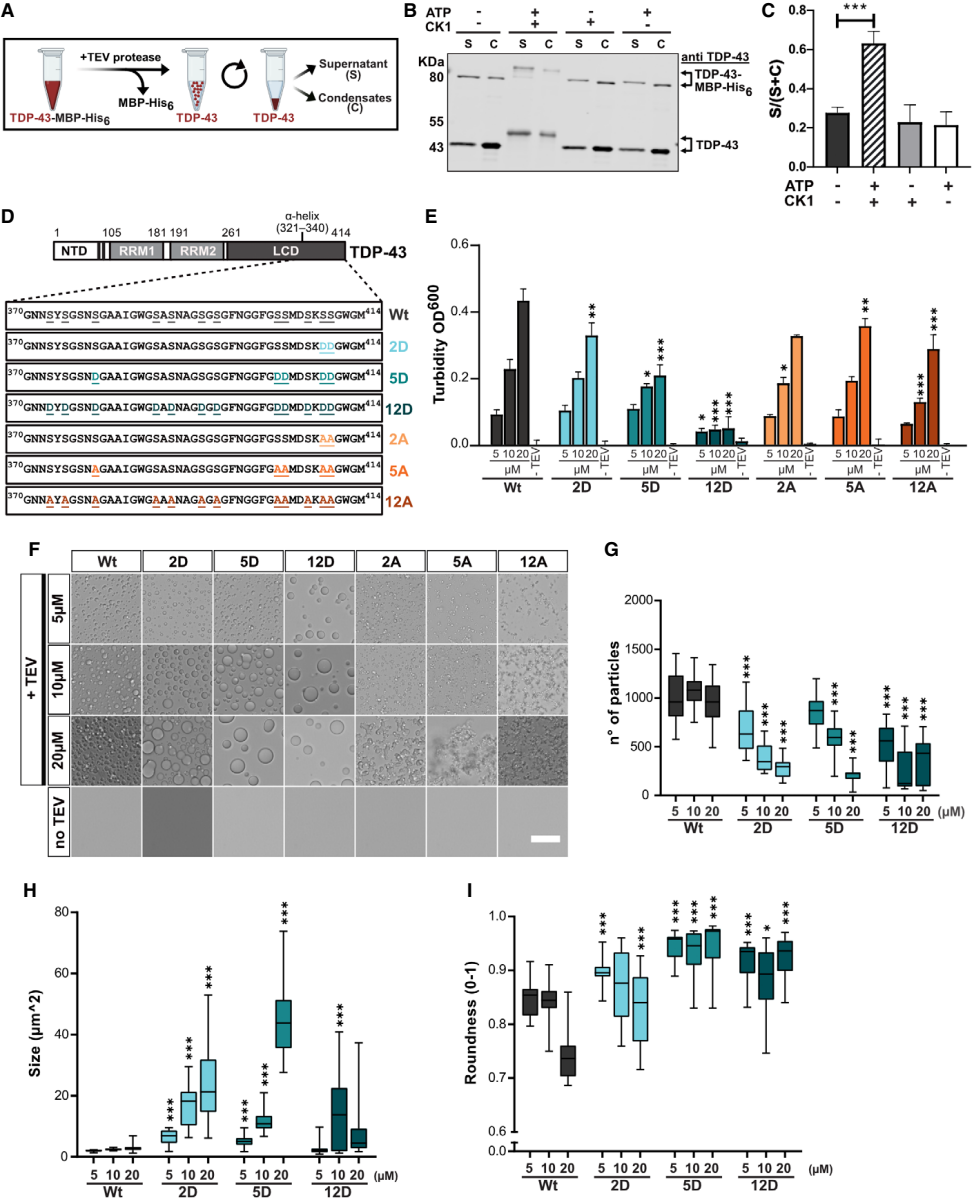
TDP‐43 phosphorylation by CK1δ and C‐terminal phosphomimetic substitutions reduce TDP‐43 condensation *in vitro* AScheme of sedimentation assay (created in BioRender.com): phase separation of TDP‐43 was induced by TEV protease cleavage of TDP‐43‐MBP‐His_6_, and condensates were pelleted by centrifugation.BSedimentation assay to quantify condensation of unmodified TDP‐43 versus *in vitro* phosphorylated TDP‐43 (+CK1δ, +ATP) and controls (CK1δ or ATP only); TDP‐43 detected by Western blot (rabbit anti‐TDP‐43 N‐term). Due to incomplete TEV cleavage, some TDP‐43‐MBP‐His_6_ remains present and co‐fractionates with cleaved TDP‐43, due to TDP‐43 self–self interaction.CQuantification of band intensities of cleaved TDP‐43 shown as mean of Supernatant/(Supernatant + Condensate) (S/[S + C]) ratio of three independent experimental replicates (*n* = 3) ± SD. ***P < 0.0002 by one‐way ANOVA with Dunnett's multiple comparison test to Wt.DSchematic diagram of TDP‐43 and sequence of C‐terminal region (aa. 370–414) for Wt, phosphomimetic (S‐to‐D) variants and control (S‐to‐A) variants. NTD, N‐terminal domain; RRM, RNA recognition motif; LCD, low complexity domain with α‐helical structure.ETurbidity measurements (optical density [OD] at 600 nm) to quantify phase separation of the indicated TDP‐43 variants at three different concentrations (in Hepes buffer). Values represent mean of three independent experimental replicates (*n* = 3) ± SD. *P < 0.0332, **P < 0.0021 and ***P < 0.0002 by one‐way ANOVA with Dunnett's multiple comparison test to Wt, comparing the respective concentration condition (5, 10 and 20 µM).F–IRepresentative bright field microscopic images of TDP‐43 condensates (in Hepes buffer), Bar, 25 µm (F) and quantification of condensate number (G), size (H) and roundness (I). Box plots show the comparison of median and inter‐quartile range (upper and lower quartiles) of all fields of view (FOV) from Min to Max (whiskers) of two replicates (≥ 22 FOV per condition). *P < 0.0332, **P < 0.0021 and ***P < 0.0002 by one‐way ANOVA with Dunnett's multiple comparison test to Wt, comparing the respective concentration condition (5, 10 and 20 µM). Source data are available online for this figure.

To examine how phosphorylation affects TDP‐43 phase transitions, we expressed and purified unphosphorylated full‐length TDP‐43 with a solubilizing MBP tag and a His_6_‐tag in *Escherichia coli* (Wang *et al*, 2018) (Appendix Fig S1A–E). We then *in vitro* phosphorylated the purified protein with casein kinase 1 delta (CK1δ), a kinase previously reported to phosphorylate TDP‐43 at disease‐associated sites (Kametani *et al*, 2009), and confirmed phosphorylation of C‐terminal serines (S403/S404; S409/S410) with phospho‐specific antibodies (Fig EV1A). Mass spectrometric analysis detected phosphorylation on several additional serine/threonine sites (Fig EV1B), and the running behavior in SDS–PAGE suggests hyperphosphorylation on multiple sites (Figs 1B and EV1A). We then induced phase separation of the unphosphorylated vs *in vitro* phosphorylated TDP‐43 by cleaving off the MBP tag with TEV protease (Wang *et al*, 2018) and used centrifugation to separate the condensates (C) from the cleared supernatant (S; Fig 1A). Cleaved TDP‐43 was mostly in the condensate fraction (S/[S + C] ratio ~0.25), whereas *in vitro* phosphorylated TDP‐43 was predominantly in the supernatant (S/[S + C] ratio > 0.6; Fig 1B and C). Reduced sedimentation of TDP‐43 was not seen upon addition of adenosine triphosphate (ATP) or CK1δ alone, suggesting that it is indeed caused by the addition of phospho‐groups to TDP‐43.

### C‐terminal phosphomimetic substitutions mimicking disease‐linked phosphorylation suppress TDP‐43 phase separation

To study defined disease‐linked phosphorylation sites, we generated phosphomimetic proteins harboring different numbers of phosphomimetic serine‐to‐aspartate (S‐to‐D) mutations or corresponding serine‐to‐alanine (S‐to‐A) mutations as control. Phosphomimetic substitutions rely on the replacement of a phosphorylated serine or threonine with a negatively charged amino acid (D or E), thus mimicking the negative charge of the phospho group. Even though they under‐appreciate the charge change (net charge of aspartate = −1 instead of −2 for a phospho‐group) and do not always accurately mimic the chemistry of a phospho group, phosphomimetics have been successfully used to probe the biological function of phosphorylated residues (Martin *et al*, 2014). Phosphorylation on S409/S410 is a highly specific and consistent feature of aggregated TDP‐43 in all ALS/FTD subtypes (Inukai *et al*, 2008; Neumann *et al*, 2009), and five phosphorylation sites (S379, S403, S404, S409 and S410) were detected with phosphorylation site‐specific antibodies in human post‐mortem tissue (Hasegawa *et al*, 2008). Therefore, we mutated these serines to create "2D" and "5D" variants as well as the corresponding “2A” and “5A” controls (Fig 1D). Based on a mass spectrometric study that found phosphorylation on 12 out of 14 serines in the C‐terminal LCD of TDP‐43 in ALS spinal cord (Kametani *et al*, 2016), we also mutated these 12 sites (S373, S375, S379, S387, S389, S393, S395, S403, S404, S407, S409 and S410) to create “12D” or “12A” variants (Fig 1D). Interestingly, the PLAAC web tool (http://plaac.wi.mit.edu/) that allows prediction of probable prion subsequences using a hidden‐Markov model (HMM) algorithm (Lancaster *et al*, 2014), predicted a reduced prion‐like character of the C‐terminal region in the phosphomimetic 12D variant compared with the wild‐type (Wt) and 12A protein (Appendix Fig S2).

**Figure EV2:**
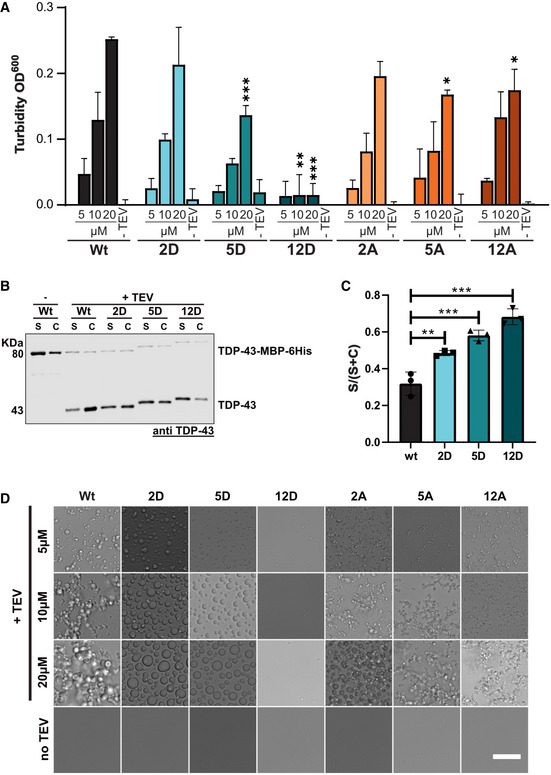
C‐terminal phosphomimetic substitutions reduce TDP‐43 condensation *in vitro* Turbidity measurements (optical density [OD] at 600 nm) to quantify phase separation of different S‐to‐D and S‐to‐A mutants in comparison to TDP‐43 Wt using phosphate buffer. Values represent mean of three independent experimental replicates (*n* = 3) ± SD. *P < 0.0332, **P < 0.0021 and ***P < 0.0002 by one‐way ANOVA with Dunnett's multiple comparison test to Wt, comparing the respective concentration condition (5, 10, 20 µM).Sedimentation assay to quantify condensation of different S‐to‐D mutants in comparison to TDP‐43 Wt (in Hepes buffer). TDP‐43 was detected by TDP‐43 Western blot (rabbit anti‐TDP‐43 N‐term).Quantification of band intensities of cleaved TDP‐43 corresponding to supernatant (S) and condensates (C) fractions is shown as mean of S/(S + C) ratio of three independent experimental replicates (*n* = 3) ± SD. **P < 0.0021 and ***P < 0.0002 by one‐way ANOVA with Dunnett's multiple comparison test to Wt.Representative bright field microscopic images of TDP‐43 condensates formed from TDP‐43 Wt vs different S‐to‐D or S‐to‐A variants in phosphate buffer (Bar, 25 µm). Source data are available online for this figure.

To study phase separation experimentally, all variants were expressed and purified as TDP‐43‐MBP‐His_6_ fusion proteins (Appendix Fig S1A–E), and phase separation induced by TEV protease‐mediated cleavage of the MBP tag was examined by turbidity, sedimentation or microscopic condensate assays. Turbidity measurements revealed a concentration‐dependent increase in phase separation for TDP‐43 Wt, as expected, whereas the increase was less pronounced for the 2D and 5D variants and no concentration‐dependent increase was seen for the 12D mutant (Fig 1E). The gradual decrease in turbidity caused by the phosphomimetic mutations (Wt > 2D > 5D > 12D) was not seen to the same extent for the corresponding S‐to‐A control mutations (Fig 1E), hence suppression of phase separation is not due to the loss of serines at these positions, but can be attributed to the additional negative charges introduced by the D substitutions. Turbidity assays in phosphate buffer instead of Hepes buffer gave similar results (Fig EV2A), and sedimentation assays confirmed that TDP‐43 condensation is gradually suppressed by increasing numbers of phosphomimetic mutations (Fig EV2B and C).

### Phosphomimetic S‐to‐D substitutions lead to rounder TDP‐43 condensates, whereas S‐to‐A mutations cause an amorphous, aggregate‐like morphology

Interestingly, bright field microscopy revealed that TDP‐43 Wt formed relatively small, amorphous condensates, suggestive of solid‐like material properties (Fig 1F). In contrast, the phosphomimetic S‐to‐D proteins formed fewer, but much larger and rounder condensates (Fig 1F, see G–I for quantification), suggesting a more liquid‐like behavior and therefore fusion of condensates into larger droplets. Again, the observed changes were correlated with the number of phosphomimetic mutations, i.e., they were most pronounced for the 12D mutant, which formed very few, but large and perfectly circular protein droplets. (Note that these few large condensates most likely escape detection in the turbidity assay due to rapid sedimentation during the assay.) In contrast, the S‐to‐A control variants formed numerous small, amorphous condensates and had a more irregular, aggregate‐like appearance than TDP‐43 Wt (Fig 1F). This phenotype suggests that the OH groups in the respective serines influence the material properties of TDP‐43 and contribute to preventing its aggregation. Similar results were obtained when the assay was carried out in phosphate buffer instead of Hepes buffer, except that 12D formed only very few, small condensates in phosphate buffer (Fig EV2D), possibly because the ions in phosphate buffer may screen certain attractive interactions between TDP‐43 molecules and disfavor phase separation. Together, these results demonstrate that phosphomimetic substitutions mimicking disease‐linked C‐terminal TDP‐43 phosphorylation reduce the tendency of TDP‐43 to phase separate into amorphous condensates and suggest a more dynamic, liquid‐like behavior of C‐terminally phosphorylated TDP‐43.

### C‐terminal phosphomimetic substitutions yield more liquid‐like, dynamic TDP‐43 condensates

**Figure 2:**
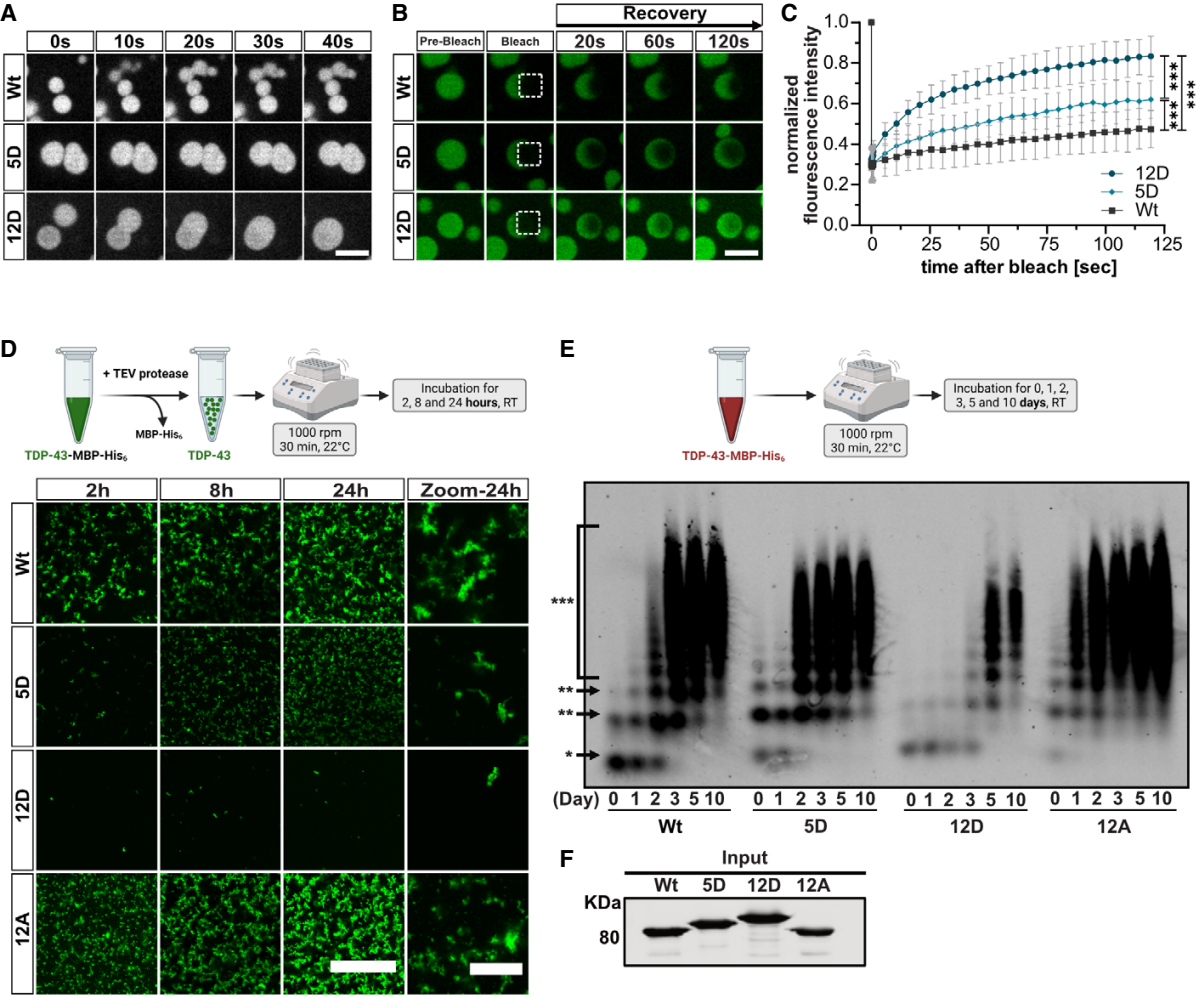
C‐terminal phosphomimetic substitutions enhance liquidity of TDP‐43 condensates and reduce TDP‐43 aggregation *in vitro* Representative still images of Alexa488‐labeled TDP‐43 condensates by spinning disc timelapse confocal microscopy. Wt condensates do not fuse, 5D condensates fuse slowly and 12D condensates readily fuse upon contact and relax into spherical droplets. Bar, 5 µm.Representative images of FRAP experiments at indicated time‐points. Boxes indicate bleached area (half‐bleach of condensate). Bar, 5 µm.FRAP curves after half‐bleach of freshly formed Alexa488‐labeled TDP‐43 condensates. Values represent mean ± SD of three independent experimental replicates (*n* = 3) of ≥ 9 droplets analyzed per condition. ***P < 0.0002 by one‐way ANOVA with Tukey's multiple comparison test for area under the curve (AUC) of individual droplets.Confocal images of Alexa488‐labeled TDP‐43 aggregates formed in an *in vitro* aggregation assay (with TEV protease cleavage). Bar, 100 µm. Zoom shows magnified view of aggregates at the 24 h time point. Bar, 20 µm.SDD‐AGE followed by TDP‐43 Western blot to visualize SDS‐resistant oligomers/high‐molecular‐weight species of TDP‐43‐MBP‐His_6_ in an *in vitro* aggregation assay (without TEV protease cleavage). Asterisks represent monomeric (*), oligomeric (**) and polymeric (***) species.Input of TDP‐43‐MBP‐His_6_ variants used in the SDD‐AGE assay, detected by Western blot (anti‐TDP‐43 N‐term). Source data are available online for this figure.

To test whether the phosphomimetic mutations indeed render TDP‐43 more liquid‐like, we performed live imaging of Alexa488‐labeled Wt, 5D and 12D condensates by spinning disc confocal microcopy. For TDP‐43 Wt, no fusion events were observed over a time course of several minutes. Instead the small condensates stuck to each other in a chain‐like arrangement (Movie EV1, Fig 2A). In contrast, 5D condensates occasionally and slowly fused with each other, and 12D condensates readily fused upon contact and relaxed into perfectly round spheres, indicating a liquid droplet‐like nature (Movies EV2 and EV3, Fig 2A). To assess the mobility of TDP‐43 molecules in condensates, we performed half‐bleaches of condensates and analyzed fluorescent recovery after photobleaching (FRAP) in the bleached half. In TDP‐43 Wt condensates, fluorescence recovered very slowly, indicating a low mobility of TDP‐43 molecules, whereas recovery was faster in 5D and even faster in 12D condensates (Fig 2B and C), in line with an increased mobility of phosphomimetic TDP‐43 compared with “unmodified” TDP‐43. Taken together, phosphomimetic S‐to‐D substitutions in the C‐terminal region enhance the liquidity of TDP‐43 condensates, suggesting that phosphorylation in this region might counteract TDP‐43's tendency to form solid, irreversible aggregates.

### C‐terminal phosphomimetic substitutions reduce TDP‐43 aggregation

To address whether phosphorylation indeed counteracts TDP‐43 aggregation, we performed *in vitro* aggregation assays modified from published protocols (Halfmann & Lindquist, 2008; French *et al*, 2019). Under the assay conditions, TEV cleavage of fluorescently labeled TDP‐43‐MBP‐His_6_ yields amorphous TDP‐43 aggregates that can be visualized by confocal microscopy. In contrast to Wt or 12A, the phosphomimetic 5D or 12D proteins formed much smaller and fewer aggregates, respectively (Fig 2D), suggesting that C‐terminal TDP‐43 phosphorylation can efficiently suppress TDP‐43 aggregation. For biochemical characterization of the formed aggregates, we performed semi‐denaturing detergent‐agarose gel electrophoresis (SDD‐AGE) under the same assay conditions, just in the absence of TEV, as MBP‐tagged TDP‐43 aggregates slower than TDP‐43 and distinct oligomeric/polymeric species resistant to 0.5% SDS can be visualized under these conditions (Appendix Fig S3). In comparison to TDP‐43 Wt and 5D, 12D showed reduced and delayed oligomerization and formation of high‐molecular‐weight species (Fig 2E, equal protein input shown in Fig 2F). In contrast, 12A formed SDS‐resistant oligomers/high‐molecular‐weight species at a higher rate, which together with our microscopic images of TDP‐43 condensates (Fig 1F), suggests that C‐terminal alanine substitutions make TDP‐43 more aggregation‐prone. Taken together, C‐terminal phosphomimetic substitutions that mimic the phosphorylation pattern in ALS patients reduce the formation of SDS‐resistant high‐molecular‐weight oligomers and TDP‐43 aggregates *in vitro*.

### Multi‐scale simulations of the TDP‐43 LCD reveal reduced protein‐protein interactions through enhanced solvation of phosphomimetic residues

**Figure EV3:**
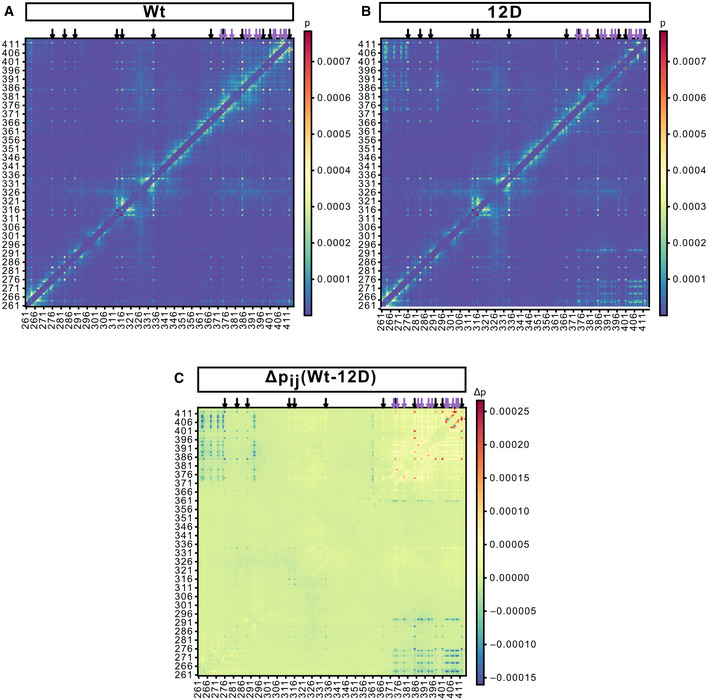
Analysis of contacts in biomolecular condensates formed by the TDP‐43 LCD in coarse‐grained simulations A, BContact maps for Wt (A) and 12D (B) TDP‐43 LCD from simulations with the explicit solvent Martini coarse‐grained model. Residue i and residue j are defined to be in contact if any of the coarse‐grained beads are within 4.5 Å. The relative contact probability is calculated by averaging over all 118 protein chains and the last 5 of 20 μs simulations each. Intra‐chain contacts with the two preceding and following residues are excluded from the analysis. Aromatic residues form prominent contacts and are highlighted by black arrows. For example, looking at the column for F276 and following it upwards one can see that F276 interacts with F276 in other chains and irrespective of the chain, with F283, F289, F313, F316, W334, F367, Y347, W385, F401, and W412. The sites of the phosphomicking S‐to‐D mutations are highlighted by purple arrows. At these sites differences between Wt and 12D LCD can be seen, with Wt forming more contacts close in protein sequence and 12D instead interacting with R268, R272, R275, R293, and R361 further away in the sequence.CDifferences in contact probability *P_i,j_* = *P_i,j_* (Wt) − *P_i,j_* (12D) from simulations with the explicit‐solvent Martini coarse‐grained model. Differences highlight that wild‐type S residues, unlike phosphomicking D residues, favor interactions with residues close in sequence, while demonstrating that most contacts are not affected by the phosphomicking S‐to‐D mutations. Black and purple arrows correspond to aromatic residues and phosphomicking S‐to‐D mutations, respectively.

**Figure 3:**
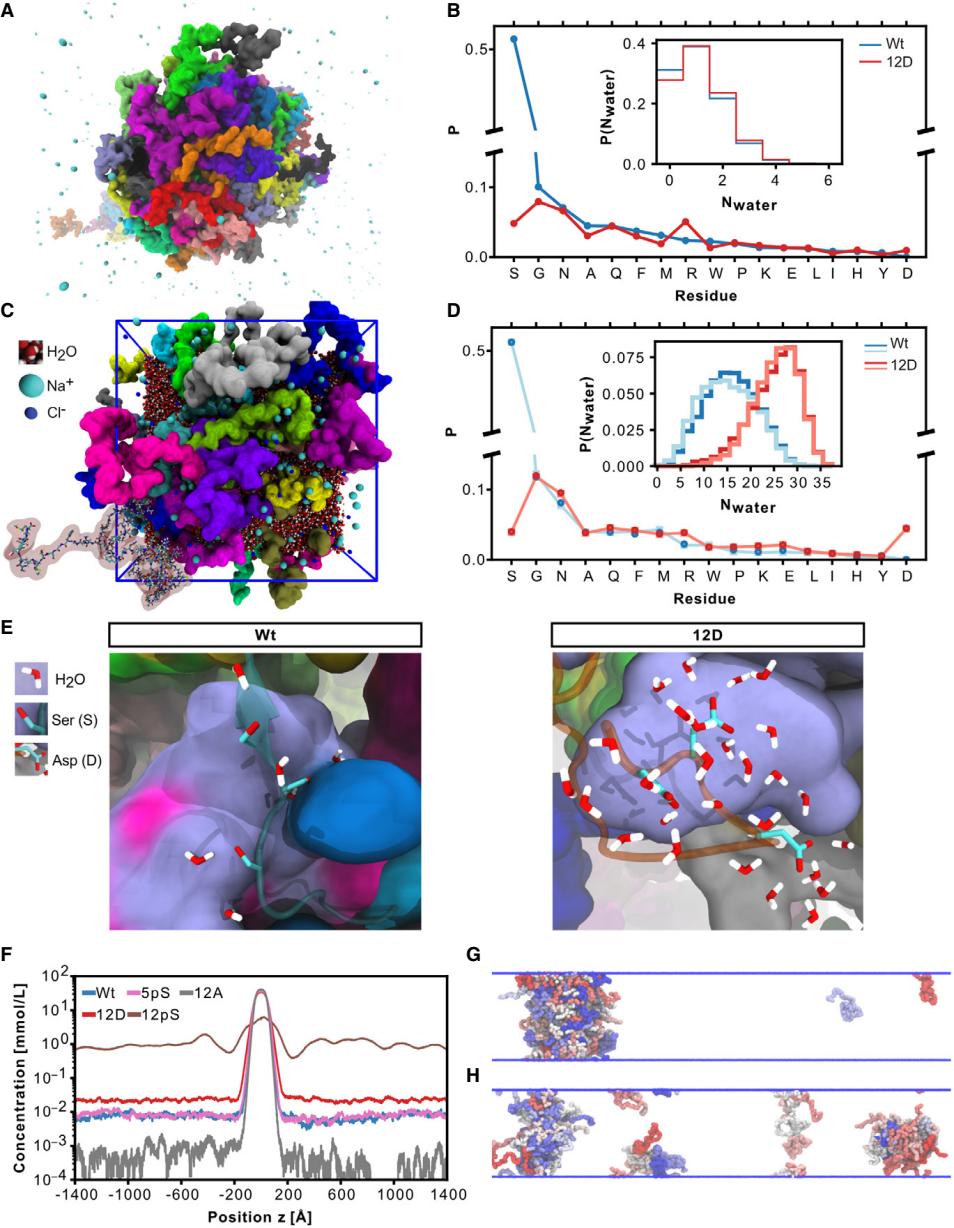
Atomistic and coarse‐grained simulations of TDP‐43 LCD: phosphomimicking residues form fewer protein–protein interactions and more protein–solvent interactions ATDP‐43 LCD phase separates in coarse‐grained simulations with explicit solvent. Condensate of TDP‐43 Wt LCD is shown, protein colored according to chain identity. Water omitted for clarity. Ions shown in cyan.BNormalized probability of protein‐protein contacts by phosphomimicking aspartates in 12D and serines in Wt resolved by amino acid type from coarse‐grained simulations. Error bars smaller than symbols. Inset: Distributions of the number of water molecules within 5 Å of side chains of phosphomimicking aspartates of 12D and corresponding serines in Wt from 15 µs of coarse‐grained molecular dynamics simulations.CAtomistic simulation setup of 32 TDP‐43 LCDs. Different LCD chains shown in different colors in space‐filling representation. For one chain (lower left), a transparent surface reveals its atomic structure as sticks.DNormalized probability of protein–protein contacts by phosphomimicking aspartates in 12D and serines in Wt resolved by amino acid type from atomistic simulations. Two 1 µs simulations are distinguished by color intensity. Inset: distributions of the number of water molecules within 5 Å of the side chains of phosphomimicking aspartates of 12D and the corresponding serines in Wt from atomistic simulations.ERepresentative snapshots of atomistic simulations showing water within 3 Å of (left) Wt S407, S409 and S410 with nearby LCDs in surface representation and (right) 12D D407, D409 and D410. Protein surfaces are colored according to chain identity.FDensity profiles in TDP‐43 LCD condensates (peak at center) coexisting with dilute solutions for Wt, 12D, 5pS, 12pS and 12A from coarse‐grained simulations with the implicit solvent coarse‐grained HPS model.G, HSnapshots of 12D condensate (G) and fragmented 12pS clusters (H) in simulations with the coarse‐grained HPS model. Side view on elongated boxes (blue lines).

To understand the effect of C‐terminal TDP‐43 phosphorylation on phase separation at the molecular level, we used coarse‐grained and atomistic MD simulations of the disordered TDP‐43 LCD (aa. 261–414) with and without phosphomimetic substitutions. In coarse‐grained simulations, we can access the relevant long time and large length scales to characterize phase behavior, while in atomistic simulations we can resolve the interactions of condensates with high resolution and high accuracy (Dignon *et al*, 2018; Pietrek *et al*, 2020; Benayad *et al*, 2021). We found that phosphomimetic substitutions locally reduce protein–protein interactions (Fig EV3) and increase protein–solvent interactions (Fig 3). In the coarse‐grained simulations, the LCD of both TDP‐43 Wt and 12D phase separated spontaneously to form condensates (shown for Wt in Fig 3A and Movie EV4). Yet, phosphomimicking residues are less prone to interact with protein in the phase‐separated condensates and are somewhat more solvated than the corresponding serine residues (Figs 3B and EV3A and B). The aspartate side chains in 12D LCDs engage in partially compensatory interactions with arginines, showing that introduction of charged side chains gives rise to both stabilizing and destabilizing interactions in condensates. Importantly, our simulations are in line with previous studies that have highlighted the importance of aromatic sticker–sticker interactions in driving phase separation of prion‐like domains and the TDP‐43 LCD (Li *et al*, 2018; Schmidt *et al*, 2019; Martin *et al*, 2020).

To characterize the interactions of TDP‐43 LCDs further, we performed atomistic MD simulations of dense protein condensates (Fig 3C, Movie EV5) assembled with hierarchical chain growth (HCG; Pietrek *et al*, 2020) to enhance the sampling of polymeric degrees of freedom. In microsecond dynamics with explicit solvent and a highly accurate atomistic description of molecular interactions (Robustelli *et al*, 2018), we again found serine residues in the Wt protein to be more prone to interact with other protein residues than interacting with solvent (Fig 3D). By contrast, phosphomimicking aspartate side chains bind comparably more water molecules and show an overall reduced tendency for protein‐protein interactions (Fig 3D and E, Appendix Fig S4A). Enhanced side chain solvation is consistent across the 12 phosphomimetic substitution sites (Appendix Fig S4B). The atomistic simulations are consistent with an increase in charge favoring solvated states and thus weakening TDP‐43 condensates.

### Effects of phosphomimicking mutations and phosphorylation on TDP‐43 LCD phase behavior

To characterize possible differences between phosphomicking mutations and phosphorylation, we employed the highly efficient hydrophobicity scale (HPS) coarse‐grained model (Dignon *et al*, 2018). The HPS implicit solvent model enabled us to quantify differences in the phase behavior of TDP‐43 LCD variants. In line with experiments on full‐length TDP‐43 (Fig 1), 12D LCD phase‐separated, but more protein remained in the dilute phase compared with Wt (Fig 3F–H). Indeed, computing the excess free energy of transfer Δ*G*_trans_ from the density profile (Appendix Fig S5D), which reports how favorable it is to move one chain from dilute solution at the saturation density to the dense phase of the condensate, showed that 12D LCDs are less prone to interact with each other in a condensate than Wt LCDs (Appendix Table S1). Loss of local contacts in the C‐terminal region due to phosphomimetic substitutions was only partially compensated by new protein–protein interactions with arginines (Appendix Fig S5A–C), in accordance with coarse‐grained simulations with explicit solvent (Fig EV3C). The 12A substitutions stabilized the TDP‐43 LCD condensates, as expected based on our experiments, with little protein remaining in the dilute phase (Fig 3F). Phosphorylation modulates the stability of LCD condensates in a dose‐dependent way. Attaching five phospho groups (5pS) led to a somewhat less‐dense LCD condensate, but overall the excess free energy of transfer is on par with Wt (Appendix Table S1, Appendix Fig S5D). By contrast, fully phosphorylating all twelve sites (12pS) dissolved the LCD condensate in our simulations, with no clear peak in the density profile (Fig 3F). Overall, the simulations with the HPS model rank the saturation density to form condensates as 12A ≫ Wt ~5pS > 12D ≫ 12pS. The calculations thus predict that (i) phosphorylation may indeed dissolve condensates, and (ii) that phosphorylation may have an even stronger effect than phosphomicking substitutions, due to the larger negative charge of phospho‐serine compared with aspartate.

### C‐terminal phosphomimetic substitutions do not impair nuclear import and RNA regulatory functions of TDP‐43

**Figure EV4:**
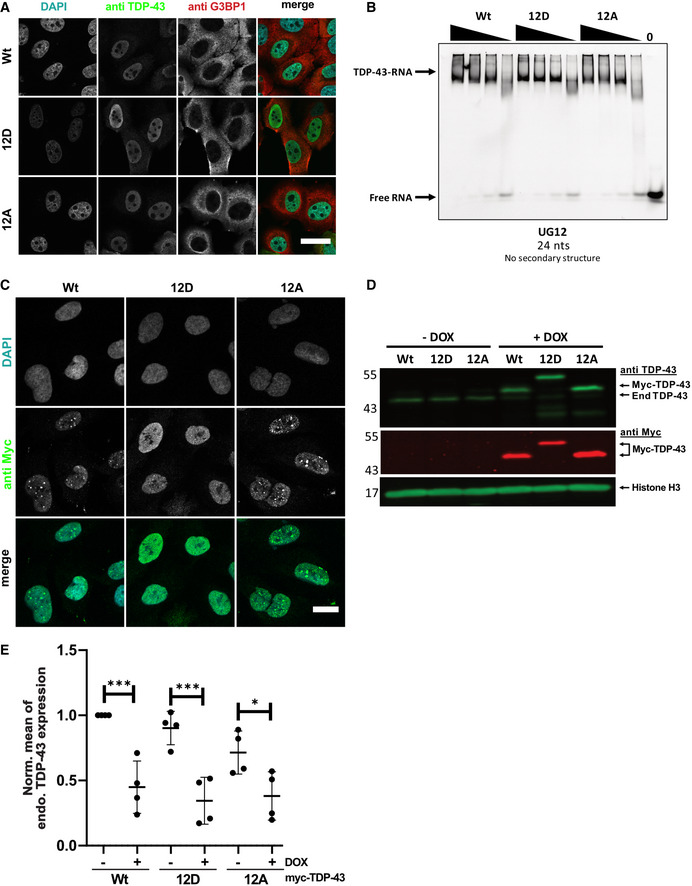
Phosphomimetic substitutions do not alter nuclear localization, UG‐rich RNA binding and autoregulation of TDP‐43 Immunostainings showing nuclear localization of myc‐TDP‐43 Wt, 12D and 12A in HeLa cells. Endogenous TDP‐43 expression was silenced by siRNAs, followed by transient transfection of the indicated siRNA‐resistant myc‐TDP‐43 constructs. After 24 h, localization of TDP‐43 Wt, 12D and 12A variants was visualized by TDP‐43 immunostaining (mouse anti‐TDP‐43 antibody, Proteintech). G3BP1 (rabbit anti‐G3BP1 antibody, Proteintech) and DAPI signal is shown to visualize the cytoplasm and nuclei, respectively. In the merge (right column), DAPI is show in turquoise, TDP‐43 in green, and G3BP1 in magenta. Bar, 30 µm.Electrophoretic mobility shift assay (EMSA) of TDP‐43‐MBP‐His_6_ variants (Wt, 12D and 12A) in a complex with (UG)_12_ RNA.Representative confocal images of U2OS cells stably expressing the indicated myc‐TDP‐43 variants (Wt, 12D and 12A) after siRNA KD of endogenous TDP‐43 and induction of myc‐TDP‐43 expression with doxycycline. Cells were stained with mouse monoclonal anti‐myc 9E10 antibody (IMB protein production facility) and DAPI. For clarity, signals were converted to grey values in the individual channels (upper two rows). In the merge (lower row), DAPI is shown in turquoise) and myc‐TDP‐43 is shown in green. Bar, 20 µm.Western Blot showing the expression levels of myc‐TDP‐43 variants in stable inducible Flp‐In T‐Rex U2OS cell lines before and after addition of doxycycline (dox). Samples were analyzed by SDS–PAGE and Western blot using a rabbit anti‐TDP‐43 N‐term antibody (Proteintech, upper blot), mouse anti‐myc 9E10 antibody (IMB protein production core facility), and rabbit anti‐Histone H3 antibody (Abcam) to detect the loading control Histone H3.Quantification of TDP‐43 autoregulation after dox‐induced expression of myc‐TDP‐43 variants in U2OS cell lines. Values represent the mean ± SD of four independent experimental replicates (*n* = 4) of endogenous TDP‐43 expression levels normalized to Wt (−Dox) condition. *P < 0.0332 and ***P < 0.0002 by one‐way ANOVA with Šídák's multiple comparisons test of TDP‐43 endogenous expression levels, comparing the respective non‐induced (−Dox) and induced (+Dox) lines. Source data are available online for this figure.

**Figure 4:**
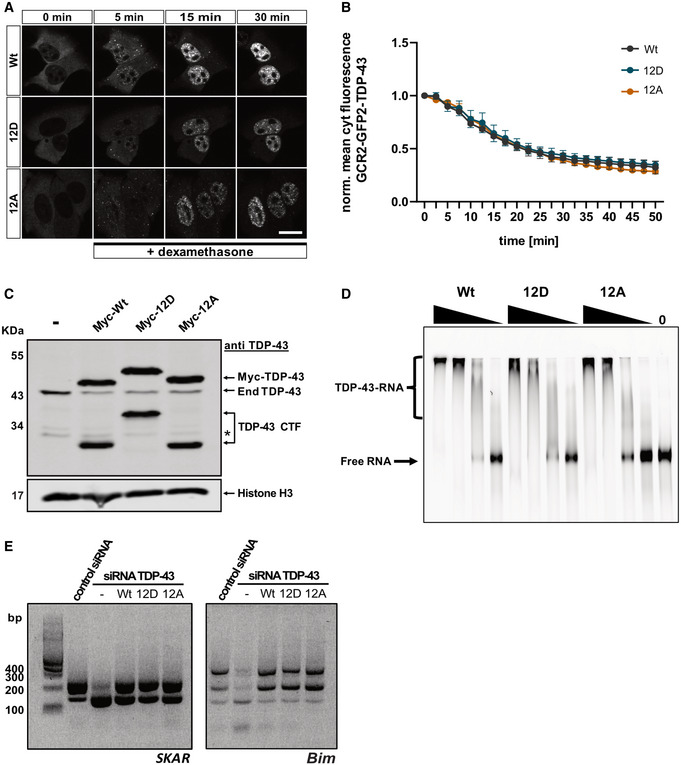
Phosphomimetic substitutions do not alter the rate of TDP‐43 nuclear import and do not impair TDP‐43 autoregulation, RNA‐binding or alternative splicing function Hormone‐inducible nuclear import assay, representative still images of GCR_2_‐EGFP_2_‐TDP‐43 Wt, 12D and 12A before and during import triggered by addition of dexamethasone. Images were live recorded by spinning disc confocal microscopy. Bar, 20 µm.Quantification of the hormone‐inducible nuclear import measured during a total time course of 50 min. Values represent the mean fluorescence intensity of GCR_2_‐EGFP_2_‐TDP‐43 in the cytoplasm for three independent replicates ± SEM (≥ 42 cells per condition).Phosphomimetic 12D TDP‐43 is competent in autoregulating TDP‐43 expression. SDS–PAGE followed by TDP‐43 Western blot showing downregulation of endogenous TDP‐43 through autoregulation (*60*) after 48 h expression of Wt, 12D and 12A variants in HeLa cells. TDP‐43 was detected using rabbit anti‐TDP‐43 C‐term antibody (Proteintech), Histone H3 (rabbit anti‐Histone H3 antibody, Abcam) was visualized as a loading control. * denotes an unspecific band.Electrophoretic mobility shift assays (EMSA) of TDP‐43‐MBP‐His_6_ variants (Wt, 12D and 12A) in a complex with TDP‐43 autoregulatory RNA binding site (*60*). All TDP‐43 variants form TDP‐43‐RNA complexes equally well.Splicing analysis by RT–PCR of known TDP‐43 splice targets (*SKAR* exon 3 and *Bim* exon 3) in HeLa cells. Silencing of endogenous TDP‐43 by siRNA leads to altered splice isoforms of *SKAR* and *Bim* (second vs first lane). These splicing alterations can be rescued by re‐expression of TDP‐43 Wt, but also 12D or 12A variants, demonstrating that phosphomimetic TDP‐43 is fully competent in regulation splicing of these TDP‐43 splice targets. Source data are available online for this figure.

Next, we turned to cellular experiments to investigate how C‐terminal TDP‐43 phosphorylation affects the behavior and function of TDP‐43 in cells. As TDP‐43 hyperphosphorylation is found in the disease state, it seems possible that this PTM has detrimental effects on the protein and contributes to mislocalization and/or malfunction of TDP‐43, thus driving neurodegeneration. To address this possibility, we transiently expressed different myc‐tagged TDP‐43 variants (Wt, 12D, 12A) in HeLa cells and analyzed their intracellular localization, nuclear import and RNA processing functions. All three TDP‐43 variants showed a predominantly nuclear steady‐state localization (Fig EV4A). We also compared their nuclear import rates in a hormone‐inducible reporter assay by live cell imaging (Hutten *et al*, 2020). In this assay, a protein‐of‐interest harboring a nuclear localization signal (NLS) is fused to a tandem EGFP and two hormone binding domains of the glucocorticoid receptor (GCR), which retains the reporter protein in the cytoplasm. Upon addition of a steroid hormone (dexamethasone), the reporter protein is released from the cytoplasm and imported into the nucleus, by virtue of the NLS in the protein‐of‐interest. We examined reporters containing the different TDP‐43 variants (Wt, 12D, 12A) and found that their import rates were indistinguishable (Fig 4A and B).

To assess whether hyperphosphorylated TDP‐43 shows functional impairments in RNA processing, we first assessed its ability to autoregulate its own levels when transiently overexpressed in HeLa cells (Ayala *et al*, 2011; Avendano‐Vazquez *et al*, 2012). However, endogenous TDP‐43 was downregulated to the same degree by all three myc‐TDP‐43 variants (Fig 4C), indicating that hyperphosphorylated TDP‐43 can normally bind to its own 3′UTR and autoregulate its own levels. In line with these findings, recombinant TDP‐43 Wt, 12D and 12A showed comparable RNA binding in electrophoretic mobility shift assays (EMSAs) with *in vitro* transcribed RNA comprised of the autoregulatory TDP‐43 binding site (Fig 4D) or synthetic (UG)_12_ RNA (Fig EV4B). Second, we examined splicing of two known TDP‐43 splice targets that get mis‐spliced upon loss of TDP‐43 (Tollervey *et al*, 2011; Fiesel *et al*, 2012). After siRNA‐mediated silencing of endogenous TDP‐43 expression and re‐expression of siRNA‐resistant myc‐TDP‐43 Wt, 12D or 12A (Appendix Fig S6A), splicing of *SKAR* and *Bim* exon 3 were fully restored by all three TDP‐43 variants (Fig 4E), indicating normal function of phosphomimetic TDP‐43 in splicing regulation. Normal nuclear localization and autoregulation of TDP‐43 were also replicated in a cellular system that avoids high overexpression and has homogenous expression levels, namely stable inducible Flp‐In U2OS cell lines that express the different myc‐TDP‐43 variants (Wt, 12D and 12A) after overnight doxycycline addition (Fig  EV4C–E, in Fig EV4C endogenous TDP‐43 was silenced with siRNAs, see Appendix Fig S6C). In conclusion, even though an effect on other RNA targets/RNA processing events or intracellular transport in other cell types cannot be excluded, our data suggest that C‐terminal TDP‐43 hyperphosphorylation is not primarily responsible for cytosolic mislocalization or impaired RNA regulatory functions of TDP‐43 in disease.

### Phosphorylation suppresses recruitment of TDP‐43 into stress‐induced MLOs

**Figure 5:**
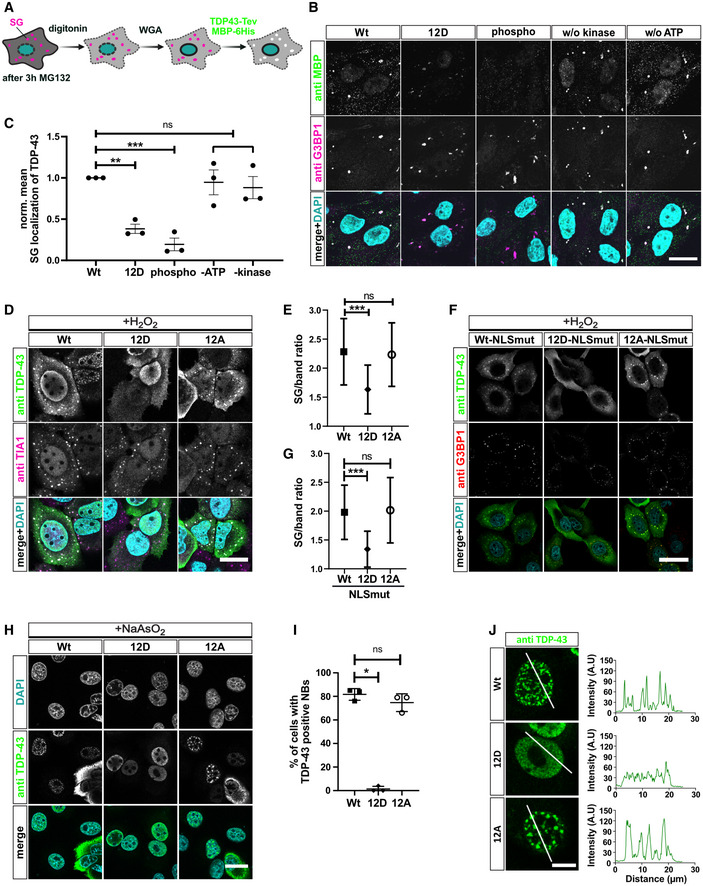
Phosphorylation and phosphomimetic substitutions reduce recruitment of TDP‐43 into stress‐induced membrane‐less organelles Scheme of stress granule (SG) recruitment assay in semi‐permeabilized cells.Reduced SG association of TDP‐43 by 12D mutations or *in vitro* phosphorylation. Bar, 20 µm.Quantification of TDP‐43‐MBP‐His_6_ mean fluorescence intensity in SGs normalized to Wt ± SEM of three independent experimental replicates (*n* = 3; ≥ 10 cells; ≥ 46 SGs each). **P < 0.0021 and ***P < 0.0002 by one‐way ANOVA with Dunnett's multiple comparison test to Wt.SG recruitment of TDP‐43 variants in intact HeLa cells in absence of endogenous TDP‐43. After TDP‐43 silencing and expression of myc‐TDP‐43 Wt, 12D and 12A variants, SGs were induced by H_2_O_2_ treatment and SG recruitment of TDP‐43 was monitored by TDP‐43 and TIA1 immunostaining. For clarity, signals were converted to grey values in the individual channels (upper two rows). In the merge (lower row), nuclei were stained in DAPI (turquoise), TDP‐43 (green) and TIA‐1 (magenta). Bar, 25 µm.Quantification of TDP‐43 in SGs versus cytoplasm ± SD of two independent experimental replicates (*n* = 2; ≥ 62 cells; ≥ 234 SGs each). ***P < 0.0002 by Krustal–Wallis test with Dunn's multiple comparison test to Wt.SG recruitment of different TDP‐43‐NLSmut variants in intact HeLa cells in the absence of endogenous TDP‐43. After TDP‐43 silencing and expression of NLSmut Wt, 12D and 12A variants, SGs were induced by H_2_O_2_ treatment and SG recruitment of TDP‐43 was monitored by TDP‐43 and G3BP1 immunostaining. For clarity, signals were converted to grey values in the individual channels (upper two rows). In the merge (lower row), nuclei were stained in DAPI (turquoise), TDP‐43 (green) and G3BP1 (red). Bar, 40 µm.Quantification of TDP‐43‐NLS mutants in SGs versus band around SGs of two independent replicates ± SD. ***P < 0.0002 by Kruskal–Wallis test with Dunn's multiple comparison test to Wt (≥ 56 cells; ≥ 315 SGs per condition).Recruitment of TDP‐43 into arsenite‐induced nuclear bodies (NBs) in HeLa cells. After TDP‐43 silencing and expression myc‐TDP‐43 Wt, 12D and 12A, NBs were induced by sodium arsenite treatment and NB recruitment of TDP‐43 was monitored by TDP‐43 immunostaining. Bar, 20 µm.Percentage of cells with TDP‐43 in NBs ± SD of three independent experimental replicates (*n* = 3). *P < 0.0332 by Kruskal–Wallis test with Dunn's multiple comparison test to Wt.Intensity profiles (right) of TDP‐43 Wt, 12D and 12A variants (green) along white lines (left). Bar, 10 µm.

**Figure EV5:**
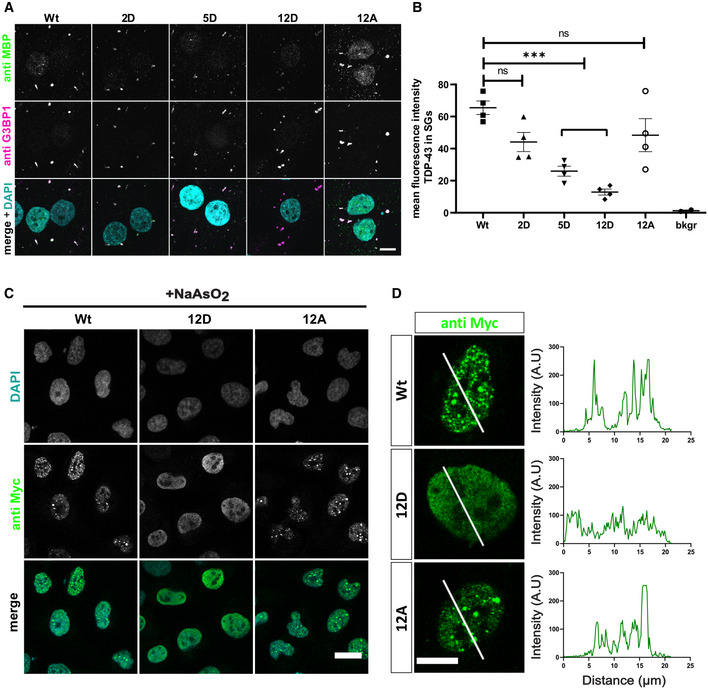
Phosphomimetic S‐to‐D substitutions reduce association of TDP‐43 with stress granules and nuclear stress bodies Association of TDP‐43 with stress granules (SGs) in semi‐permeabilized HeLa cells is suppressed by phosphomimetic (2D, 5D and 12D) mutations in comparison to TDP‐43 Wt and 12A. SGs and TDP‐43‐MBP‐His_6_ were visualized by G3BP1 and MBP immunostaining, respectively. For clarity, signals were converted to grey values in the individual channels (upper two rows). In the merge (lower row), G3BP1 is shown in magenta, TDP‐43‐MBP‐His_6_ in green, white pixels indicate colocalization. Nuclei were counterstained with DAPI (turquoise). Bar, 10 µm.Quantification of the mean fluorescence intensity of TDP‐43‐MBP‐His_6_ in SGs normalized to Wt for four independent replicates ± SEM, ***P < 0.0002 defined by 1‐way ANOVA with Dunnett's multiple comparison test (≥ 10 cells; ≥ 46SGs per condition). Bkgr = background fluorescence in the green channel.Representative confocal images of U2OS cells stably expressing the indicated myc‐TDP‐43 variants (Wt, 12D and 12A) after siRNA KD of endogenous TDP‐43 and induction of myc‐TDP‐43 expression with doxycycline, followed by sodium arsenite stress (2 h) to induce nuclear stress bodies (NBs). Cells were stained with mouse monoclonal anti‐myc 9E10 antibody (IMB protein production facility) and DAPI. For clarity, signals were converted to grey values in the individual channels (upper two rows). In the merge (lower row), DAPI is shown in turquoise and myc‐TDP‐is shown in green. Bar, 20 µm.Intensity profiles (right) of myc‐TDP‐43 Wt, 12D and 12A variants (green), expressed in Flp‐In T‐Rex USOS stable cell lines, along white lines (left). Bar, 10 µm.

Finally, we investigated how C‐terminal TDP‐43 phosphorylation affects TDP‐43 condensation in cellular MLOs. First, we used a quantitative assay to measure SG association of recombinant proteins under controlled conditions in semi‐permeabilized HeLa cells (Hutten & Dormann, 2020) (Fig 5A). In line with our *in vitro* condensation experiments, increasing the number of phosphomimetic S‐to‐D substitutions caused a gradual decrease in SG association of TDP‐43 (Fig EV5A and B). *In vitro* phosphorylated TDP‐43 showed a similar or even stronger reduction in SG association as the 12D protein (Fig 5B and C), demonstrating that the phosphomimetic substitutions and phospho‐groups introduced by a kinase have similar effects on SG association of TDP‐43. Second, we expressed the different TDP‐43 variants in intact HeLa cells to analyze their recruitment into stress‐induced MLOs. To this end, we silenced endogenous TDP‐43 expression using siRNA (Appendix Fig S6A and B) and then re‐introduced siRNA‐resistant myc‐tagged TDP‐43 Wt, 12D or 12A, thus avoiding oligomerization with endogenous TDP‐43 via the N‐terminal domain (Afroz *et al*, 2017). Short term oxidative stress treatment with H_2_O_2_ caused a partially cytosolic relocalization of TDP‐43 and led to recruitment of TDP‐43 Wt and 12A, but significantly reduced recruitment of the 12D mutant into TIA‐1‐positive SGs (Fig 5D and E, see Appendix Fig S6D for control staining of untransfected cells). Similar results were obtained for nuclear import‐deficient TDP‐43 (Fig 5F and G) that was strongly mislocalized to the cytoplasm due to point mutations in the nuclear localization signal (NLSmut; Appendix Fig S7). Finally, we examined recruitment of TDP‐43 into arsenite‐induced nuclear bodies (NBs) (Wang *et al*, 2020) and found that TDP‐43 Wt and 12A were readily recruited into stress‐induced NBs, while the phosphomimetic 12D protein remained dispersed in the nucleoplasm (Fig 5H–J, see Appendix Fig S6E for control staining of untransfected cells). TDP‐43 12D also remained completely dispersed in the stable inducible U2OS cell lines treated with arsenite, whereas TDP‐43 WT and 12A localized in NBs upon arsenite treatment (Fig EV5C and D). Taken together, phosphomimetic substitutions that mimic disease‐linked phosphorylation of TDP‐43 suppress the localization of TDP‐43 in phase‐separated MLOs that could be condensation sites for pathological TDP‐43 aggregation.

### Phosphomimetic substitutions enhance TDP‐43 solubility and suppress SG recruitment in primary neurons

**Figure 6:**
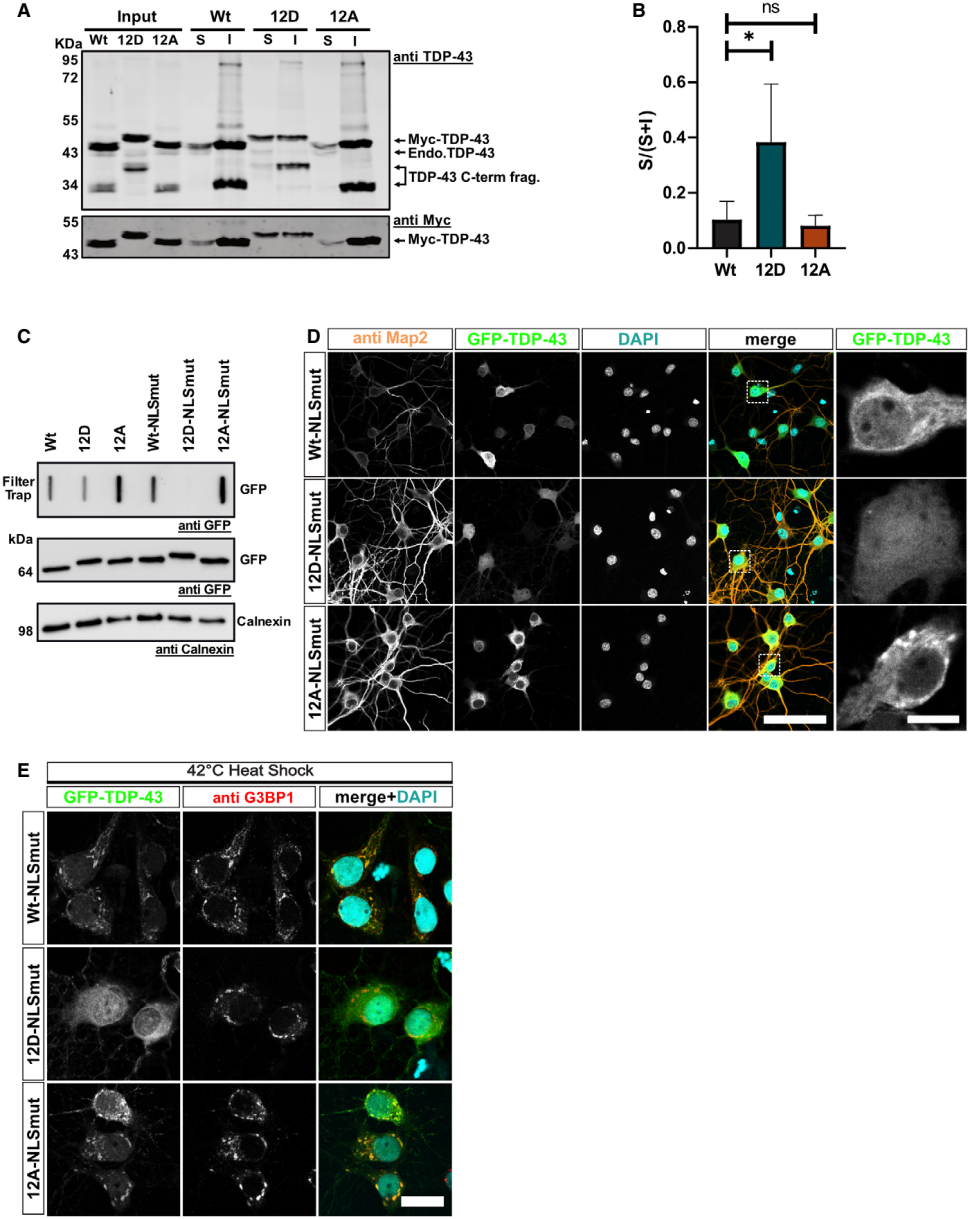
Phosphomimetic substitutions enhance TDP‐43 solubility in HeLa cells and primary neurons Biochemical fractionation into RIPA‐soluble (S) and RIPA‐insoluble (I) fractions to analyze solubility of the different myc‐TDP‐43 variants (Wt, 12D and 12A) expressed in HeLa cells for 48 h. TDP‐43 was detected by TDP‐43 Western blot (upper blot, rabbit anti‐TDP‐43 C‐term, Proteintech) and Myc Western blot (lower blot, mouse anti‐Myc 9E10).Quantification of myc‐TDP‐43 variants (Wt, 12D and 12A) in (S) versus (I) fractions extracted from TDP‐43 Western blots of four independent replicates ± SD, plotted as S/(S + I). *P < 0.0332 by one way ANOVA with Dunnett's multiple comparison test to Wt.RIPA‐insoluble material of the indicated EGFP‐tagged TDP‐43 variants (± NLS mutation) expressed in primary cortical neurons analyzed by filter‐trap assay.Primary hippocampal neurons expressing EGFP‐TDP‐43 Wt, 12D or 12A with additional NLS mutation. Bar, 80 µm. Right: zoomed images of white squares (TDP‐43 signal). Bar, 10 µm.SG recruitment of EGFP‐TDP‐43 NLS mutant variants (Wt, 12D, 12A) in primary hippocampal neurons. SG formation was induced by 1 h heat shock at 42°C. SGs and TDP‐43 were monitored by G3BP1 antibody staining and EGFP fluorescence, respectively. For clarity, signals were converted to grey values in the individual channels (first two columns). In the merge (third column), EGFP‐TDP‐43 shown in green, G3BP1 in red and nuclei (DAPI staining) in turquoise. Bar, 20 µm. Source data are available online for this figure.

To further support the idea that phosphorylation enhances the solubility of TDP‐43 and counteracts its aggregation propensity in cells, we expressed the different myc‐tagged TDP‐43 variants in HeLa cells and performed a biochemical fractionation into a RIPA‐soluble (S) and RIPA‐insoluble (I) fraction. Indeed, the 12D protein had a significantly higher S/(S + I) ratio compared with the Wt and 12A proteins (Fig 6A and B). We also expressed EGFP‐tagged TDP‐43 Wt, 12D, 12A or the corresponding NLS‐mutant cytosolic versions in primary rat neurons (see Appendix Fig S8 and Fig 6D for subcellular localization, which was unaltered by the phosphomimetic mutations). We then probed for RIPA‐insoluble high‐molecular‐weight material in a filter trap assay. Both the nuclear and the cytosolic 12D proteins showed a strong reduction in the amount of RIPA‐insoluble TDP‐43 in the transduced neurons (Fig 6C). Confocal microscopy of transduced neurons revealed a completely dispersed localization of the NLS‐mutant 12D protein, whereas TDP‐43 Wt and 12A showed a more granular, condensed pattern in the neuronal cytoplasm (Fig 6D). Moreover, NLS mutant TDP‐43 Wt and 12A were readily recruited into G3BP1‐positive SGs induced by heat shock in primary rat neurons, whereas TDP‐43 12D was not (Fig 6E). Thus, we conclude that phosphomimetic substitutions mimicking disease‐linked C‐terminal hyperphosphorylation reduce TDP‐43's tendency to condense into MLOs and to become insoluble in neurons. Based on these findings, we speculate that TDP‐43 phosphorylation might be a cellular response to counteract pathological TDP‐43 aggregation.

## Discussion

C‐terminal TDP‐43 phosphorylation is a long‐recognized pathological hallmark in ALS and FTD (Hasegawa *et al*, 2008; Inukai *et al*, 2008; Neumann *et al*, 2009; Kametani *et al*, 2016). Against previous expectations, we now show that TDP‐43 phosphorylation, and in particular phosphomimetic mutations mimicking the phosphorylation pattern in ALS/FTD (Hasegawa *et al*, 2008; Kametani *et al*, 2016), strongly suppress TDP‐43 phase separation and aggregation both *in vitro* and in cells. Our data are in line with two previous studies that examined C‐terminal fragments of TDP‐43 with phosphomimetic 2D or 5D/E mutations and observed a reduced aggregation propensity and toxicity in cell lines and *Drosophila* (Brady *et al*, 2011; Li *et al*, 2011). Even though phosphomimetic mutations do not always recapitulate the effects of phosphorylation on protein‐protein interactions (Yaffe *et al*, 1997; Durocher *et al*, 1999), our *in vitro* data with purified proteins show that the phosphomimetic 12D variant has a similar condensation behavior as CK1δ‐phosphorylated TDP‐43. We would like to point out that phosphomimetic mutations are an experimental under‐appreciation of true charge, as aspartate has a net charge of −1, whereas phosphorylation has a net charge of −2. In line, our simulations show that 12S‐p disrupts phase separation of the TDP‐43 LCD more strongly than the phosphomimetic 12D mutations (Fig 3F–H). It seems possible that the number of phosphorylation sites, but not their exact position, is critical for the suppression of TDP‐43 condensation, which would indicate that multisite phosphorylation may regulate TDP‐43 phase separation through bulk electrostatics, as previously shown for other proteins (Serber & Ferrell, 2007; Strickfaden *et al*, 2007). Indeed, two recent studies showed that the net charge of IDR‐containing RBPs tunes their driving force for assembly (preprint: Crabtree *et al*, 2020; preprint: Bremer *et al*, 2021). For instance, reducing the net charge of the disordered region of Ddx4 promotes its phase separation (preprint: Crabtree *et al*, 2020), and increasing the net charge of the low complexity region of hnRNP‐A1 reduces its phase separation, likely due to repulsive electrostatic long‐range interactions (preprint: Bremer *et al*, 2021). At physiological pH, TDP‐43 has a net charge of −4.1, phosphomimetic 12D TDP‐43 has a net charge of −16.1 and 12× phosphorylated TDP‐43 has a net charge of −28.1. In line with the aforementioned studies, it seems likely that the strong increase in negative net charge in phosphomimetic/phosphorylated TDP‐43 is responsible for the reduced propensity to self‐assemble into condensates.

Various modes of TDP‐43 assembly have been proposed, including homotypic interactions of an α‐helical structure in the conserved region (CR) of the LCD (Conicella *et al*, 2016, 2020) and interactions between aromatic/aliphatic residues in the LCD (Li *et al*, 2018; Schmidt *et al*, 2019; Laurents *et al*, 2021). All phosphomimetic mutations examined in our study are outside of the α‐helix/CR (aa. 321–360; Fig 1D), hence they are unlikely to interfere with helix‐helix interactions. In line with this hypothesis, the contact maps extracted from the simulations of the TDP‐43 LCD show that most interactions, including aromatic interactions, are not strongly affected by C‐terminal phosphomimetics and that mainly interactions of serines with nearby residues are reduced. However, more work is required to understand how PTMs on TDP‐43 affect aromatic “sticker”–“sticker” interactions on the molecular scale. C‐terminal phosphorylation may also affect amyloid‐like fibril formation: A recent cryo‐EM structure of solid TDP‐43 LCD fibrils showed that several C‐terminal serines are buried inside the fibril structure (Li *et al*, 2021), hence phosphorylation could disrupt the amyloid fibril structure, in line with our experimental findings that TDP‐43 aggregate formation is strongly reduced by phosphomimetic substitutions. We therefore speculate that TDP‐43 phosphorylation might be a protective cellular mechanism that counteracts aberrant TDP‐43 phase transitions and renders TDP‐43 more dynamic and liquid‐like by reducing C‐terminal LCD‐LCD interactions through negatively charged, highly hydrated phospho‐groups.

Under what conditions C‐terminal TDP‐43 phosphorylation arises in cells and which form of TDP‐43 (soluble, phase separated, aggregated) is phosphorylated is still unknown. Interestingly, we and others previously found that C‐terminal TDP‐43 phosphorylation follows TDP‐43 insolubility, suggesting that phosphorylation arises downstream of TDP‐43 aggregation (Dormann *et al*, 2009; Brady *et al*, 2011; Zhang *et al*, 2019). In line with these findings, treatment of neuron‐like cells with amyloid‐like particles triggers a solidification of initially liquid‐like cytoplasmic TDP‐43 droplets, along with C‐terminal TDP‐43 phosphorylation (Gasset‐Rosa *et al*, 2019). Why phosphorylated TDP‐43 aggregates nevertheless persist and are not readily disassembled after phosphorylation remains to be investigated. Further research into the functional consequences of C‐terminal TDP‐43 phosphorylation, e.g., how it affects global protein or RNA interactions, TDP‐43 stability or the introduction of additional PTMs, is needed to understand the role of TDP‐43 phosphorylation in physiology and pathology.

Several other studies on TDP‐43 phosphorylation at first glance contrast our findings. Overexpression of various TDP‐43 kinases in cell or animal models was shown to promote TDP‐43 aggregation and neurotoxicity (Choksi *et al*, 2014; Liachko *et al*, 2014; Nonaka *et al*, 2016; Taylor *et al*, 2018). Based on these studies, inhibition of TDP‐43 phosphorylation by kinase inhibitors has been proposed as a potential therapeutic strategy for ALS (Liachko *et al*, 2013; Salado *et al*, 2014; Martinez‐Gonzalez *et al*, 2020). A possible explanation for the discrepant findings could be that kinase overexpression has pleiotropic effects that may cause TDP‐43 aggregation and neurotoxicity independent of TDP‐43 phosphorylation. Our data exclude such indirect effects, as they rely on experiments with purified components, MD simulations and defined phosphomimetic constructs rather than modulation of kinase levels/activity. Furthermore, our results suggest that beneficial effects seen with kinase inhibitors are likely not the direct consequence of reduced TDP‐43 phosphorylation, but rather mediated by other mechanisms.

An alternative scenario that we cannot exclude is that reduced TDP‐43 condensation due to hyperphosphorylation may have negative consequences by disturbing essential functions of TDP‐43 that depend on its capacity to phase separate or solidify, e.g., certain DNA/RNA processing steps or recruitment of TDP‐43 into cytoprotective NBs (Wang *et al*, 2020) or other MLOs. In support of this hypothesis, a deep mutagenesis study recently found that aggregating TDP‐43 variants decrease toxicity in yeast, whereas dynamic, liquid‐like variants enhance toxicity (Bolognesi *et al*, 2019), so further work is needed to investigate this possible scenario. However, our data clearly show that some essential RNA processing functions (autoregulation and regulation of certain splicing events), RNA‐binding and nuclear localization/import of TDP‐43 are not affected by C‐terminal hyperphosphorylation, at least in HeLa and U2OS cells, and therefore do not depend on TDP‐43's phase separation and solidification capacity. In line with our findings, an earlier study found that phase separation‐deficient TDP‐43 remains competent in splicing regulation (Schmidt *et al*, 2019).

Of note, abnormal PTMs are a common theme in neurodegenerative disorders, e.g., Tauopathies linked to pathological Tau aggregation (Morris *et al*, 2015; Alquezar *et al*, 2020). Interestingly, even though hyperphosphorylation is generally believed to trigger Tau aggregation, site‐specific phosphorylation in the microtubule‐binding region of Tau was recently shown to inhibit, rather than promote Tau fibrillization and seeding (Haj‐Yahya *et al*, 2020). We now show that C‐terminal TDP‐43 phosphorylation as detected on ALS/FTD inclusions has a similar inhibitory effect on TDP‐43 aggregation, underscoring the idea that aberrant PTMs detected on pathological inclusions may not necessarily all be drivers of protein aggregation, but could also have protective, anti‐aggregation effects that are later‐on overruled by other pathogenic mechanisms.

## Materials and Methods

### cDNA constructs

#### Bacterial expressing constructs

TDP‐43 carrying mutations in serine 409 and 410, either to aspartate (2D) or to alanine (2A), were generated by site‐directed mutagenesis using Q5 high fidelity DNA polymerase (NEB) using primers containing the mutations S409D/410D and S409A/410A and pJ4M TDP‐43‐TEV‐MBP‐His_6_ vector as a template. Expression constructs with 5 or 12 serine substitutions (5D, 5A, 12D and 12A) were generated using synthetic double‐stranded DNA fragments (gBlocks Gene Fragments, IDT) containing the respective mutations, cloned into PstI and XhoI sites of the pJ4M TDP‐43‐TEV‐MBP‐His_6_‐backbone.

#### Mammalian expressing constructs

To generate an expressing construct coding for Myc‐hTDP‐43, the coding sequence of hTDP‐43 was PCR amplified from pEGFP‐C1‐hTDP‐43 (Ederle *et al*, 2018), including a Myc coding sequence in the forward PCR primer, and cloned into a pcDNA5‐FRT‐TO‐backbone using XhoI and BamHI restriction sites. Note, that the hTDP‐43 template includes a resistance to TARDBPHSS118765 siRNA (Invitrogen) used to silence endogenous TDP‐43 (see “siRNA‐mediated knockdown of TDP‐43”). For generation of the TDP‐43 12D and 12A constructs, synthetic gBlocks (IDT) harboring the respective mutations were previously cloned into the NdeI and BamHI sites of the pEGFP‐C1‐hTDP‐43 vector. In constructs carrying mutations in the NLS of TDP‐43 (mNLS), amino acids 82–84 as well as 95, 97 and 98 were exchanged for alanine (pEGFP‐hTDP‐43 mNLS). Then, the mNLS region was transferred from the pEGFP‐TDP‐43 mNLS template to the pcDNA5‐FRT‐TO‐Myc‐hTDP‐43, 12D and 12A vectors via the restriction enzymes XhoI and NdeI. To generate the GCR_2_‐EGFP_2_‐TDP‐43 12D and 12A constructs, the respective coding sequences were PCR amplified and inserted into GCR_2_‐EGFP_2_‐backbone using EcoRV and BamHI. To allow for lentiviral packaging and subsequent neuronal transduction, coding sequences of TDP‐43 Wt, 12D and 12A were subcloned into the FhSynW backbone in frame with mEGFP (May *et al*, 2014).

### HeLa cell culture, transient transfection and stress treatment

HeLa cells were grown in DMEM high glucose GlutaMAX (Invitrogen) supplemented with 10% fetal bovine serum (FBS) and 10 µg/ml gentamicin and incubated in a humidified chamber with 5% CO_2_ at 37°C. cDNA transfections were performed using Lipofectamine 2000 (Thermo) in culture medium without gentamicin and medium was exchanged after 4 to 6 h to avoid cellular stress by the transfection reagent. Note, that for equal transfection efficiency different amounts of DNA were transfected for the different constructs (for 12D: 100%; for Wt and 12A: 75% + 25% empty vector DNA). For immunostaining cells were fixed after ~24 h, hydrogen peroxide (H_2_O_2_; 1 mM) treatment was carried out for 2 h, MG132 (10 µM) treatment for 2.5–3 h and sodium arsenite (0.5 mM) treatment for 45 min.

### Flp‐In T‐Rex U2OS cell culture and stress treatment

Inducible U2OS cell lines stably expressing myc‐hTDP‐43 variants (Wt, 12D and 12A) were generated using the Flp‐In T‐Rex system. Flp‐In T‐Rex U2OS cells (gift from A. Lamond lab) were cotransfected with pcDNA5‐FRT‐TO‐hTDP‐43 (Wt, 12D or 12A) and pOG44 Flp recombinase expression plasmids, followed by selection with Hygromycin (150 µg/ml) and Blasticidin (15 µl/ml). After expansion of surviving single cell colonies, myc‐TDP‐43 expression was induced by doxycycline (dox) addition for 24 h, using 0.005 µg/ml dox for myc‐TDP‐43 Wt and 0.25 µg/ml dox for TDP‐43 12D and 12A, in order to yield similar protein expression levels. To induce nuclear stress bodies, cells were treated with sodium arsenite (0.5 mM) for 2 h.

### Neuronal cell culture, lentiviral packaging and stress treatment

Primary hippocampal and cortical neuronal cultures were prepared from embryonic day 19 rats as described in detail previously (Guo *et al*, 2018). In brief, neocortex and hippocampus were dissected, followed by enzymatic dissociation and gentle trituration. For immunofluorescence experiments, hippocampal neurons (85,000 cells/ml) were plated on poly‐d‐lysine‐coated glass coverslips (VWR) in 12‐well plates (Thermo Fisher) and cultured in Neurobasal medium (Thermo Fisher) supplemented with 2% B27 (Thermo Fisher), 1% Penicillin–Streptomycin (Thermo Fisher), 0.5 mM l‐glutamine (Thermo Fisher) and 12.5 µM glutamate (Thermo Fisher). Both, cortical and hippocampal neurons, were transduced on day *in vitro* (DIV) 5.

Cortical neurons (250,000 cells/ml) used for filter trap assays were plated on poly‐d‐lysine‐coated six‐well plates and cultured in Neurobasal medium containing 2% B27, 1% Penicillin–Streptomycin and 0.5 mM l‐glutamine.

Lentiviral packaging was performed by seeding HEK293FT cells (Thermo Fisher) of low passage number into three 10 cm dishes per construct (5 × 10^6^ cells/dish). Cells were plated in DMEM, high glucose, GlutaMAX (Thermo Fisher) supplemented with 10% FBS (Sigma), 1% Penicillin–Streptomycin (Thermo Fisher) and 1% non‐essential amino acids (Thermo Fisher). On the following day, cells were co‐transfected with 18.6 µg transfer vector (FhSynW‐mEGFP‐hTDP‐43, FhSynW‐mEGFP‐hTDP‐43 [12D], FhSynW‐mEGFP‐hTDP‐43 [12A], FhSynW‐mEGFP‐hTDP‐43‐mNLS, FhSynW‐mEGFP‐hTDP‐43‐mNLS [12D] and FhSynW‐mEGFP‐hTDP‐43‐mNLS [12A]), 11 µg pSPAX2 and 6.4 µg pVSVG using Lipofectamine 2000 (Thermo Fisher). The transfection media was replaced by plating media supplemented with 13 mg/ml bovine serum albumin (BSA, Sigma) on the next day. Lentivirus from the cell supernatant was collected 24 h later by ultracentrifugation with a Sw28 rotor (Beckman Coulter; 64,100 *g*, 2 h, 4°C). Finally, lentiviral particles were resuspended in Neurobasal media (Thermo Fisher), stored at −80°C and used for lentiviral transduction by adding to neuronal culture media upon thawing. Neurons were kept in culture for 4 additional days after transduction on DIV5 (DIV5 + 4). To induce SGs, heat shock was carried out by incubating neurons for 1 h at 42°C in a cell culture incubator.

### Recombinant protein expression and purification

#### TDP‐43‐TEV‐MBP‐His_6_

All TDP‐43‐MBP‐His_6_ variants were purified according to (Wang *et al*, 2018) with minor adaptations. First, expression of proteins was performed in *E. coli* BL21‐DE3 Rosetta 2 using 0.5 mM IPTG and grown overnight at 16°C. Next, cells were resuspended in lysis buffer (20 mM Tris pH 8, 1 M NaCl, 10 mM imidazole, 10% (v/v) glycerol, 4 mM β‐mercaptoethanol and 1 µg/ml each of aprotinin, leupeptin hemisulfate and pepstatin A) supplemented with 0.1 mg/ml RNase A, and lysed using lysozyme and sonication. Subsequently, the protein was purified by Ni‐NTA agarose (Qiagen) and eluted with lysis buffer containing 300 mM imidazole. For all TDP‐43‐MBP‐His_6_ variants, a final size exclusion chromatography (SEC; Hiload 16/600 Superdex 200 pg, GE Healthcare) purification step was performed in purification buffer (20 mM Tris pH 8, 300 mM NaCl, 10% (v/v) glycerol supplemented with 2 mM TCEP), in order to separate TDP‐43‐MBP‐His_6_ from protein aggregates and contaminants. Purified monomeric TDP‐43‐MBP‐His_6_ was collected by pooling the fractions corresponding to peak B in the SEC profile (Appendix Fig S1D). All purified proteins were concentrated using Amicon ultra centrifugal filters and then flash frozen and stored at −80°C. To determine protein concentration, absorbance at 280 nm was measured using the respective extinction coefficient (ε) predicted by the ProtParam tool. Additionally, for all purified proteins, the A260/280 ratio was determined and found to be between 0.5 and 0.7.

#### CK1δ

The kinase domain of CSNK1D was expressed as an N‐terminal MBP‐tagged fusion in *E. coli* Rosetta 2 cells, co‐expressing λ‐phosphatase to guarantee a completely unphosphorylated protein. The cells were grown to an OD of 0.45 and subsequently the temperature was reduced to 18°C. Then the cells were induced (generally at OD 0.7–0.8) with 0.5 mM IPTG and expression was performed overnight. Cells were harvested and resuspended in AC‐A buffer (25 mM Bis‐Tris, 500 mM NaCl, 10 mM β‐mercaptoethanol, pH 7.0), supplemented with DNAse, RNAse, lysozyme and protease inhibitor cocktail (selfmade) for cell disruption. Lysis was done by sonication on ice (5 × 30 s with breaks of 1 min between each pulse). Cell debris was pelleted by centrifugation (SS34 rotor, 34,541 *g*, 30 min). The supernatant was filtered and subsequently loaded on a Dextrin Sepharose column (cytiva), previously equilibrated with AC‐A buffer. The column was washed for 5 column volumes with AC‐A buffer. Elution was done with MBP‐B buffer (25 mM Bis‐Tris, 500 mM NaCl, 10 mM β‐mercaptoethanol, 20 mM maltose, pH 7.0). The eluted protein was subject to TEV protease cleavage overnight at 4°C. On the next day the buffer was exchanged to IEX‐A buffer (25 mM Bis‐Tris, 50 mM NaCl, 10 mM. β‐mercaptoethanol) by ultra‐filtration (Amicon Ultra‐15 30 kDa, Merck Millipore) and subject to cation‐exchange chromatography by a linear to IEX‐B buffer (25 mM Bis‐Tris, 500 mM NaCl, 10 mM. β‐mercaptoethanol). Eluted protein was concentrated and gel‐filtered over a Superdex 75 (cytiva) into SEC buffer (25 mM Bis‐Tris, 50 mM NaCl, 10 mM MgCl_2_, 1 mM DTT). Fractions were collected, concentrated and aliquots of 200 µl were flash frozen and stored at −80°C until use.

#### His_6_‐TEV protease

His_6_‐TEV protease expression and purification was performed as described in Hutten *et al* (2020).

### *In vitro* phosphorylation

TDP‐43‐MBP‐His_6_ was *in vitro* phosphorylated with CK1δ and 200 µM ATP in phosphorylation buffer (50 mM Tris–HCl, pH 7.5, 10 mM MgCl2, 1 mM DTT) for 2 h at RT, using a two‐fold molar excess of TDP‐43‐MBP‐His_6_ over CK1δ. Subsequently, the reaction was used for sedimentation and SG association assays. As negative controls, either the kinase or the ATP was omitted and also included as controls in subsequent assays.

### Enzymatic digestion, enrichment for phospho‐peptides and mass spectrometric analysis

TDP‐43‐MBP‐His_6_ was *in vitro* phosphorylated as described above, separated on a 10% SDS–PAGE gel and visualized by Coomassie staining. The gel band corresponding to the phosphorylated TDP‐43‐MBP‐His_6_ was excised and destained twice for 30 min at 37°C with 50% acetonitrile in 50 mM Tris–HCl, pH 8. The gel piece was dehydrated with 100% acetonitrile, reduced and alkylated, and finally digested overnight at 37°C with 375 ng trypsin (Promega). The peptides were extracted from the gel twice using 100 µl of 50% acetonitrile and 0.25% TFA buffer. Both extractions were merged and evaporated in a vacuum evaporator. In order to enrich the phospho‐peptides, 10 µl of 0.5 mg/µl TiO_2_ beads (GL Sciences Cat. No.: 5010‐21315) in loading buffer (80% ACN, 5% TFA and 1 M glycolic acid) were added to the dried samples in a ratio of 0.3 mg of beads to 5 pmol of protein. Samples were incubated for 10 min at RT on a shaker at 270 *g* and spun down at 100 *g* for 1 min. The supernatant was removed and kept for further analysis, while beads were sequentially washed with loading buffer, washing buffer 1 (80% ACN, 1% TFA) and washing buffer 2 (10% ACN, 0.2% TFA). Next, the beads were dried in the hood for 10 min and resuspended with 50 µl elution buffer (28% ammonia solution in H_2_O). Finally, the samples were speed vacuum evaporated and resuspended with 15 µl 0.1% FA. For LC‐MS purposes, desalted peptides were injected in an Ultimate 3000 RSLCnano system (Thermo) and separated in a 25‐cm analytical column (75 µm ID, 1.6 µm C18, IonOpticks) with a 30‐min gradient from 3 to 30% acetonitrile in 0.1% formic acid. The effluent from the HPLC was directly electrosprayed into a Qexactive HF (Thermo) operated in data‐dependent mode to automatically switch between full scan MS and MS/MS acquisition. Survey full scan MS spectra (from *m*/*z* 300–1,600) were acquired with resolution *R* = 60,000 at *m*/*z* 400 (AGC target of 3 × 10^6^). The 10 most intense peptide ions with charge states between 2 and 5 were sequentially isolated to a target value of 1 × 10^5^ with resolution *R* = 15,000 and isolation window 1.6 Th and fragmented at 27% normalized collision energy. Typical mass spectrometric conditions were: spray voltage, 1.5 kV; no sheath and auxiliary gas flow; heated capillary temperature, 250°C; ion selection threshold, 33.000 counts.

### Fluorescent labeling of purified TDP‐43

TDP‐43‐MBP‐His_6_ variants were labeled with Alexa Fluor 488 C5 maleimide (Thermo Fisher) at a low (~0.01–0.05) labeling efficiency in order to avoid interference with condensate formation. Labeling was performed according to the manufacture's protocol using a 1:100 or 1:20 protein:fluorescent dye mole ratio. Briefly, the Alexa Fluor reagent, previously dissolved in DMSO, was mixed with the protein and kept in the dark for 2 h at RT. Excess dye was removed by consecutive washes with TDP‐43 purification buffer using Amicon ultra centrifugal filters. Subsequently, labeled protein was used for spinning disc confocal microscopy, FRAP and aggregation assays, respectively.

### *In vitro* phase separation and aggregation assays

#### Sedimentation assay

For sedimentation analysis, 1 µM TDP‐43‐TEV‐MBP‐His_6_ variants or *in vitro* phosphorylated TDP‐43‐TEV‐MBP‐His_6_ was cleaved by addition of 20 µg/ml His_6_‐TEV protease in 50 or 25 µl Hepes buffer (20 mM Hepes, pH 7.5, 150 mM NaCl, 1 mM DTT), respectively, to remove the MBP‐His_6_ tag and induce phase separation. Samples were incubated for 60 min at 30°C, followed by centrifugation for 15 min at 21,000 g at 4°C to pellet the formed condensates. Equal amounts of supernatant (S) and condensate (C) fractions were loaded onto an SDS–PAGE gel and TDP‐43 was detected by Western Blot (rabbit TDP‐43 N‐term, Proteintech, Cat. No.: 10782‐2‐AP).

#### Microscopic condensate assay

For all microscopic condensate assays, uncoated µ‐Slide 18 Well‐Flat chambers (Cat. No.: 81821, Ibidi) were pretreated with 10% Pluronics F‐127 solution for 1 h and 5 times washed with MilliQ water. The water remained in the chamber until just before the experiment, as described in Ceballos *et al* (2018).

Purified TDP‐43‐TEV‐MBP‐His_6_ variants were buffer exchange to Hepes buffer or phosphate buffer (20 mM Na_2_HPO_4_/NaH_2_PO_4_, pH 7.5, 150 mM NaCl, 2.5% glycerol, 1 mM DTT). Proteins were then centrifuged at 21,000 *g* for 10 min at 4°C to remove any preformed protein precipitates. For condensates formation, the reaction was setup directly in Pluronics‐coated µ‐Slide 18 Well‐Flat chambers, where proteins were diluted to the indicated concentrations and phase separation was induced by addition of 100 µg/ml His_6_‐TEV protease at RT. After ~20 min, imaging was performed by bright field microscopy using a widefield microscope.

For fusion events and FRAP analysis, condensates were formed directly in Pluronics‐coated µ‐Slide 18 Well ‐ Flat chambers as described above using 20 µM of each Al.488‐labeled TDP‐43 protein variants (Wt, 5D and 12D) in Hepes buffer and incubated for 10 min at RT before imaging. Note that experiments were performed until maximally 1 h after adding the TEV protease, in order to avoid *in vitro* aging of condensates.

#### Turbidity assay

Phase separation of TDP‐43‐TEV‐MBP‐His_6_ variants was induced as described earlier for the microscopic condensate assay. Reactions of 20 µl samples were prepared at the indicated concentrations in 384‐well plates and incubated for 30 min at RT after adding TEV protease. Subsequently, a BioTek Power Wave HT plate reader was used to measure turbidity at 600 nm. Turbidity measurements were performed in triplicates.

#### Semi‐denaturing detergent agarose gel electrophoresis

SDD‐AGE experiments were performed based on protocols published by French *et al* (2019) and Halfmann and Lindquist (2008). First, 2 µM purified TDP‐43‐MBP‐His_6_ variants (WT, 5D, 12D and 12A) were set up in low binding tubes (Eppendorf) in 35 µl aggregation buffer (50 mM Tris pH 8.0, 250 mM NaCl, 5% glycerol, 5% sucrose, 150 mM imidazole pH 8.0) and supplemented with 1× protease inhibitor (Sigma). Samples were shaken for 30 min on a thermomixer at 1,000 rpm at RT (~22°C) and then incubated at RT for the indicated time period. 5 µl of each sample was collected and diluted in SDD‐AGE buffer (40 mM Tris–HCl pH 6.8, 5% glycerol, 0.5% SDS, 0.1% bromphenol‐blue) and analyzed by SDD‐AGE by horizontal 1.5% agarose gel electrophoresis (gel: 1.5% agarose in 20 mM Tris, 200 mM glycine and 0.1% SDS) in running buffer (60 mM Tris, 20 mM acetate, 200 mM glycine, 1 mM EDTA and 0.1% SDS) for ~6 h at 60 V. Detection of TDP‐43 monomers, oligomers and high‐molecular‐weight species was performed after overnight capillary transfer in TBS (50 mM Tris pH 7.6, 150 mM NaCl) to a nitrocellulose membrane and by standard Western Blot using rabbit anti TDP‐43 N‐term antibody (Proteintech, Cat. No.: 10782‐2‐AP).

### Formation of Alexa 488‐labeled TDP‐43 aggregates

In order to visualize TDP‐43 (wt, 5D, 12D and 12A) aggregates formed under the above described assay conditions, 10 µM Al.488‐labeled TDP‐43‐MBP‐His_6_ was set up in low binding tubes (Eppendorf) in aggregation buffer and incubated with or without 100 µg/ml His_6_‐TEV protease. Samples were shaken at 1,000 rpm at RT for 30 min and then transferred into a 384‐well black plate (Greiner Bio‐One), incubated at RT and imaged by confocal microscopy after 2, 8 and 24 h.

### Cellular TDP‐43 solubility assays

#### Fractionation in RIPA‐Benzonase buffer

HeLa cells (~1 × 10^6^) were washed twice in PBS, harvested by scraping and pelleted at 1,100 *g* for 5 min. Cell pellets were incubated on ice for 15 min in 200 µl RIPA buffer (50 mM Tris–HCl, pH 8.0, 150 mM NaCl, 1% NP‐40, 0.5% deoxycholate, 0.1% SDS) with freshly added 1× protease inhibitor cocktail (Sigma), 1× phosphatase inhibitors (final concentration: 10 mM NaF, 1 mM β‐glycerophosphate, 1 mM Na_3_VO_4_) and 0.05 unit/µl Benzonase (Sigma). Samples were sonicated in a BioRuptorPico (Diagenode) for 45 sec and 20 µl of sample was collected as “Input”. The remaining sample was then centrifuged at 13,000 *g* for 30 min at 4°C. The resulting supernatants (S) were collected and the remaining pellets were washed in RIPA buffer with inhibitors, resonicated for 45 sec and recentrifuged for 30 min at 4°C at 13,000 *g*. Finally, the RIPA insoluble pellets (I) were resuspended in 36 µl urea buffer (7 M urea, 2 M thiourea, 4% CHAPS, 30 mM Tris–HCl, pH 8.5) and sonicated. All samples were supplemented with 4× Lämmli buffer (250 mM Tris–HCl, pH 6,8, 40% glycerol, 8% SDS, 0.1% bromphenol‐blue, 4% β‐mercaptoethanol) and input and supernatant (S) samples were boiled prior to SDS–PAGE and Western Blot against TDP‐43 (rabbit anti TDP‐43 C‐term, Proteintech, Cat. No.: 12892‐1‐AP) and Myc (mouse anti‐myc 9E10 antibody, Helmholtz Center Munich). Note that for detection reasons, the (I) fractions were 4× more concentrated than the (S) fractions, so they are represented in a 1:5 ratio.

#### Filter trap assay

Cortical neurons expressing the indicated EGFP‐tagged TDP‐43 variants (DIV5 + 4 days expression) were washed two times with PBS and lysed on ice in RIPA buffer (50 mM Tris–HCl, pH 8.0, 150 mM NaCl, 1% NP‐40, 0.5% deoxycholate, 0.1% SDS) freshly supplemented with 1× protease inhibitor cocktail (Sigma), 1× phosphatase inhibitor cocktail (Sigma) and 0.125 Units/µl benzonase (Sigma) for 20 min. Cell lysates were collected and centrifuged at 1,000 *g*, 4°C for 30 min. Two‐third of the resulting supernatant (RIPA‐insoluble fraction) was filtered through a nitrocellulose membrane (0.2 µM pore size, Merck) using a filter trap slot blot (Hoefer Scientific Instruments). After washing with PBS for three times, membranes were blocked for 1 h with 2% I‐Block (Thermo Fisher) prior to immuno‐detection with mouse anti‐GFP (UC Davis/NIH Neuromab Facility, Cat. No.: N86/8) and rabbit anti Calnexin antibody (Enzo Life Sciences, Cat. No.: ADI‐SPA‐860). The remaining 1/3 of the lysates was diluted with 3× loading buffer (200 mM Tris–HCl pH 6.8, 6% SDS, 20% glycerol, 0.1 g/ml DTT, 0.1 mg bromophenol blue), boiled at 95°C and used for subsequent standard Western Blot analysis.

### Multi‐scale MD simulations

#### Explicit solvent coarse‐grained MD simulations

Coarse‐grained MD simulations with explicit solvent to investigate protein phase separation and phase‐separated protein condensates were run with a rescaled version of the Martini forcefield (Marrink *et al*, 2007; Monticelli *et al*, 2008) as described by Benayad *et al* (2021). A similar approach was shown to describe the conformational ensembles of proteins with disordered domains very well (Larsen *et al*, 2020; Martin *et al*, 2021), and we recently showed that such approaches can be extended to simulations of LLPS of disordered proteins (Benayad *et al*, 2021). Protein–protein interactions were thus scaled to 0.8 of the default value. Chloride and sodium ions were added to neutralize the system in simulations of Wt and 12D proteins. 10% of the water beads were replaced by WF anti‐freeze beads. Coarse‐grained simulations were run with GROMACS 2018 (Abraham *et al*, 2015). Simulations boxes measured 450 × 450 × 600 Å. Simulations systems were energy minimized and equilibrated in MD simulations with and without position restraints. 118 Wt and 12D C‐terminal LCDs (aa. 261–414) were simulated for 20 µs each. The coarse‐grained simulations systems consist of roughly one million particles. Equations of motions were integrated with a 20‐fs time step. Simulations were conducted in the NPT ensemble at 1 bar and 300 K using the Parrinello‐Rahman barostat (Parrinello & Rahman, 1981) and the Bussi–Donadio–Parrinello velocity‐rescaling thermostat (Bussi *et al*, 2007).

Note that in the coarse‐grained approach we employed, four atoms are typically grouped together to a coarse‐grained particle. For example, a coarse‐grained water molecule would correspond to four water molecules in an atomistic simulation.

#### Implicit solvent coarse‐grained MD simulations

The HPS coarse‐grained model provides a coarser and thus computationally very efficient description of disordered proteins and their phase behavior (Dignon *et al*, 2018). Parameters for PTMs, such a phosphorylation, are available (Perdikari *et al*, 2021). Solvent is treated implicitly and electrostatics is described by Debye–Hückel theory. We simulated 100 C‐terminal LCDs (aa. 261–414) in slab geometry (212 Å × 212 Å × 2800 Å) following the protocol of Mittal and co‐workers (https://bitbucket.org/jeetain/hoomd_slab_builder/src/master/) (Dignon *et al*, 2018; Regy *et al*, 2020). Simulations were started with all proteins concentrated and equilibrated in a small sub volume so that the proteins formed an initial condensate. We studied the phase behavior of Wt, 12D, 12A, 5pS and 12pS TDP‐43 LCDs. Production simulations (*T* = 310 K) were run for at least 5.8 μs and up to 6.5 μs for each LCD variant.

#### Atomistic MD simulations

HCG (Pietrek *et al*, 2020) enables us to generate statistically independent and chemically‐meaningful conformations of a biomolecular condensate with atomic resolution, which serve as starting points for atomistic MD simulations. Atomic‐resolution models of clusters of the C‐terminal disordered domain of TDP‐43 (aa. 261–414) were generated for both Wt protein and the 12D mutant. To assemble the disordered proteins into a condensate, we first assemble pairs of disordered domains, then pairs of pairs, pairs of quadruplets, and so forth, following the logic set out in (Pietrek *et al*, 2020). HCG Monte Carlo manifestly satisfies detailed balance and thus we generate representative ensembles. Finally, we arrive at densely packed disordered domains, while retaining atomic resolution at each modeling step. Periodic boundary conditions were employed during the assembly.

Clusters of Wt and 12D LCDs were solvated in a 150 Å × 150 Å × 150 Å simulation box, the system charge was neutralized and 150 mM NaCl was added. We employed the Amber‐disp protein force field developed by Robustelli *et al* (2018), including the modified TPIP4P‐D water model (Piana *et al*, 2015) that accompanies the Amber‐disp protein force field. Temperature was maintained at 300 K by the Bussi–Donadio–Parrinello velocity‐rescaling thermostat (Bussi *et al*, 2007). We employed the Parrinello–Rahman barostat (Robustelli *et al*, 2018) to set the pressure to 1 bar. Equations of motions were integrated with a 2‐fs time step. Production simulations were prepared by energy minimization with and without soft‐core potentials. To start production simulations, we equilibrated the atomistic simulations systems, running at least 5,000 steps with a 1‐fs time step and position restraints and for 1.5 ns with a 2‐fs time step also with position restraints. Equilibrium simulations of the clusters of the disordered domains were conducted with GROMACS 2019 (Abraham *et al*, 2015). For both wild‐type and 12D, clusters of 32 chains with 154 residues were simulated for just over 1 µs, with two repeats each started from independently generated HCG structures.

Simulations were analyzed with the MDAnalysis (Michaud‐Agrawal *et al*, 2011; Gowers *et al*, 2016) and the MDtraj Python libraries (McGibbon *et al*, 2015). Contact analysis was performed with the Contact Map Explorer Python library (https://github.com/dwhswenson/contact_map).

#### Analysis of MD simulations

Simulations were analyzed with the MDAnalysis (Michaud‐Agrawal *et al*, 2011; Gowers *et al*, 2016) and the MDtraj Python libraries (McGibbon *et al*, 2015). Contact analysis of the coarse‐grained simulations with the explicit solvent Martini model was performed with the Contact Map Explorer Python library (https://github.com/dwhswenson/contact_map). For simulations with the HPS implicit solvent coarse‐grained model, contacts were computed with MDAnalysis. Two amino acids were deemed in contact in the simulations with the HPS model when their inter‐bead distance was < 2^1/6^σ*_ij_* where σ*_ij_* = ½(σ*_i_* + σ*_j_*) is the average of bead diameter of the respective amino acids *i* and *j*. The concentrations *c*_dilute_ and *c*_dense_ of dilute and dense phases, respectively, were determined by adapting the workflow of Tesei *et al* (https://github.com/KULL‐Centre/papers/tree/main/2021/CG‐IDPs‐Tesei‐et‐al) from the simulations of the HPS model (preprint: Tesei *et al*, 2021). The excess free energy of transfer from the dilute to the dense phase was then computed as Δ*G*_trans_ = −*RT* ln (*c*_dense_/*c*_dilute_), where *R* is the gas constant and *T* is the absolute temperature.

### Nuclear transport assay

To analyze import of GCR_2_‐EGFP_2_ tagged TDP‐43 reporters, HeLa cells were grown for at least two passages in DMEM supplemented with 10% dialyzed FBS and were transiently transfected with the different GCR_2_‐EGFP_2_‐TDP‐43 variants as described earlier. Import of the GCR_2_‐EGFP_2_‐TDP‐43 reporters was induced by adding dexamethasone (5 µM final concentration) in imaging medium (fluorobrite) and followed by live cell imaging using a spinning disk confocal microscope (see below).

### SG association assay

HeLa cells were grown on high precision (No. 1.5) poly‐l‐lysine coated 12 mm coverslips, and after SG induction with MG132 (10 µM for 2.5–3 h), cells were permeabilized for 2× 2 min with 0.004–0.005% digitonin in KPB (20 mM potassium phosphate pH 7.4, 5 mM Mg(OAc)_2_, 200 mM KOAc, 1 mM EGTA, 2 mM DTT and 1 mg/ml each aprotinin, pepstatin and leupeptin). Soluble proteins were removed by several, stringent washes (4× 4 min in KPB on ice) before blocking nuclear pores by 15 min incubation with 200 mg/ml wheat germ agglutinin (WGA) on ice. Cells were then incubated for 30 min at RT with 100 nM TDP‐43‐MBP‐His_6_. For comparison of *in vitro* phosphorylated TDP‐43 with controls, proteins were either subjected to the *in vitro* phosphorylation reaction or mock treated (Wt, 12D) in absence of kinase or ATP before exchanging the buffer to KPB using 40K Zeba spin desalting columns (Thermo). Subsequently, cells were washed (3× 5 min in KPB on ice) to remove unbound TDP‐43‐MBP‐His_6_ and processed by immunostaining to visualize SGs. SGs and TDP‐43‐MBP‐His_6_ were visualized by G3BP1 immunostaining (rabbit anti G3BP1antibody, Proteintech, Cat. No.: 13057‐2‐AP) and MBP immunostaining (by mouse anti MBP antibody, Proteintech, Cat. No.: 66003‐1‐Ig), respectively. On Fig 4B for clarity, signals were converted to grey values in the individual channels (upper two rows). In the merge (lower row), G3BP1 is shown in magenta, TDP‐43‐MBP‐His_6_ in green, white pixels indicate colocalization. Nuclei were counterstained with DAPI (turquoise).

### siRNA‐mediated knockdown of TDP‐43

TDP‐43 knockdown was achieved using the pre‐designed TARDBPHSS118765 siRNA (Invitrogen) as described in Dormann *et al* (2009). Briefly, 20 nM siRNA was transfected into HeLa or U2OS cells using RNAimax (Thermo) transfection reagent. Knockdown was analyzed 48 h post transfection by immunohistochemistry using mouse anti TDP‐43 antibody (Proteintech, Cat. No.: 60019‐2‐Ig) and immunoblotting using rabbit anti TDP‐43 C‐Term antibody (Proteintech, Cat. No.: 12892‐1‐AP) to detect TDP‐43 and mouse anti alpha‐Tubulin antibody (Proteintech, Cat. No.: 66031‐1‐Ig) for detection of α‐Tubulin as a control.

### RNA extraction and RT–PCR to analyze TDP‐43 splice targets

TDP‐43 expression was silenced in HeLa cells by siRNA as described earlier and 24 h later cells were transfected with siRNA‐resistant pcDNA5‐FRT‐TO‐Myc‐hTDP43 constructs (Wt, 12D and 12A). 48 h after transfection, cells were harvested and total RNA was extracted using an RNeasy mini kit from Qiagen. cDNA was synthesized using 500 ng of total RNA, M‐MLV reverse transcriptase polymerase (Invitrogen), and 6 µM of random hexamer primer (NEB). cDNA was amplified with Taq DNA polymerase (NEB) using the forward (FW) and reverse (RV) primers targeting the *SKAR* gene (FW—5′CCTTCATAAACCCACCCATTGGGACAG3′; RV—5′GTGGTGGAGAAAGCCGCCTGAG3′) (Fiesel *et al*, 2012) and the *BIM* gene (FW—5′TCTGAGTGTGACCGAGAAGG3′; RV—5′TCTTGGGCGATCCATATCTC 3′) (Tollervey *et al*, 2011). PCR products were separated by electrophoresis on a 2.5% agarose gel containing GelRed (Sigma).

### Electrophoretic mobility shift assays

The TDP‐43 autoregulatory RNA site (Ayala *et al*, 2011) located in the *TARDBP* 3′UTR (5′UCACAGGCCGCGUCUUUGACGGUGGGUGUCCCAUUUUUAUCCGCUACUCUUUAUUUCAUGGAGUCGUAUCAACGCUAUGAACGCAAGGCUGUGAUAUGGAACCAGAAGGCUGUCUGAACUUUUGAAACCUUGUGUGGGAUUGAUGGUGGUGCCGAGGCAUGAAAGGCUAGUAUGAGCGAGAAAAGGAGAGAGCGCGUGCAGAGACUUGGUGGUGCAUAAUGGAUAUUUUUUAACUUGGCGAGAUGUGUCUCUCAAUCCUGUGGCUUUGGUGAGAGAGUGUGCAGAGAGCAAUGAUAGCAAAUAAUGUACGAAUGUUUUUUGCAUUCAAAGGACAUCCACAUCUGUUGGAAGACUUUUAAGUGAGUUUUUGUUCUUAGAUAACCCACAUUAGAUGAAUGUGUUAAGUGAAAUGAUACUUGUACUCCCCCUACCCCUUUGUCAACUGCUGUG) was *in vitro* transcribed from double‐stranded DNA templates and Cy5‐labeled using the HyperScribe™ T7 High Yield Cy5 RNA Labeling Kit (APExBIO, Cat. No.: K1062) per manufacturer's instructions. (UG)_12_ RNA (5′ UGUGUGUGUGUGUGUGUGUGUGUG) was chemically synthesized with the addition of a 5′ Cy5‐label (Metabion). 16 nM of Cy5‐labeled RNA was mixed with varying amounts of TDP‐43 Wt, 12A, and 12D (0–1.6 µM). Binding reactions (20 µl) were incubated in binding buffer (20 mM NaPO_4_ [pH 8], 150 mM NaCl, 1 mM DTT, 5 mM MgCl_2_, 0.5 mg/ml BSA, 0.1 mg/ml yeast tRNA, 5% glycerol and 1 U/µl RNase inhibitor [NEB]) for 20 min at RT before loading onto a 1‐mm thick non‐denaturing polyacrylamide gel (6%) in 0.5× TBE. Gels were run at 100 V for 1 h at RT. Gels were imaged with a Typhoon™ FLA 9500 laser scanner.

### Immunostaining

All steps were performed at RT. HeLa and Flp‐In T‐Rex U2OS cells were fixed in 4% formaldehyde in PBS for 10 min, permeabilized for 5 min using 0.2% (v/v) Triton X‐100 in PBS and subsequently blocked in blocking buffer (5% goat or donkey serum in 0.1% saponine in PBS) for 30 min. Primary and secondary antibodies were diluted in blocking buffer and applied each for 1 h and washed three times using 0.1% saponine in PBS. Myc‐TDP‐43 was stained using mouse anti TDP‐43 antibody (Proteintech) or mouse anti‐myc 9E10 antibody (IMB protein production core facility), SGs were stained using goat anti TIA1 antibody (Santa Cruz, Cat. No.: sc‐48371) or rabbit anti G3BP1 antibody (Proteintech) and DNA was stained with DAPI at 0.5 μg/ml in PBS for 5 min. Coverslips were then mounted on glass slides with ProLong™ Diamond Antifade reagent (Life Technologies) and dried overnight at RT.

Hippocampal neurons cultured on glass coverslips were washed twice with PBS and fixed for 10 min at RT using 4% paraformaldehyde and 4% sucrose in PBS. Primary antibody as well as secondary antibody (1:400) were diluted in GDB buffer (0.1% gelatin, 0.3% Triton X‐100, 450 mM NaCl, 16 mM sodium phosphate pH 7.4). Primary antibodies (Mouse anti Map2, Sigma, Cat. No.: M1406; Rabbit anti G3BP1, Abam, Cat. No.: ab181150) were incubated overnight at 4°C while secondary antibodies was applied for 1 h at RT, each followed by three times washing with PBS. Coverslips were mounted using Vectashield Vibrance with DAPI (Biozol) to counterstain nuclei.

### Microscopy

#### Bright and wide‐field microscopy

Imaging of unlabeled TDP‐43 condensates was done by bright‐field microscopy on an Axio Oberver.Z1 wide‐field fluorescence microscope, using a 63×/1.40 Oil objective and an AxioCam 506 (Zeiss, Oberkochen, Germany).

#### Confocal microscopy

Confocal microscopy of TDP‐43 aggregates and HeLa cells was performed using an inverted Leica SP8 microscope and the LAS X imaging program (Leica), provided by the Bioimaging core facility of the Biomedical Center (LMU Munich), which included the excitation lasers for 405, 488, 552 and 638 nm. Images were acquired using two‐fold frame averaging with a 63×1.4 oil objective, with an image pixel size of 180 nm for Al.488‐TDP‐43 aggregates and fixed cells, and 59 nm for images of cells subjected to the SG association assay. Confocal images of U2OS cells were obtained using an inverted Leica SP5 microscope and the LASAF imaging program (Leica), provided by the Light Microscopy core facility of the Biocenter (JGU Mainz). Images were acquired using two‐fold frame averaging with a 100× 1.4 oil objective, with an image pixel size of 151 nm.

The following fluorescence settings were used for detection: DAPI: 419–442 nm, GFP: 498–533 nm, Alexa 555: 562–598 nm and Alexa 647: 650–700 nm. Recording was performed sequentially using a conventional photomultiplier tube to avoid bleed‐through.

#### Spinning disc confocal microscopy

- Nuclear transport assay imaging:
  Images were acquired for a duration of 50 min in 2.5 min intervals at 36.5°C and 5% CO_2_ (EMBLEM environmental chamber) using an inverted microscope (Axio Observer.Z1; Carl Zeiss, Oberkochen, Germany) equipped with a confocal spinning disc (CSU‐X1; Yokogawa, Tokyo, Japan) and a 63×/1.4 oil immersion lens. Images were acquired using the 488 nm SD laser line and an EM‐CCD camera (EvolveDelta; Photomoetrics) at bin 1 × 1.
- Fusion events and FRAP:
  Experiments were performed on an inverted microscope (Axio Observer.Z1; Carl Zeiss, Oberkochen, Germany) equipped with a confocal spinning disk unit (CSU‐X1; Yokogawa, Tokyo, Japan) and an oil immersion lens of 100×/1.46 Oil Ph3. Images recording the dynamics of TDP‐43 condensates were obtained using a EM‐CCD camera (EvolveDelta; Photomoetrics), with a bin 1 × 1 in a recording mode of 5 s intervals in a block of 3 min. Images of TDP‐43 condensates after bleaching were acquired with bin 1 × 1 in streaming mode for 1.5 s followed by a block of 2 min where images were recorded in intervals of 5 sec. Experiments were performed at RT and ≥ 11 condensates were analyzed per condition in three independent experiments. Localized photobleaching (“half‐bleach”) was obtained using a laser scanning device (UGA‐42 Geo; Rapp OptoElectronic, Hamburg, Germany). The “Geo” module allowed for simultaneous laser illumination within hardware‐defined shapes of different sizes. For this experiment, an illumination size of ~4 µm in a square‐like shape was used. The targeting structure was half bleached to approximately 70% of the initial intensity using a 473‐nm diode laser (DL‐473/75; Rapp OptoElectronic, Hamburg, Germany).

### Quantification and analysis

#### Droplet quantification

Wide‐field images of droplets were processed and quantified and measured using Image J/Fiji software. First, a bandpass filter of 20 pixels was applied to all images in order to reveal some details and thresholds were adjusted to optimally include all droplets. Finally, droplets were counted and measured by their size and roundness [4*area/(π*major_axis^2^), or the inverse of the aspect ratio] using the command Analyze Particles, excluding the detection of particles with a circularity below 0.3 and/or an area smaller than 3 pixels. Statistical analyses were performed using GraphPad Prism 8.

#### Analysis of cellular images

Analysis of the nuclear transport assay was performed using Image J/Fiji software by measuring loss in cytoplasmic fluorescence over time and normalizing *t* = 0 min to 1.

Images of cells from the SG association assay (Hutten & Dormann, 2020) were processed and analyzed using Image J/Fiji software, applying linear enhancement for brightness and contrast and implemented plugins for measurement of pixel intensities in SGs.

Quantification of Myc‐hTDP‐43 recruitment into SGs was performed using Image J/Fiji software. First, SGs from TDP‐43‐positive cells were selected using the Wand tracing tool and a band of 1 µm representing a proxy for the cytosol was drawn around all selected SGs using the “Make Band” command. Then, all pixel intensities for both SG and band selections was extracted for the TDP‐43 channel. After subtraction of the background signal from all measured values, calculation of the SG/band ratio was performed for each SG.

Analysis of Myc‐hTDP‐43 recruitment into NBs was performed by counting the number of cells with positive TDP‐43 nuclear condensates, excluding cells expressing TDP‐43 staining only in the cytoplasm. Profile of TDP‐43 nuclear staining was performed using Image J/Fiji software by using the “Plot Profile” command, which quantifies the gray values along the indicated lines.

All statistical analyses were performed using GraphPad Prism 8.

#### WB analysis

WB analysis was performed by extracting the optical densities of each band using the software Image Studio Lite (LI‐COR), using the top and bottom average background option, to obtain the signal value, in which local background is automatically subtracted.

Analysis of Sedimentation assays and Fractionation in RIPA‐Benzonase buffer experiments was performed by dividing the signal values of (S) by the total (S + C) or (S + I) signal values, to obtain a S/(S + C) or S/(S + I) ratio, respectively.

Analysis of TDP‐43 autoregulation levels in the Flp‐In T‐Rex U2OS cell line was performed by comparing the signal values of endogenous TDP‐43 protein between induced (+Dox) and non‐induced (−Dox) expression conditions of myc‐hTDP‐43 variants. After housekeeping protein normalization using Histone H3, endogenous TDP‐43 protein expression levels were normalized to 1 in the myc‐TDP‐43 Wt (−Dox) condition.

All statistical analyses were performed using GraphPad Prism 8.

#### FRAP analysis

FRAP analysis were performed using Image J/Fiji software by calculating the fluorescence intensity over time (*I*(*t*)) using the macro Time Series Analyzer command and the following formula:

$$I\left(t\right)=\frac{\left[\text{ROI}1\left(t\right)‐\text{ROI}3\left(t\right)\right]}{\left[\text{ROI}2\left(t\right)‐\text{ROI}3\left(t\right)\right]}.$$

ROI1 corresponds to the averaged gray values of the bleached area, and ROI2 to the averaged gray values of the total droplet. ROI3 corresponds to the averaged background values. Values were further normalized to the initial fluorescence by dividing *I*(*t*) by the mean gray value of the initial 1 time step before bleaching <*I*(1)>. This way bleached areas were corrected for background noise and bleaching due to imaging over time. Statistical analyses were performed using GraphPad Prism 8.

## Author contributions

**Lara A Gruijs da Silva:** Investigation; Visualization; Methodology; Writing—original draft; Writing—review and editing. **Francesca Simonetti:** Investigation; Visualization; Methodology; Writing—review and editing. **Saskia Hutten:** Investigation; Visualization; Methodology; Writing—review and editing. **Henrick Riemenschneider:** Investigation; Visualization; Methodology; Writing—review and editing. **Erin L Sternburg:** Investigation; Visualization; Methodology; Writing—review and editing. **Lisa M Pietrek:** Methodology. **Jakob Gebel:** Resources; Investigation; Methodology. **Volker Dötsch:** Resources. **Dieter Edbauer:** Resources; Supervision; Methodology; Writing—review and editing. **Gerhard Hummer:** Resources; Methodology; Writing—review and editing. **Lukas D Stelzl:** Investigation; Visualization; Methodology; Writing—original draft; Writing—review and editing. **Dorothee Dormann:** Conceptualization; Supervision; Funding acquisition; Writing—original draft; Writing—review and editing.

In addition to the CRediT author contributions listed above, the contributions in detail are:

Conceptualization: DD, LAGS; Methodology: all authors; Investigation: LAGS, FS, SH, HR, ELS, LMP, JG, LSS; Resources: DD, GH, LSS, DE, VD; Writing–original draft: DD, LAGS; Writing–review and editing: all authors; Visualization: LAGS, FS, SH, HR, ELS, JG, LSS; Supervision: DD, GH, DE, VD, LSS; Project administration: DD; Funding acquisition: DD, DE, GH, LSS, VD.

## Supporting information



Appendix

Expanded View Figures PDF

Movie EV1

Movie EV2

Movie EV3

Movie EV4

Movie EV5

Source Data for Expanded View/Appendix

Source Data for Figure 1

Source Data for Figure 2

Source Data for Figure 4

Source Data for Figure 6

## Data Availability

This study includes no data deposited in external repositories.
