# Supplementary material for: Disease‐linked TDP‐43 hyperphosphorylation suppresses TDP‐43 condensation and aggregation
Source: EMBO J. 2022 Feb 3;41(8):e108443. doi: 10.15252/embj.2021108443 (PMC9016352; doi:10.15252/embj.2021108443)
Supplement: Supplementary file 1 — Appendix [file EMBJ-41-e108443-s002.pdf]

Appendix

Disease-linked TDP-43 hyperphosphorylation suppresses TDP-43 condensation and aggregation

Table of content

Appendix Figure S1. TDP-43-MBP-His_6 purification.

Appendix Figure S2. PLAAC predicts a reduced prion-like character of phosphomimetic TDP-43 12D.

Appendix Figure S3. SDD-AGE analysis of TDP-43-MBP-His_6 with and without TEV protease-mediated cleavage.

Appendix Figure S4. Differences in protein-protein and protein-water interactions in atomistic simulations of dense clusters of Wt and 12D TDP-43 LCDs.

Appendix Figure S5. Analysis of coarse-grained simulations with the implicit-solvent HPS model.

Appendix Figure S6. Controls showing endogenous TDP-43 levels after siRNA-mediated knockdown.

Appendix Figure S7. Control experiments for localization of TDP-43-NLS mutants.

Appendix Figure S8. Control experiments for localization of GFP-TDP-43 variants in primary cortical neurons.

Appendix Table S1. Concentrations of dense and dilute phases in simulations with the HPS coarse-grained model.

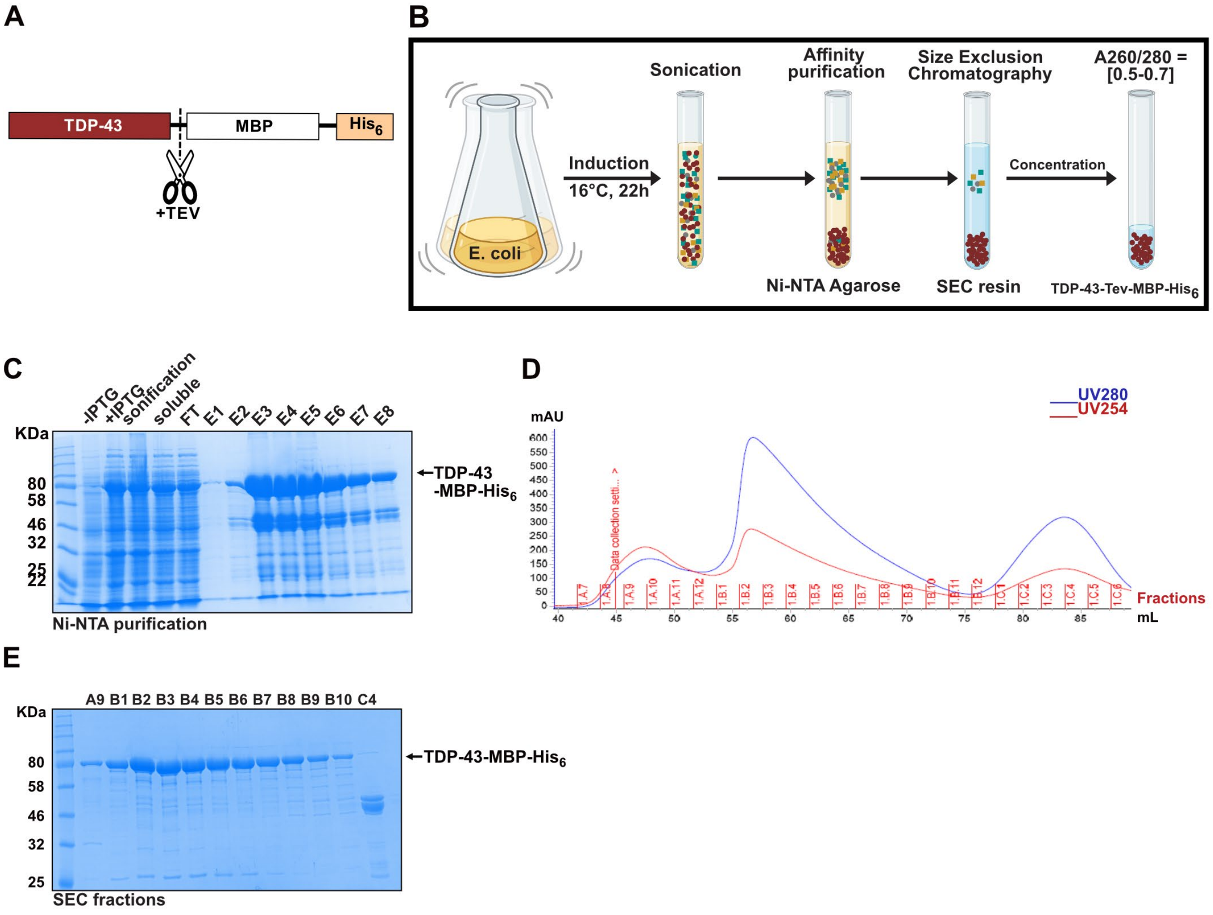

Appendix Figure S1. TDP-43-MBP-His$_6$ purification.

A Schematic representation of purified full-length TDP-43 containing a solubilizing maltose binding protein (MBP) tag, a His$_6$-tag and a TEV protease recognition site upstream of the MBP tag.

B Schematic diagram of TDP-43-MBP-His$_6$ expression and purification (created in BioRender.com). The protein is expressed in *E. coli* for 22 h at 16 °C and bacteria are lysed by sonication. Then the protein is purified under high salt conditions via Ni-NTA affinity purification and size exclusion chromatography (SEC) to obtain clean soluble TDP-43-MBP-His$_6$ (red circles), which is largely devoid of nucleic acids, as judged from the A260/280 ratio.

C Representative Coomassie-stained SDS-PAGE gel of the different steps from protein expression to Ni-NTA affinity purification. First and second lines correspond to samples before (-IPTG) and after (+IPTG) induction of TDP-43-MBP-His$_6$ expression. Third, fourth and fifth line correspond to samples after sonication, supernatant collection after spin down (soluble) and flow through (FT) after first wash with lysis buffer. The last 8 lines correspond to the consecutive elution steps (E1-E8) from the Ni-NTA agarose beads, fractions E2-E6 were pooled and used for SEC. ~89 kDa bands correspond to TDP-43-MBP-His$_6$.

D Representative SEC profile showing the absorption (mAU) of protein (UV280) and nucleic acids (UV254) on the y-axis, versus the volume (mL) of the different fractions on the x-axis.

E Coomassie-stained SDS-PAGE gel showing the indicated fractions (A9-C4) of the SEC run displayed in (D). A9, B1-B10 and C4 are fraction samples from peaks A, B and C in the SEC profile (D) and represent TDP-43-MBP-His$_6$ oligomeric, monomeric and cleaved species, respectively.

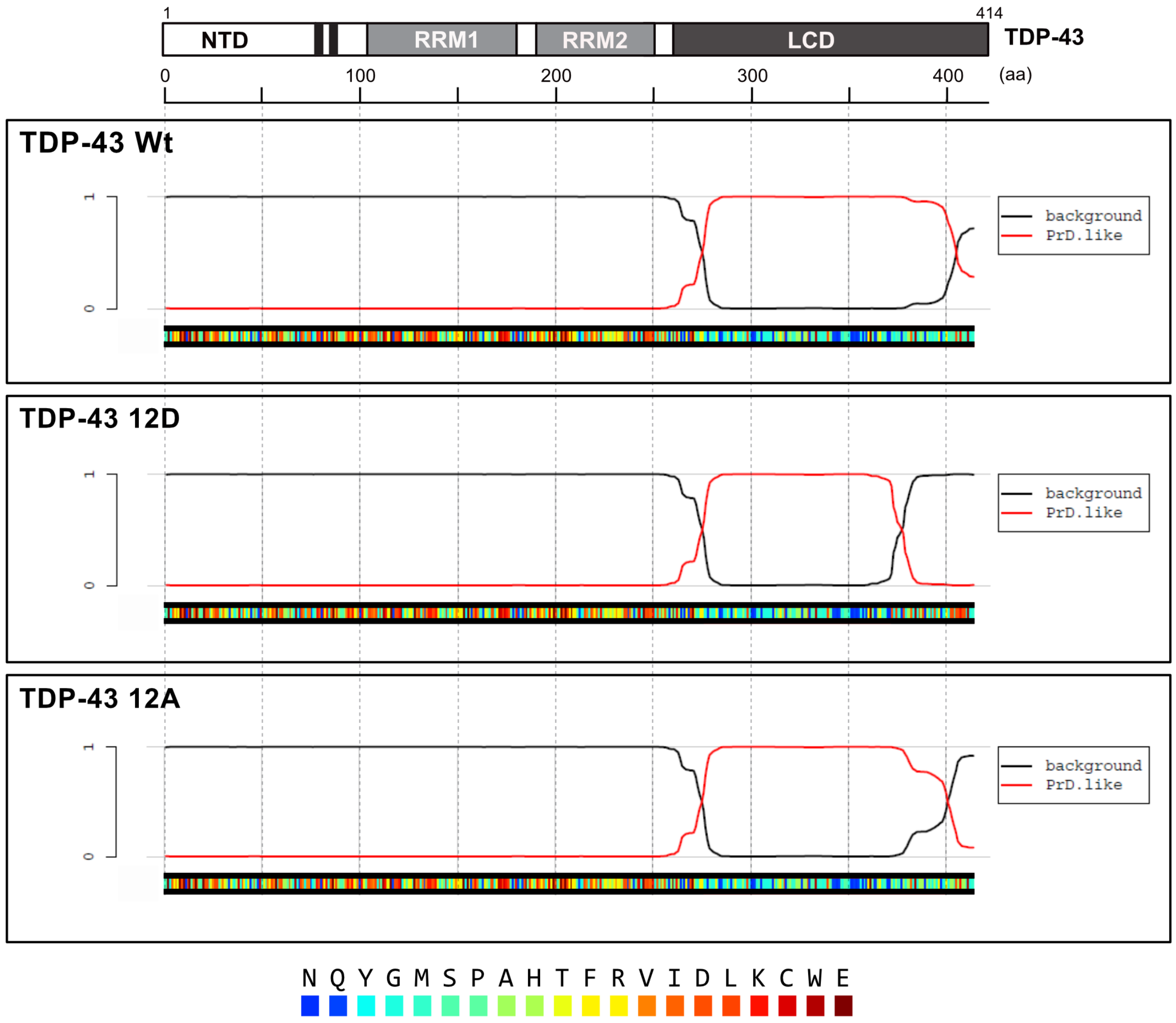

Appendix Figure S2. PLAAC predicts a reduced prion-like character of phosphomimetic TDP-43 12D.

Visualization outputs from PLAAC, a web application that scans protein sequences for domains with **p**rion-**l**ike **a**mino **a**cid **c**omposition (Lancaster et al., 2014). Each box shows a detailed visualization of different TDP-43 full-length variants (TDP-43 Wt, 12D and 12A) and respective Hidden Markov Model (HMM) prion-prediction score (probability scale from 0 to 1, shown on the y-axis), predicting prion domain-like (PrD. like, red) and non-prion domain-like (background, black) regions. The prediction reveals that the prion-like character of the C-terminal TDP-43 low complexity domain (LCD) is reduced by the 12 phosphomimetic S-to-D mutations. Bars below each diagram show amino acids (color-coded as in the legend).

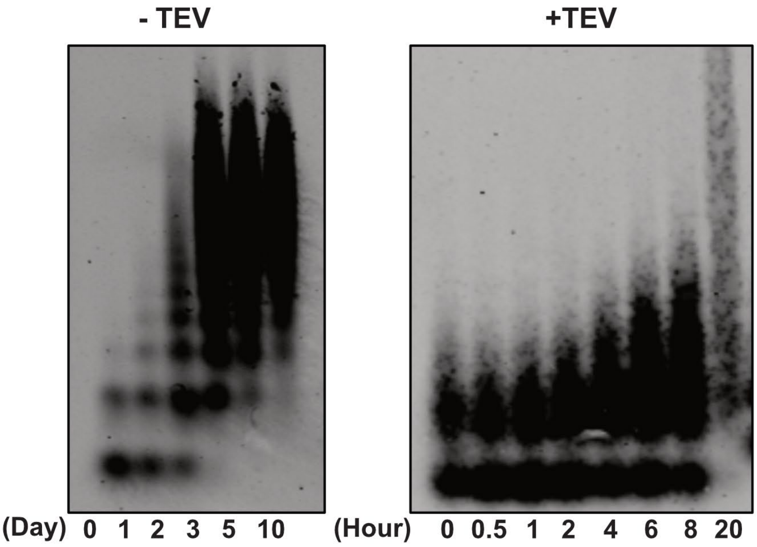

Appendix Figure S3. SDD-AGE analysis of TDP-43-MBP-His$_6$ with and without TEV protease-mediated cleavage.

Left: *In vitro* aggregation assay of TDP-43-MBP-His$_6$ in the absence of TEV protease (-TEV) leads to the appearance of SDS-resistant oligomers / high molecular weight species after several days of incubation. Distinct bands representing TDP-43-MBP-His$_2$ monomers, dimers, oligomers can be distinguished for the early timepoints (day 0 - 2). Note that the blot is the same as in Fig. 2E WT.

Right: *In vitro* aggregation assay of TDP-43-MBP-His$_6$ in the presence of TEV protease (+TEV), i.e. the MBP-His$_6$ tag is cleaved off with TEV protease. SDS-resistant oligomers / high molecular weight species already can be detected after several hours, but no distinct bands can be distinguished.

Detection was performed after SDD-AGE analysis by anti-TDP-43 Western blot (rabbit anti-TDP-43 N-term).

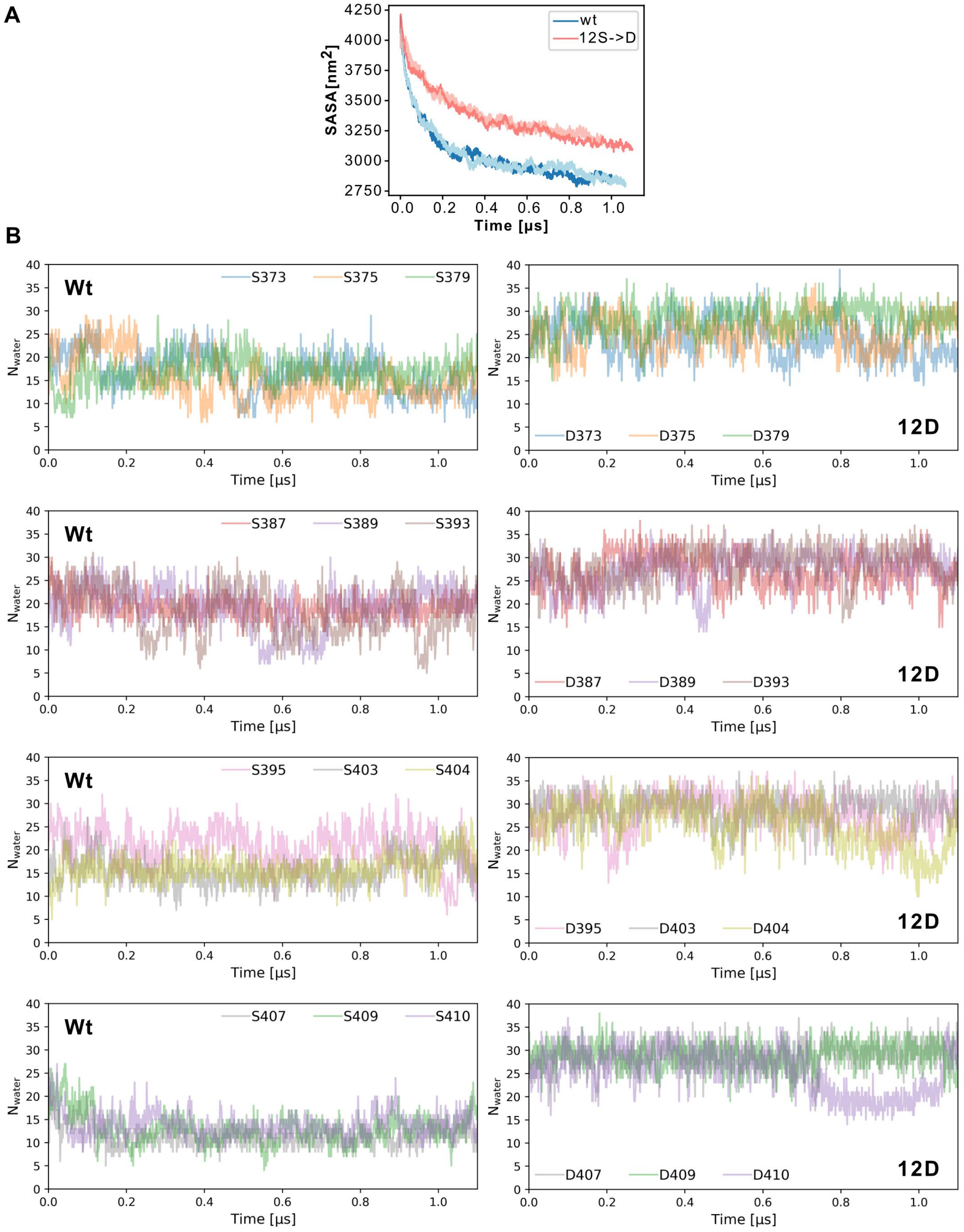

Appendix Figure S4. Differences in protein-protein and protein-water interactions in atomistic simulations of dense clusters of Wt and 12D TDP-43 LCDs.

A Solvent-accessible surface area (SASA) of the LCDs of Wt (blue and light blue) and 12D mutant (red and salmon) in atomistic simulations of dense protein solution. 12D mutant LCDs are more solvent-exposed than Wt LCDs, which is in line with the Wt LCDs forming stronger homotypic interactions.

B Dynamics of sidechain-water interactions at the sites of phosphomimicking S-to-D substitutions in atomistic simulations of Wt and 12D LCD clusters. Numbers of waters bound to sites of phosphomimicking S-to-D mutations (within 5 Å of the sidechain) are tracked over time for Wt (left) and 12D LCDs (right). While interactions are dynamic, there is a consistent trend that the phosphomimicking Asp residues bind more water molecules than the Ser residues in Wt LCDs.

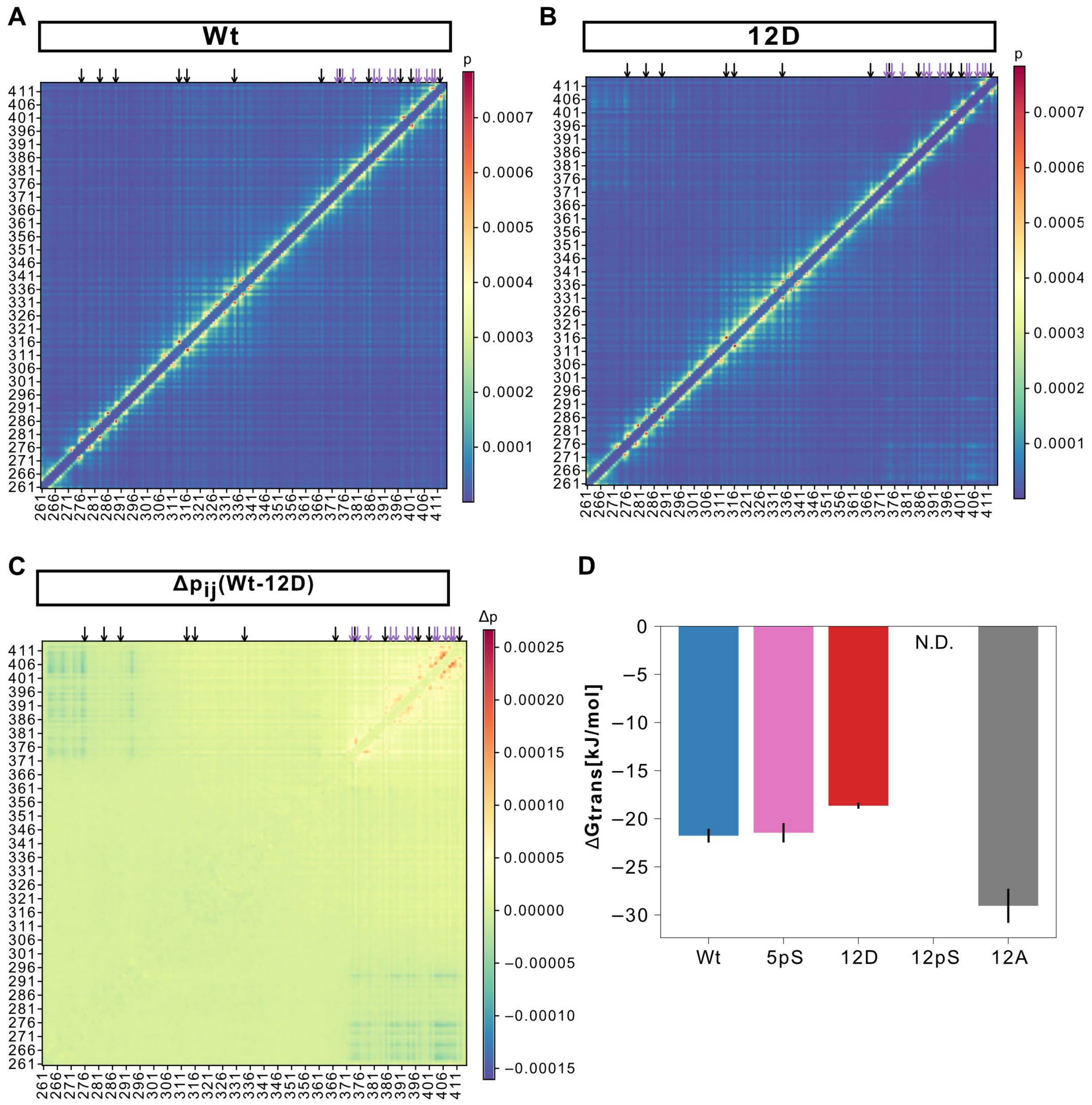

Appendix Figure S5. Analysis of coarse-grained simulations with the implicit-solvent HPS model.

A, B Contact maps for Wt (A) and 12D (B) TDP-43 LCD from simulations with the implicit solvent HPS coarse-grained model. Aromatic residues form prominent contacts and are highlighted by black arrows. The sites of the phosphomicking S-to-D mutations are highlighted by purple arrows.

C Differences in contact probability $\Delta p_{i,j} = p_{i,j}(Wt) - p_{i,j}(12D)$ from simulations with implicit solvent HPS coarse-grained model. Black and purple arrows indicate positions of aromatic residues and sites of S-to-D substitutions respectively.

D Excess free energy of transfer ΔG_{trans} from the dilute phase to the condensate computed from the saturation and condensate densities of coexisting phases in simulations with the HPS coarse-grained model for different TDP-43 LDC variants. ΔG_{trans} could not be determined reliably for the 12pS LCD (N.D. = not determined).

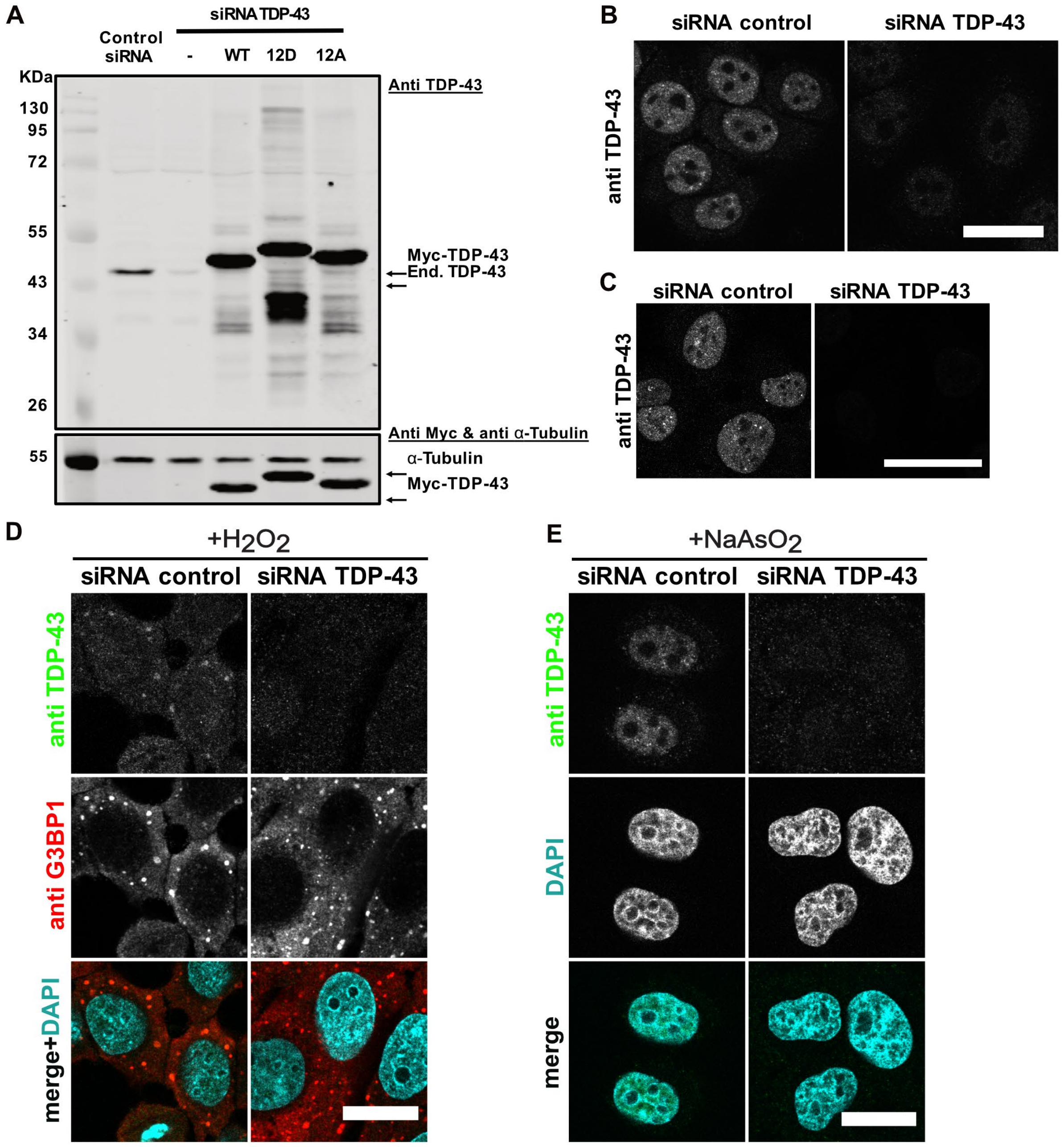

Appendix Figure S6. Controls showing endogenous TDP-43 levels after siRNA-mediated knockdown.

A SDS-PAGE followed by Western blot showing efficient siRNA-mediated knockdown of endogenous TDP-43 (running at ~43 kDa) and re-expression of myc-tagged TDP-43 Wt, 12D and 12A in Hela cells. Equal loading is demonstrated by α-Tubulin Western blot (bottom). TDP-43 was detected using rabbit anti-TDP-43 C-term antibody (Proteintech), α-Tubulin using mouse anti-alpha Tubulin antibody (Proteintech) and Myc-TDP-43 using mouse-anti Myc antibody (9E10, Helmholtz Center Munich).

B Representative confocal images of HeLa cells showing efficient siRNA-mediated knockdown of endogenous TDP-43 versus control siRNA transfection (control for experiment shown in Fig. 5D). TDP-43 (grey) was detected using a mouse anti-TDP-43 antibody (Proteintech). Bar, 25 µm.

C Representative confocal images of a Flp-In T-Rex U2OS stable cell line showing efficient siRNA-mediated knockdown of endogenous TDP-43 versus control siRNA transfection

(control for experiment shown in Fig. EV4C). TDP-43 (grey) was detected using a mouse anti-TDP-43 antibody (Proteintech). Bar, 40 µm.

D, E Representative confocal images of HeLa cells showing endogenous TDP-43 (grey, upper rows) in control siRNA-treated (left column) or TDP-43 siRNA-treated cells (right column) after H_2O_2 (C) or arsenite (D) stress (control for experiment shown in Fig. 5D and 5H). TDP-43 was detected using mouse anti-TDP-43 antibody (Proteintech), SGs using rabbit anti G3BP1 antibody (Proteintech) and nuclei were stained with DAPI. For clarity, signals were converted to grey values in the individual channels (upper two rows). In the merge (lower row), DAPI is shown in turquoise, TDP-43 in green and G3BP1 in red. Bar, 20 µm.

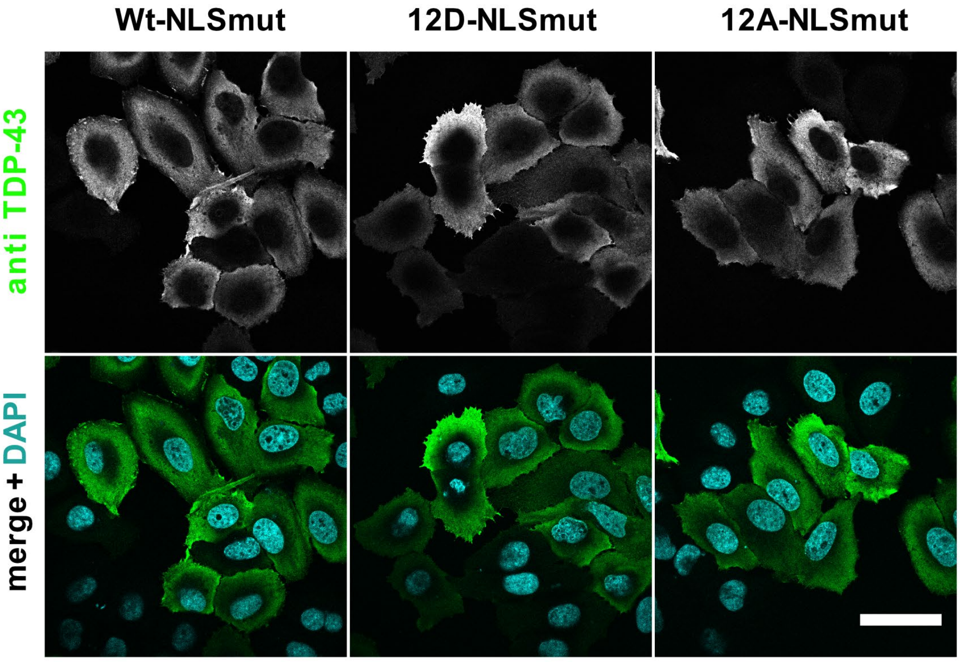

Appendix Figure S7. Control experiment for localization of NLS mutant TDP-43 variants in HeLa cells.

Representative confocal images of HeLa cells demonstrating equal cytoplasmic localization of all TDP-43-NLS mutant variants (Wt, 12D and 12A), 24 h after transfection and 48 h after endogenous TDP-43 silencing. Staining was carried out with a mouse anti-TDP-43 antibody (Proteintech), TDP-43 signal is shown in grey (upper row) or green (lower row); DAPI is shown in turquoise. Bar, 40 µm.

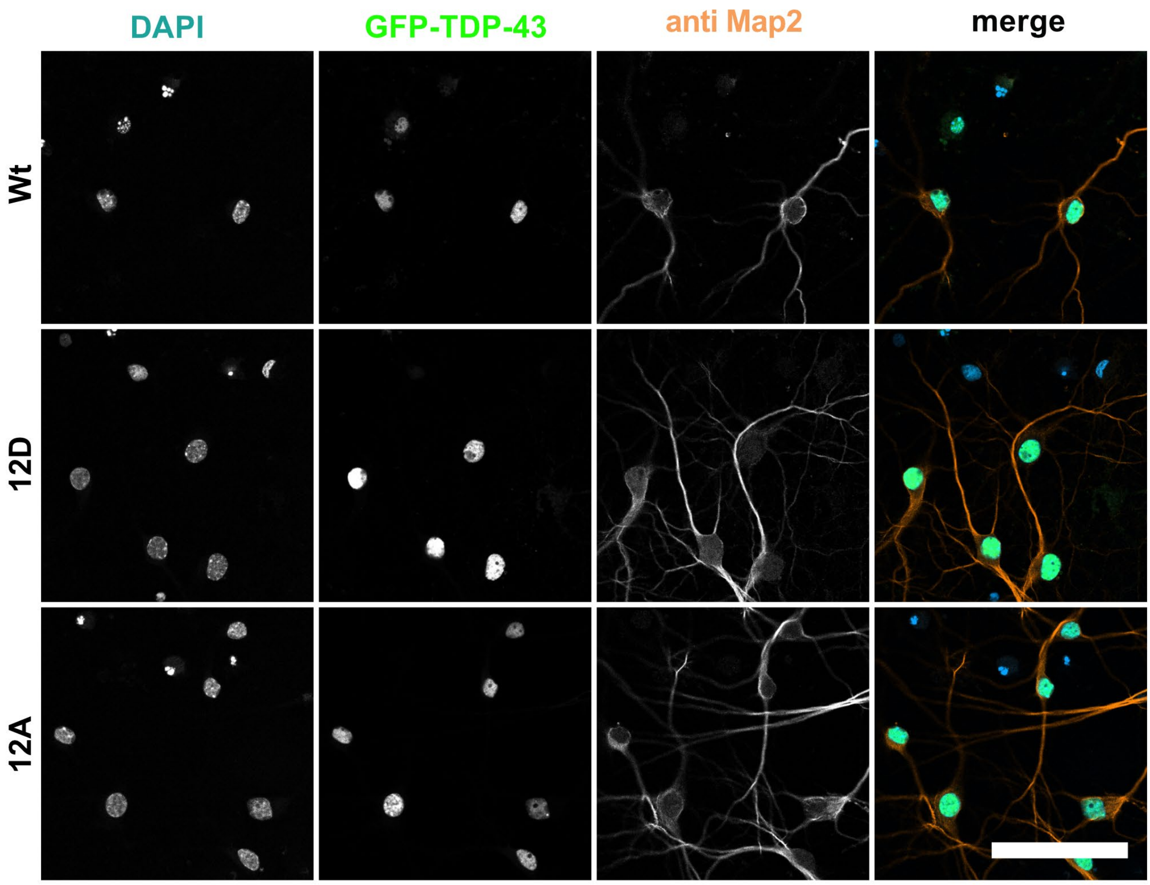

Appendix Figure S8. Control experiment for localization of EGFP-TDP-43 variants in primary hippocampal neurons.

Representative confocal images displaying nuclear localization of all EGFP-TDP-43 variants (Wt, 12D and 12A) in primary rat hippocampal neurons. Neurons were transduced on DIV 5 and stained on DIV5+4, with mouse anti-Map2 antibody and nuclei with DAPI. For clarity, signals were converted to grey values in the individual channels (first three columns). In the merge (fourth column), nuclei are shown in turquoise, EGFP-TDP-43 in green and Map2 (marker for neuronal cell body and dendrites) in orange. Bar, 80 µm.

Appendix Table S1. Concentrations of dense and dilute phases in simulations with the HPS coarse-grained model.

Uncertainties were estimated from block averaging. For 12pS the concentrations were not determined (N.D.), as it forms multiple clusters rather than a single dense-phase condensate.

	c_{dense} [mM]	c_{dilute} [mM]
Wt	34.546 +/- 0.026	0.007 +/- 0.002
12D	31.498 +/- 0.031	0.023 +/- 0.002
5pS	33.340 +/- 0.029	0.008 +/- 0.002
12pS (not a condensate)	N.D.	N.D.
12A	38.255 +/- 0.021	0.0005 +/- 0.0001
