# Supplementary material for: Disease‐linked TDP‐43 hyperphosphorylation suppresses TDP‐43 condensation and aggregation
Source: EMBO J. 2022 Feb 3;41(8):e108443. doi: 10.15252/embj.2021108443 (PMC9016352; doi:10.15252/embj.2021108443)
Supplement: Supplementary file 2 — Expanded View Figures PDF [file EMBJ-41-e108443-s007.pdf]

Expanded View Figures

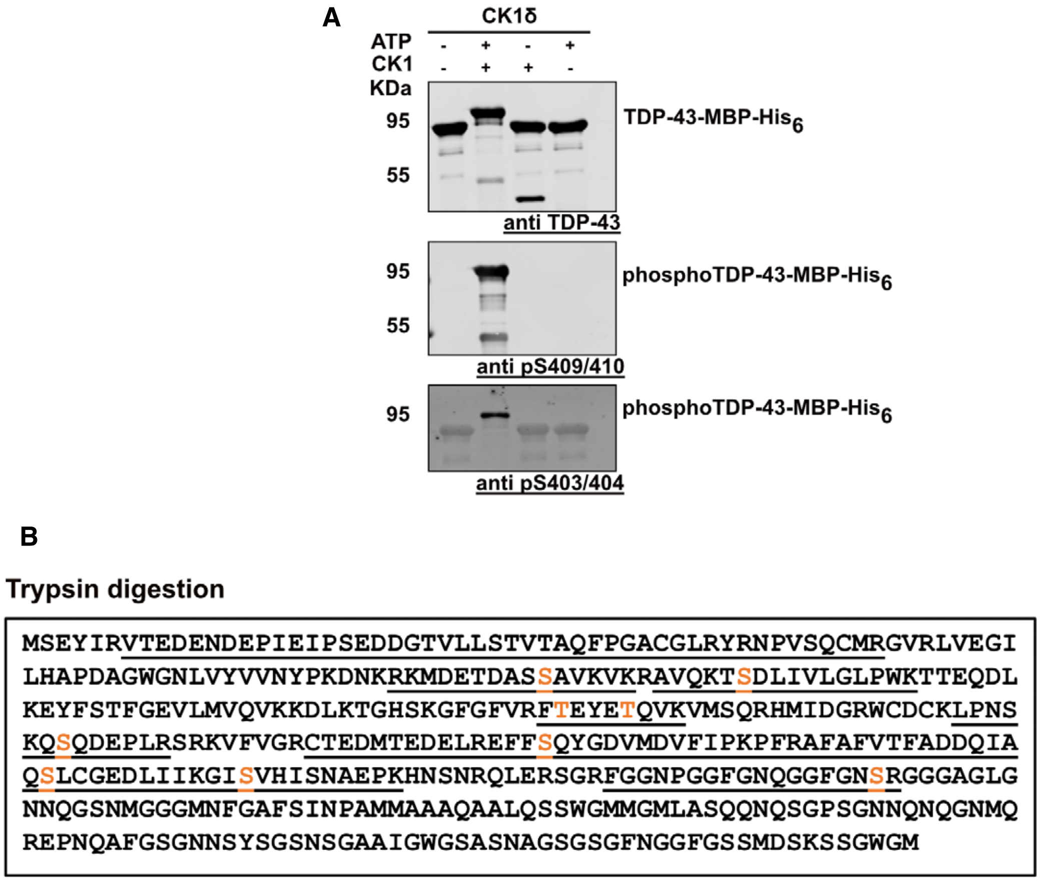

Figure EV1. Identification of TDP-43-MBP-His$_6$ phospho-sites after *in vitro* phosphorylation with CK1δ.

A Identification of TDP-43 phospho-sites on *in vitro* phosphorylated TDP-43 (+CK1δ, +ATP) in comparison to controls (−CK1δ −ATP; CK1δ only; ATP only) by Western blot. Samples were analyzed by SDS–PAGE and Western blot using a rabbit anti-TDP-43 N-term antibody (Proteintech) to detect total TDP-43, rat anti-TDP-43-phospho Ser409/410 (clone 1D3, Helmholtz Center Munich) and mouse anti-TDP-43-phospho Ser403/404 (Proteintech, Cat. No.: 66079-1-Ig) antibodies.

B Schematic diagrams showing sequence coverage in mass spectrometry after trypsin digest (underlined) and phosphorylated serine/threonine residues (orange) of *in vitro* phosphorylated TDP-43-MBP-His$_6$ with CK1δ + ATP (one out of two representative experiments is shown).

Source data are available online for this figure.

© 2022 The Authors

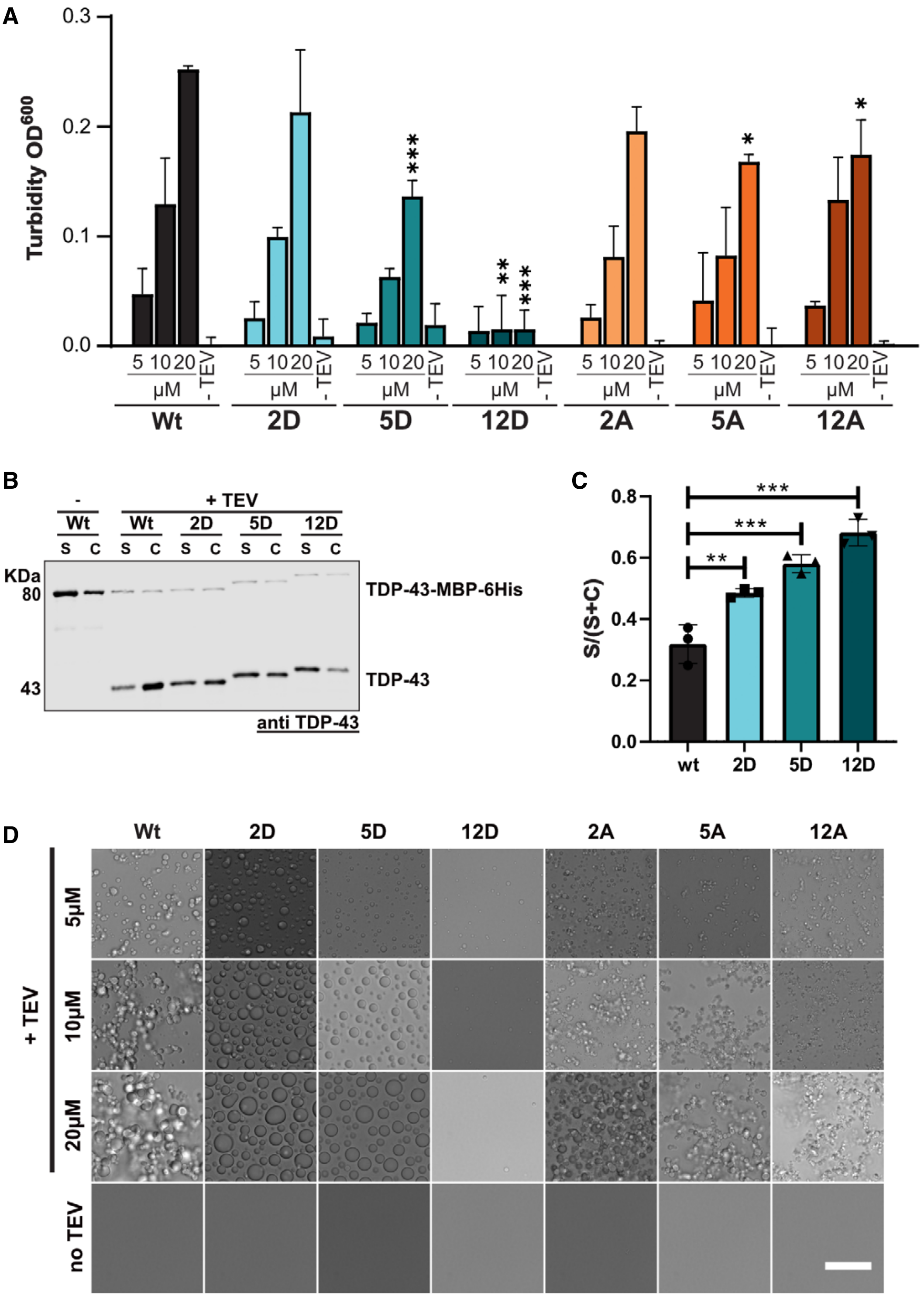
A
Turbidity OD600
0.3
0.2
0.1
0.0
5 10 20
μM
- TEV
Wt
2D
5D
12D
2A
5A
12A
B
- Wt
+ TEV
Wt 2D 5D 12D
S C
KDa
80
43
TDP-43-MBP-6His
TDP-43
anti TDP-43
C
S/(S+C)
0.8
0.6
0.4
0.2
0.0
wt 2D 5D 12D
D
Wt 2D 5D 12D 2A 5A 12A
+ TEV
5μM
10μM
20μM
no TEV

Figure EV2.

Figure EV2. C-terminal phosphomimetic substitutions reduce TDP-43 condensation *in vitro*.

A Turbidity measurements (optical density [OD] at 600 nm) to quantify phase separation of different S-to-D and S-to-A mutants in comparison to TDP-43 Wt using phosphate buffer. Values represent mean of three independent experimental replicates ($n = 3$) ± SD. *$P < 0.0332$, **$P < 0.0021$ and ***$P < 0.0002$ by one-way ANOVA with Dunnett's multiple comparison test to Wt, comparing the respective concentration condition (5, 10, 20 μM).

B Sedimentation assay to quantify condensation of different S-to-D mutants in comparison to TDP-43 Wt (in Hepes buffer). TDP-43 was detected by TDP-43 Western blot (rabbit anti-TDP-43 N-term).

C Quantification of band intensities of cleaved TDP-43 corresponding to supernatant (S) and condensates (C) fractions is shown as mean of S/(S + C) ratio of three independent experimental replicates ($n = 3$) ± SD. **$P < 0.0021$ and ***$P < 0.0002$ by one-way ANOVA with Dunnett's multiple comparison test to Wt.

D Representative bright field microscopic images of TDP-43 condensates formed from TDP-43 Wt vs different S-to-D or S-to-A variants in phosphate buffer (Bar, 25 μm).

Source data are available online for this figure.

© 2022 The Authors

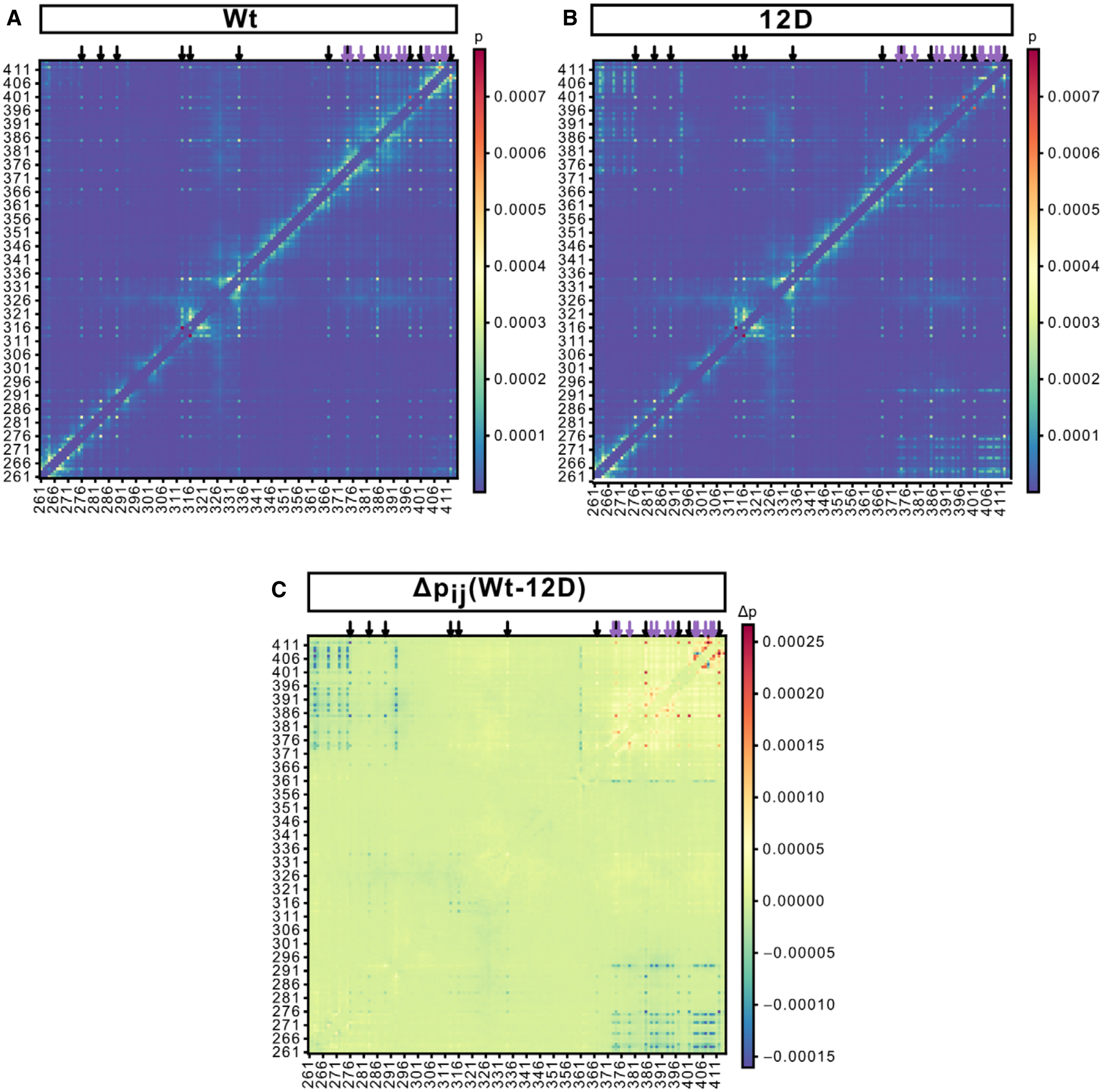

Figure EV3. Analysis of contacts in biomolecular condensates formed by the TDP-43 LCD in coarse-grained simulations.

A, B Contact maps for Wt (A) and 12D (B) TDP-43 LCD from simulations with the explicit solvent Martini coarse-grained model. Residue i and residue j are defined to be in contact if any of the coarse-grained beads are within 4.5 Å. The relative contact probability is calculated by averaging over all 118 protein chains and the last 5 of 20 μs simulations each. Intra-chain contacts with the two preceding and following residues are excluded from the analysis. Aromatic residues form prominent contacts and are highlighted by black arrows. For example, looking at the column for F276 and following it upwards one can see that F276 interacts with F276 in other chains and irrespective of the chain, with F283, F289, F313, F316, W334, F367, Y347, W385, F401, and W412. The sites of the phosphomicking S-to-D mutations are highlighted by purple arrows. At these sites differences between Wt and 12D LCD can be seen, with Wt forming more contacts close in protein sequence and 12D instead interacting with R268, R272, R275, R293, and R361 further away in the sequence.

C Differences in contact probability $P_{i,j} = P_{i,j}(\mathrm{Wt}) - P_{i,j}(\mathrm{12D})$ from simulations with the explicit-solvent Martini coarse-grained model. Differences highlight that wild-type S residues, unlike phosphomicking D residues, favor interactions with residues close in sequence, while demonstrating that most contacts are not affected by the phosphomicking S-to-D mutations. Black and purple arrows correspond to aromatic residues and phosphomicking S-to-D mutations, respectively.

© 2022 The Authors

Figure EV4. Phosphomimetic substitutions do not alter nuclear localization, UG-rich RNA binding and autoregulation of TDP-43.

A Immunostainings showing nuclear localization of myc-TDP-43 Wt, 12D and 12A in HeLa cells. Endogenous TDP-43 expression was silenced by siRNAs, followed by transient transfection of the indicated siRNA-resistant myc-TDP-43 constructs. After 24 h, localization of TDP-43 Wt, 12D and 12A variants was visualized by TDP-43 immunostaining (mouse anti-TDP-43 antibody, Proteintech). G3BP1 (rabbit anti-G3BP1 antibody, Proteintech) and DAPI signal is shown to visualize the cytoplasm and nuclei, respectively. In the merge (right column), DAPI is show in turquoise, TDP-43 in green, and G3BP1 in magenta. Bar, 30 μm.

B Electrophoretic mobility shift assay (EMSA) of TDP-43-MBP-His_6 variants (Wt, 12D and 12A) in a complex with $(UG)_{12}$ RNA.

C Representative confocal images of U2OS cells stably expressing the indicated myc-TDP-43 variants (Wt, 12D and 12A) after siRNA KD of endogenous TDP-43 and induction of myc-TDP-43 expression with doxycycline. Cells were stained with mouse monoclonal anti-myc 9E10 antibody (IMB protein production facility) and DAPI. For clarity, signals were converted to grey values in the individual channels (upper two rows). In the merge (lower row), DAPI is shown in turquoise) and myc-TDP-43 is shown in green. Bar, 20 μm.

D Western Blot showing the expression levels of myc-TDP-43 variants in stable inducible Flp-In T-Rex U2OS cell lines before and after addition of doxycycline (dox). Samples were analyzed by SDS–PAGE and Western blot using a rabbit anti-TDP-43 N-term antibody (Proteintech, upper blot), mouse anti-myc 9E10 antibody (IMB protein production core facility), and rabbit anti-Histone H3 antibody (Abcam) to detect the loading control Histone H3.

E Quantification of TDP-43 autoregulation after dox-induced expression of myc-TDP-43 variants in U2OS cell lines. Values represent the mean ± SD of four independent experimental replicates ($n = 4$) of endogenous TDP-43 expression levels normalized to Wt (−Dox) condition. *$P < 0.0332$ and ***$P < 0.0002$ by one-way ANOVA with Šídák's multiple comparisons test of TDP-43 endogenous expression levels, comparing the respective non-induced (−Dox) and induced (+Dox) lines.

Source data are available online for this figure.

 © 2022 The Authors

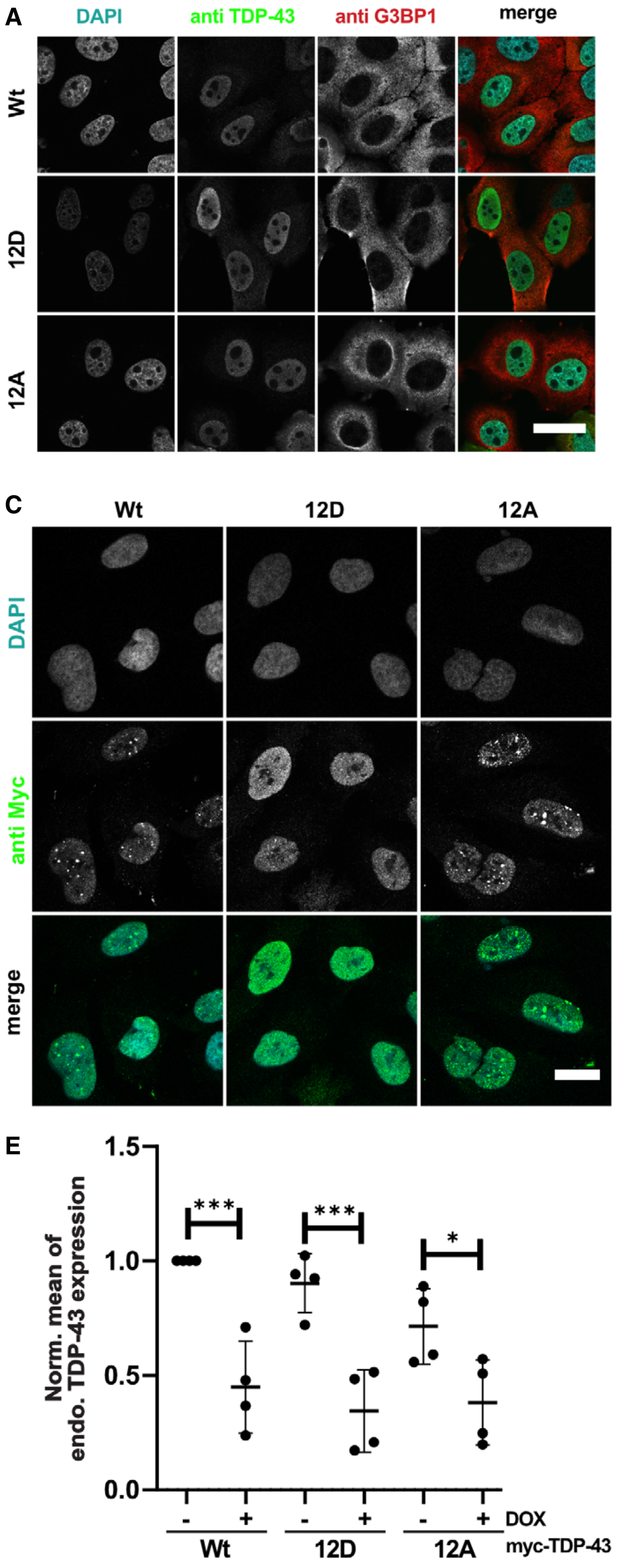
A
DAPI
anti TDP-43
anti G3BP1
merge
Wt
12D
12A
B
Wt
12D
12A
0
TDP-43-RNA
Free RNA
UG12
24 nts
No secondary structure
C
Wt
12D
12A
DAPI
anti Myc
merge
D
- DOX
+ DOX
Wt
12D
12A
Wt
12D
12A
55
43
55
43
17
anti TDP-43
Myc-TDP-43
End TDP-43
anti Myc
Myc-TDP-43
Histone H3
E
Norm. mean of endo. TDP-43 expression
1.5
1.0
0.5
0.0
***
***
*
-
+
-
+
-
+
DOX
Wt
12D
12A
myc-TDP-43

Figure EV4.

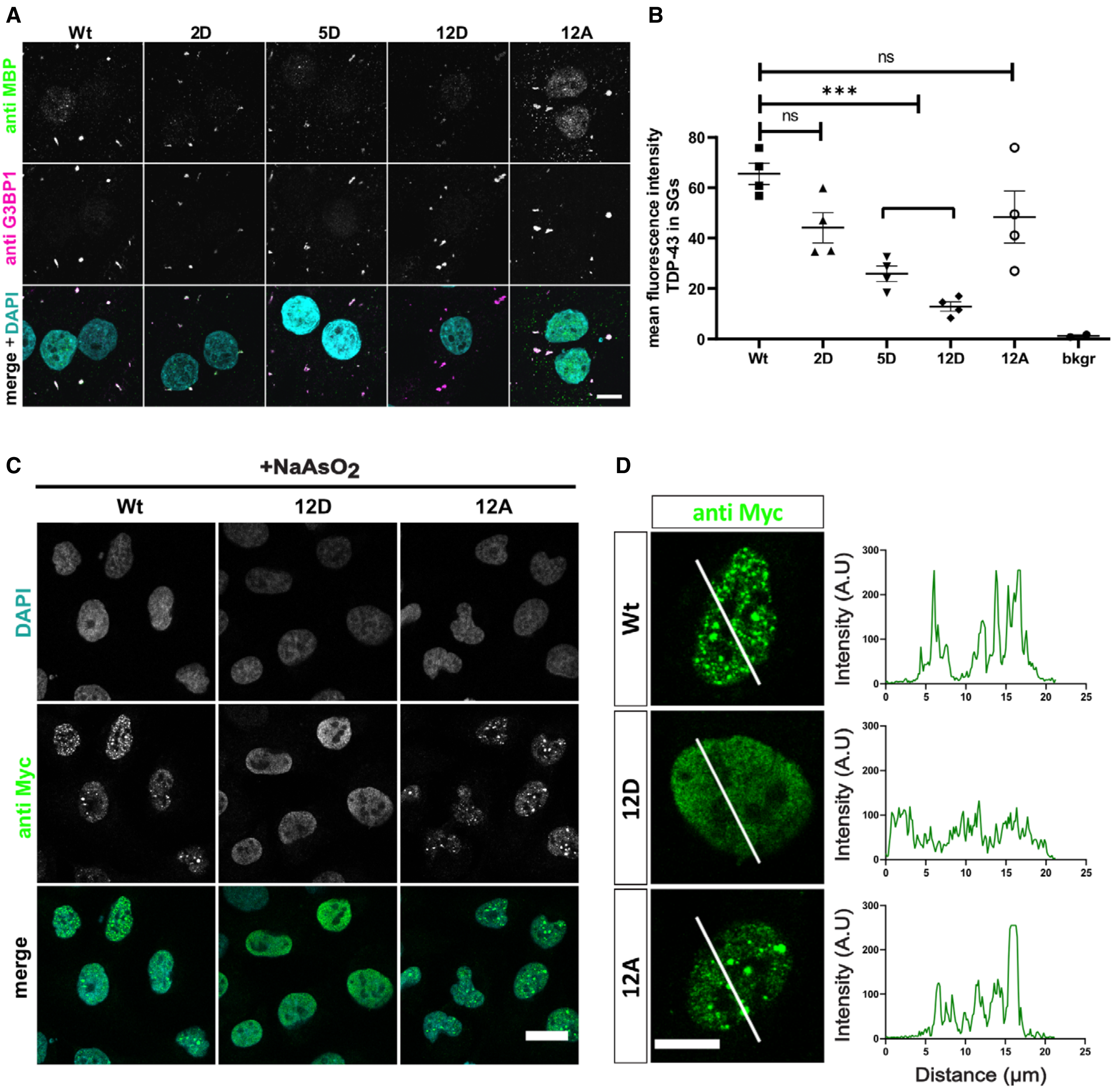

Figure EV5. Phosphomimetic S-to-D substitutions reduce association of TDP-43 with stress granules and nuclear stress bodies.

A Association of TDP-43 with stress granules (SGs) in semi-permeabilized HeLa cells is suppressed by phosphomimetic (2D, 5D and 12D) mutations in comparison to TDP-43 Wt and 12A. SGs and TDP-43-MBP-His_6 were visualized by G3BP1 and MBP immunostaining, respectively. For clarity, signals were converted to grey values in the individual channels (upper two rows). In the merge (lower row), G3BP1 is shown in magenta, TDP-43-MBP-His_6 in green, white pixels indicate colocalization. Nuclei were counterstained with DAPI (turquoise). Bar, 10 μm.

B Quantification of the mean fluorescence intensity of TDP-43-MBP-His_6 in SGs normalized to Wt for four independent replicates ± SEM, ***$P < 0.0002$ defined by 1-way ANOVA with Dunnett's multiple comparison test (≥ 10 cells; ≥ 46SGs per condition). Bkgr = background fluorescence in the green channel.

C Representative confocal images of U2OS cells stably expressing the indicated myc-TDP-43 variants (Wt, 12D and 12A) after siRNA KD of endogenous TDP-43 and induction of myc-TDP-43 expression with doxycycline, followed by sodium arsenite stress (2 h) to induce nuclear stress bodies (NBs). Cells were stained with mouse monoclonal anti-myc 9E10 antibody (IMB protein production facility) and DAPI. For clarity, signals were converted to grey values in the individual channels (upper two rows). In the merge (lower row), DAPI is shown in turquoise and myc-TDP-is shown in green. Bar, 20 μm.

D Intensity profiles (right) of myc-TDP-43 Wt, 12D and 12A variants (green), expressed in Flp-In T-Rex USOS stable cell lines, along white lines (left). Bar, 10 μm.

 © 2022 The Authors
