# Supplementary material for: Disease‐linked TDP‐43 hyperphosphorylation suppresses TDP‐43 condensation and aggregation
Source: EMBO J. 2022 Feb 3;41(8):e108443. doi: 10.15252/embj.2021108443 (PMC9016352; doi:10.15252/embj.2021108443)
Supplement: Supplementary file 3 — Movie EV1 [file EMBJ-41-e108443-s009.zip › EMBOJ-2021-108443R1-MovieEV1/Legend_MovieEV1.docx]

Movie EV1.

Fluorescently labelled TDP-43 Wt condensates imaged live by spinning disc confocal microcopy.
