# Supplementary material for: Disease‐linked TDP‐43 hyperphosphorylation suppresses TDP‐43 condensation and aggregation
Source: EMBO J. 2022 Feb 3;41(8):e108443. doi: 10.15252/embj.2021108443 (PMC9016352; doi:10.15252/embj.2021108443)
Supplement: Supplementary file 7 — Movie EV5 [file EMBJ-41-e108443-s003.zip › EMBOJ-2021-108443R1-MovieEV5/Legend_MovieEV5.docx]

**Movie EV5.**

All-atom simulations of TDP-43 LCD 12D with explicit representation of proteins, ions, and water with atomic resolution.
