# Supplementary figures and images for: Disease‐linked TDP‐43 hyperphosphorylation suppresses TDP‐43 condensation and aggregation
Source: EMBO J. 2022 Feb 3;41(8):e108443. doi: 10.15252/embj.2021108443 (PMC9016352; doi:10.15252/embj.2021108443)

Source data Appendix Fig. S1

Appendix Fig.S1C

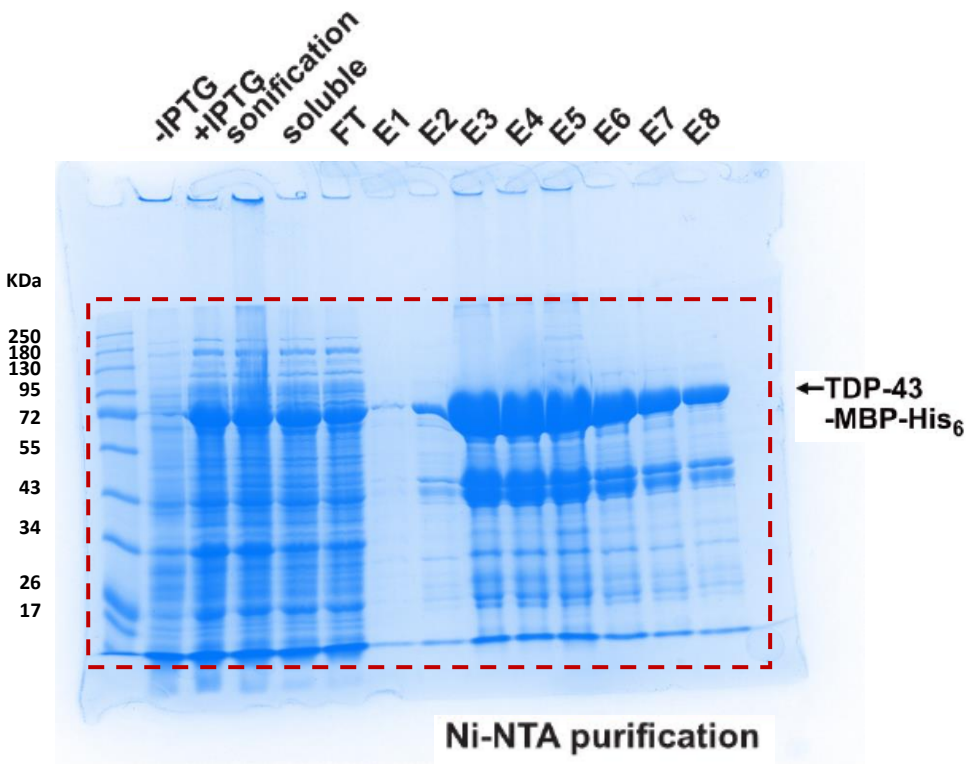

Appendix Fig.S1E

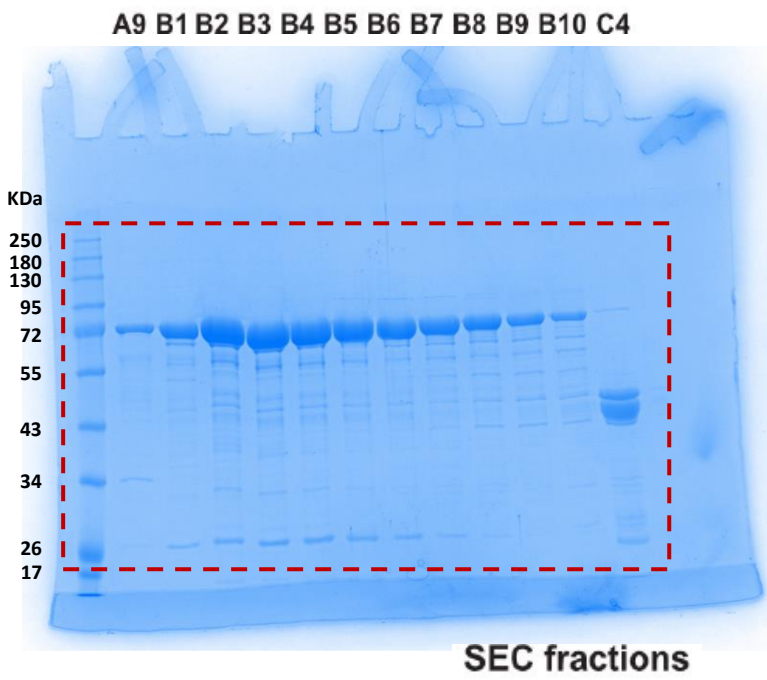

Supplement: Supplementary file 8 — Source Data for Expanded View/Appendix [file EMBJ-41-e108443-s005.zip › Appendix_and_EV_Figure_Source_Data/EMBOJ-2021-108443R1-Figure_Source_Data_Appendix_FigS1-sd.pdf]

Source data Appendix Fig. S3

Appendix Fig.S3

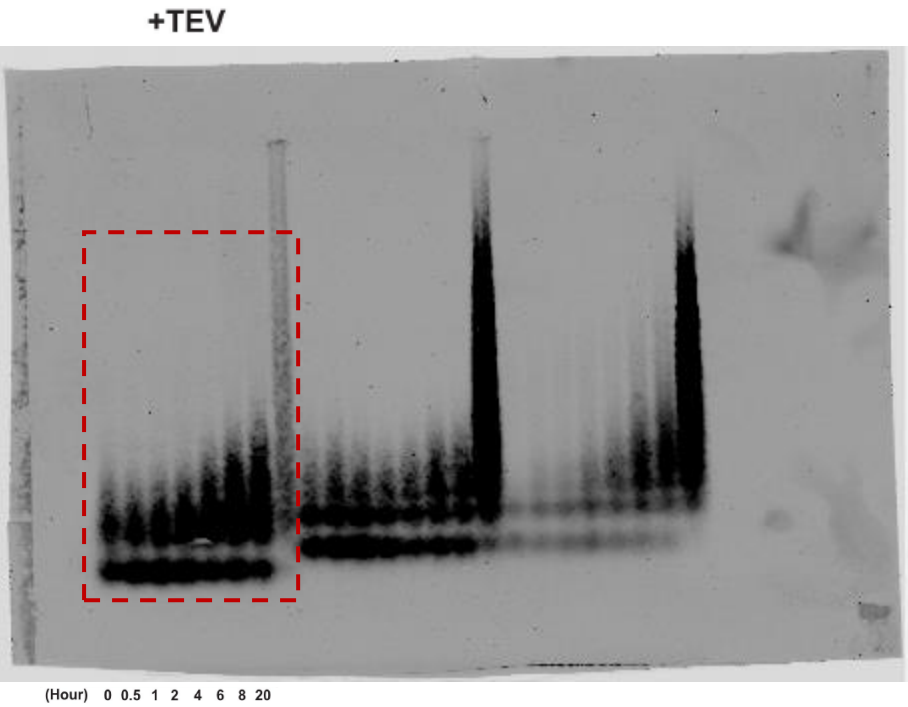

Supplement: Supplementary file 8 — Source Data for Expanded View/Appendix [file EMBJ-41-e108443-s005.zip › Appendix_and_EV_Figure_Source_Data/EMBOJ-2021-108443R1-Figure_Source_Data_Appendix_FigS3-sd.pdf]

Source data Appendix Fig. S6

Appendix Fig.S6A

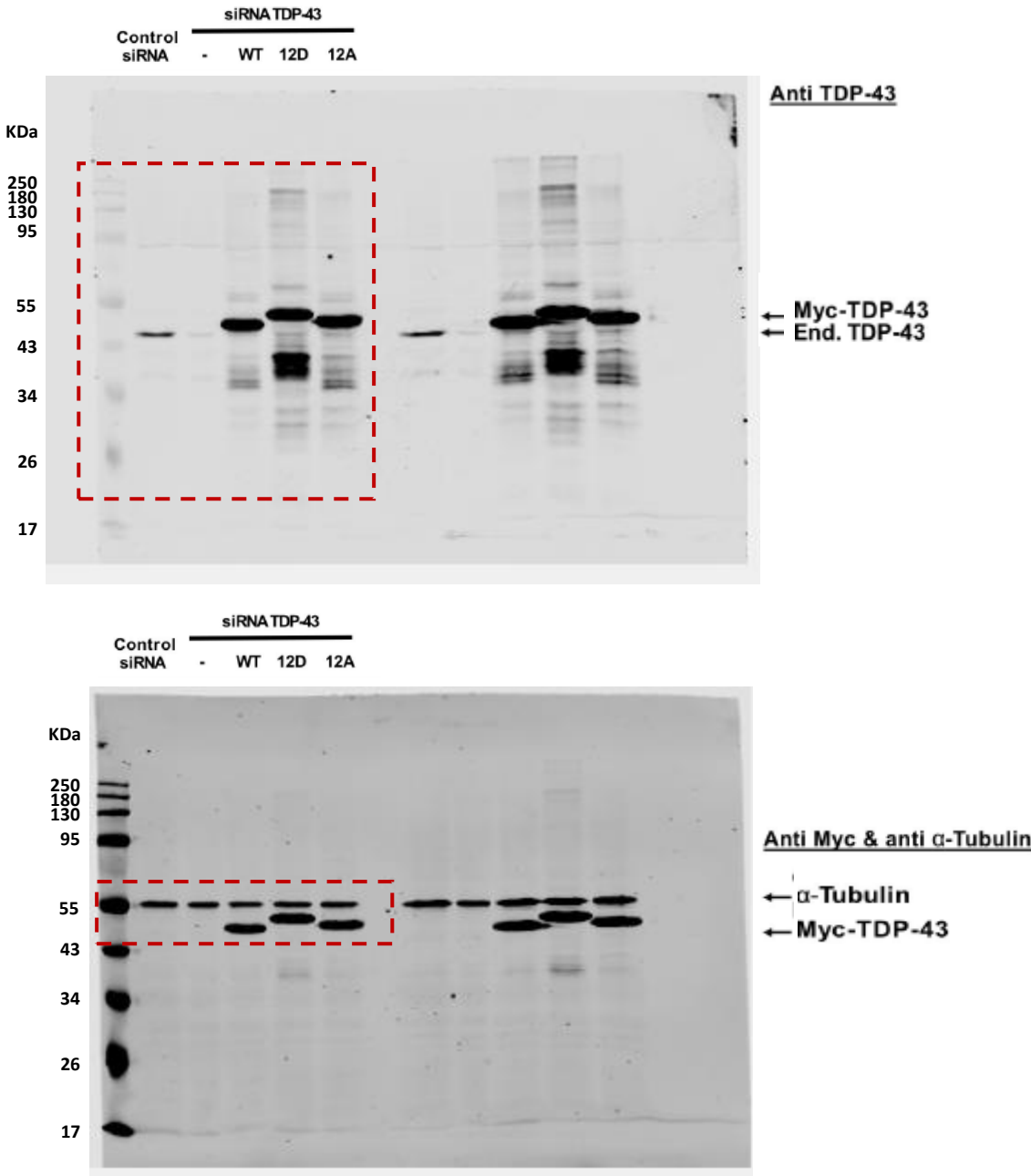

Supplement: Supplementary file 8 — Source Data for Expanded View/Appendix [file EMBJ-41-e108443-s005.zip › Appendix_and_EV_Figure_Source_Data/EMBOJ-2021-108443R1-Figure_Source_Data_Appendix_FigS6-sd.pdf]

Source data Expansion Fig. 1

Exp. Fig. 1A

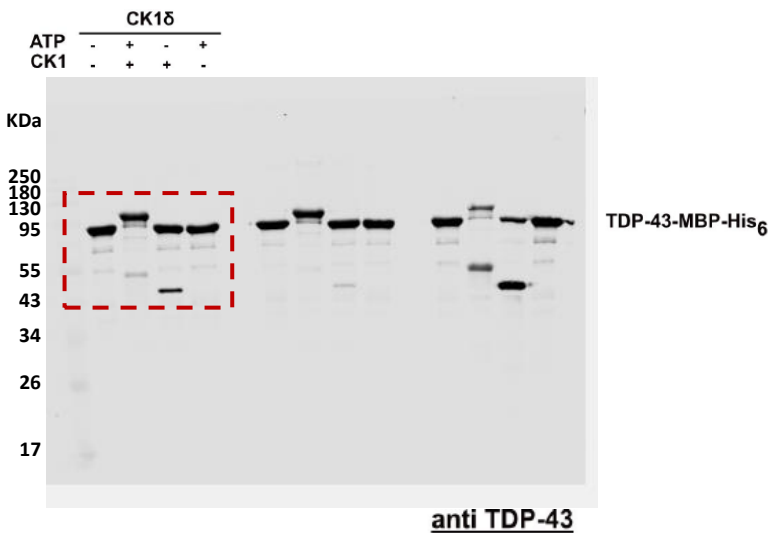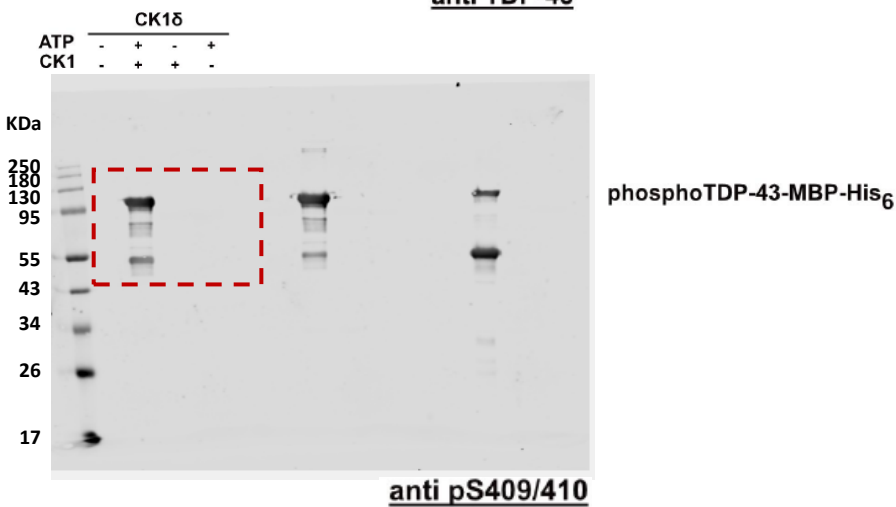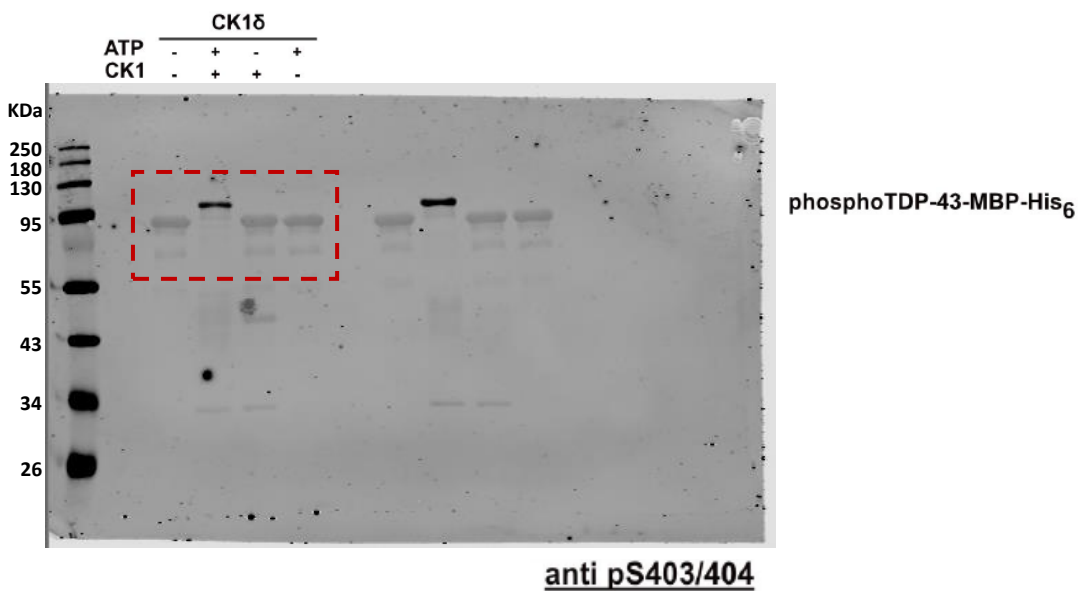

Supplement: Supplementary file 8 — Source Data for Expanded View/Appendix [file EMBJ-41-e108443-s005.zip › Appendix_and_EV_Figure_Source_Data/EMBOJ-2021-108443R1-Figure_Source_Data_figEV1-sd.pdf]

Source data Expansion Fig. 2

Exp. Fig. 2B

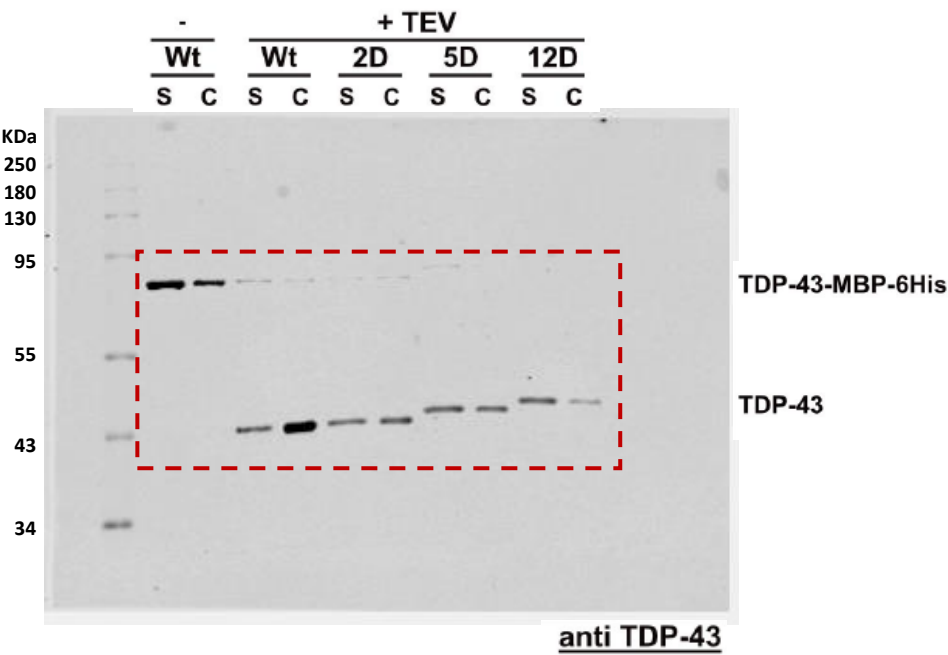

Supplement: Supplementary file 8 — Source Data for Expanded View/Appendix [file EMBJ-41-e108443-s005.zip › Appendix_and_EV_Figure_Source_Data/EMBOJ-2021-108443R1-Figure_Source_Data_FigEV2-sd.pdf]

Source data Expansion Fig. 4

Exp. Fig. 4D

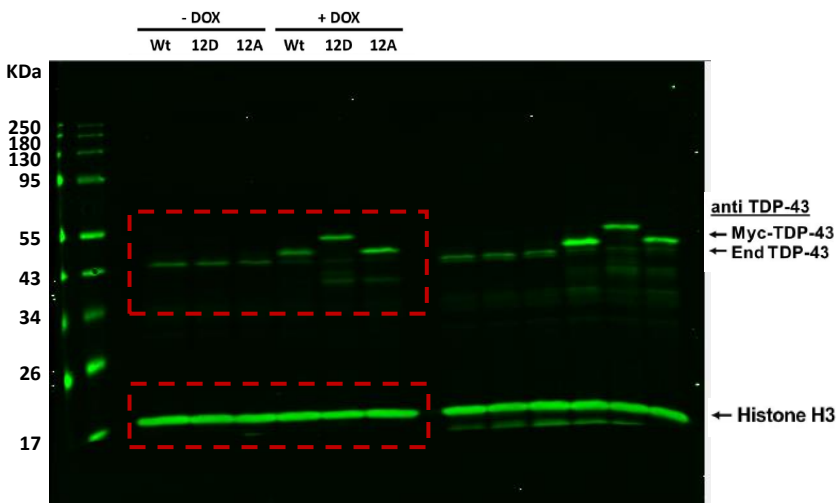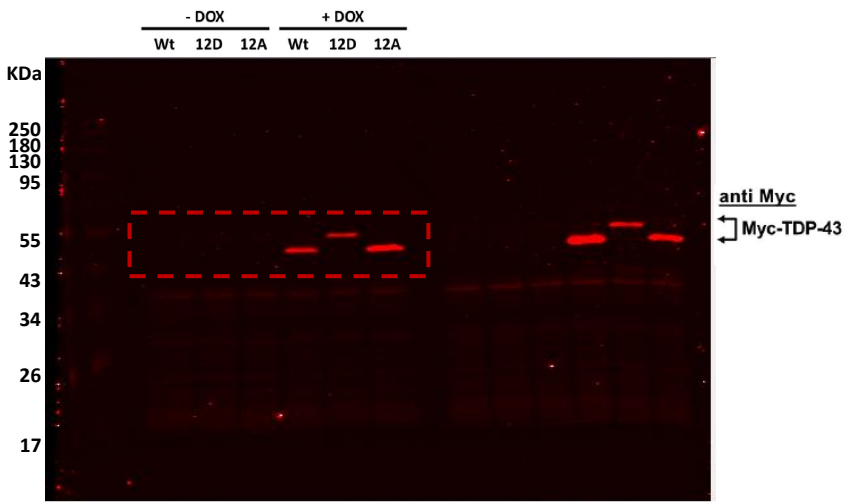

Supplement: Supplementary file 8 — Source Data for Expanded View/Appendix [file EMBJ-41-e108443-s005.zip › Appendix_and_EV_Figure_Source_Data/EMBOJ-2021-108443R1-Figure_Source_Data_FigEV4-sd.pdf]

Source data Fig. 1

Fig. 1B

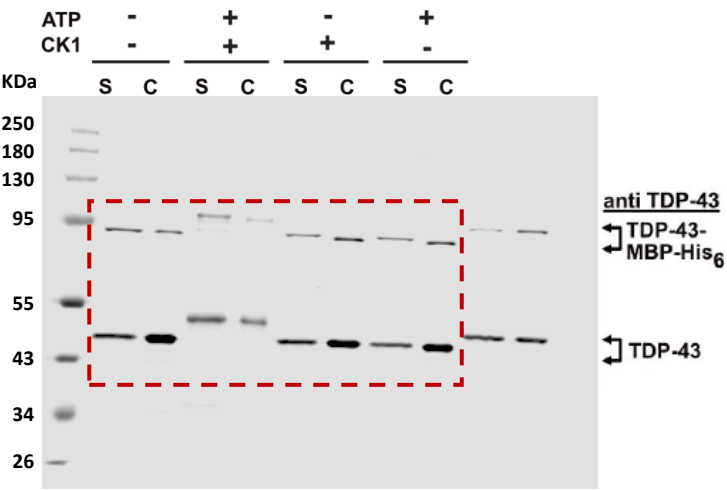

Supplement: Supplementary file 9 — Source Data for Figure 1 [file EMBJ-41-e108443-s010.pdf]

Source data Fig. 2

Fig. 2E

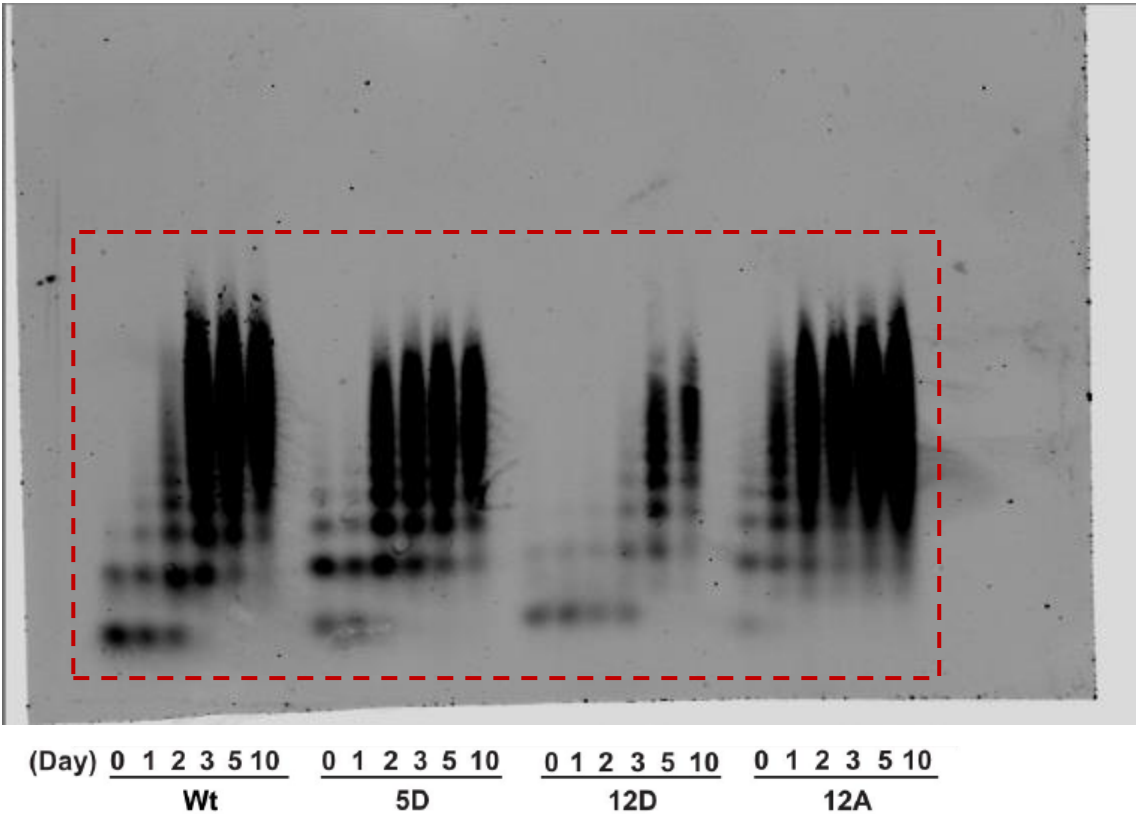

Fig. 2F

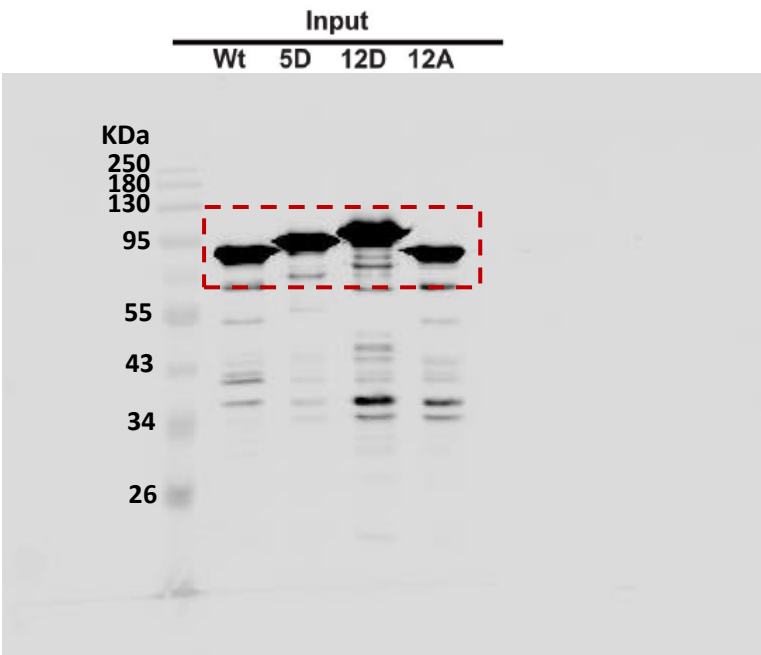

Supplement: Supplementary file 10 — Source Data for Figure 2 [file EMBJ-41-e108443-s012.pdf]

Source data Fig. 4

Fig. 4C

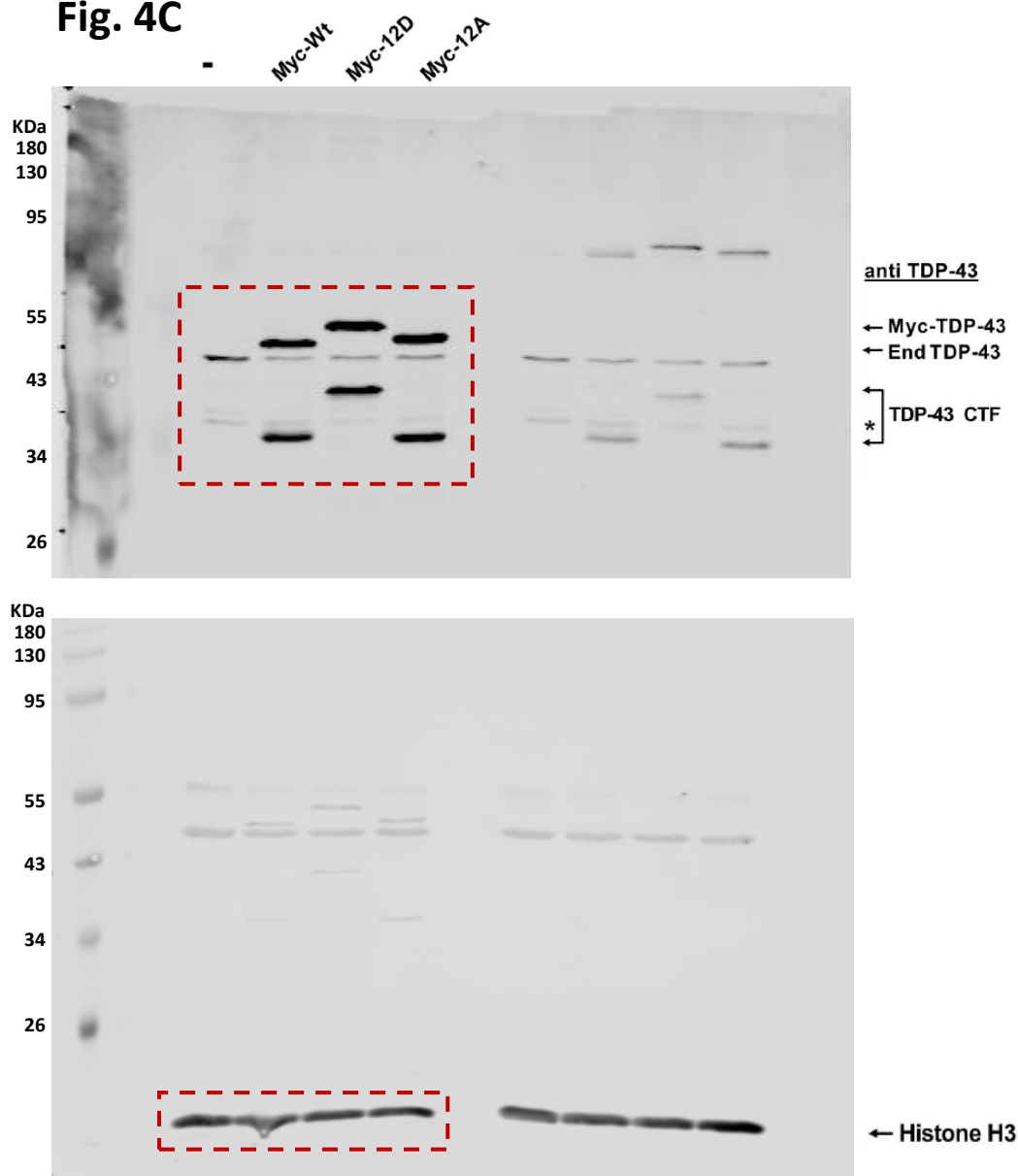

Fig. 4E

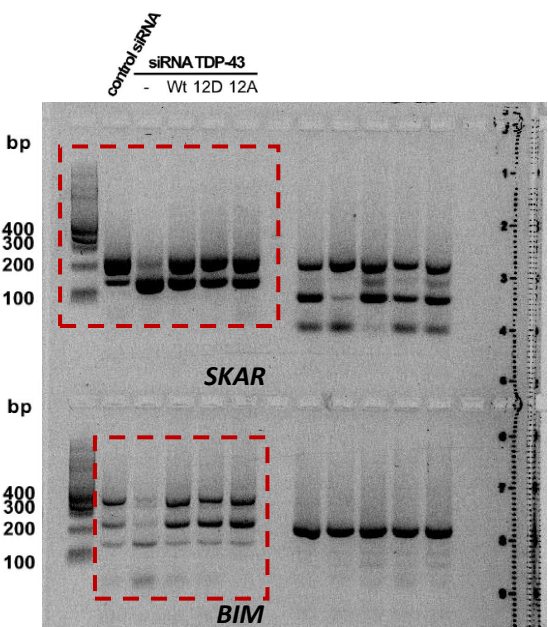

Supplement: Supplementary file 11 — Source Data for Figure 4 [file EMBJ-41-e108443-s004.pdf]

Source data Fig. 6

Fig. 6A

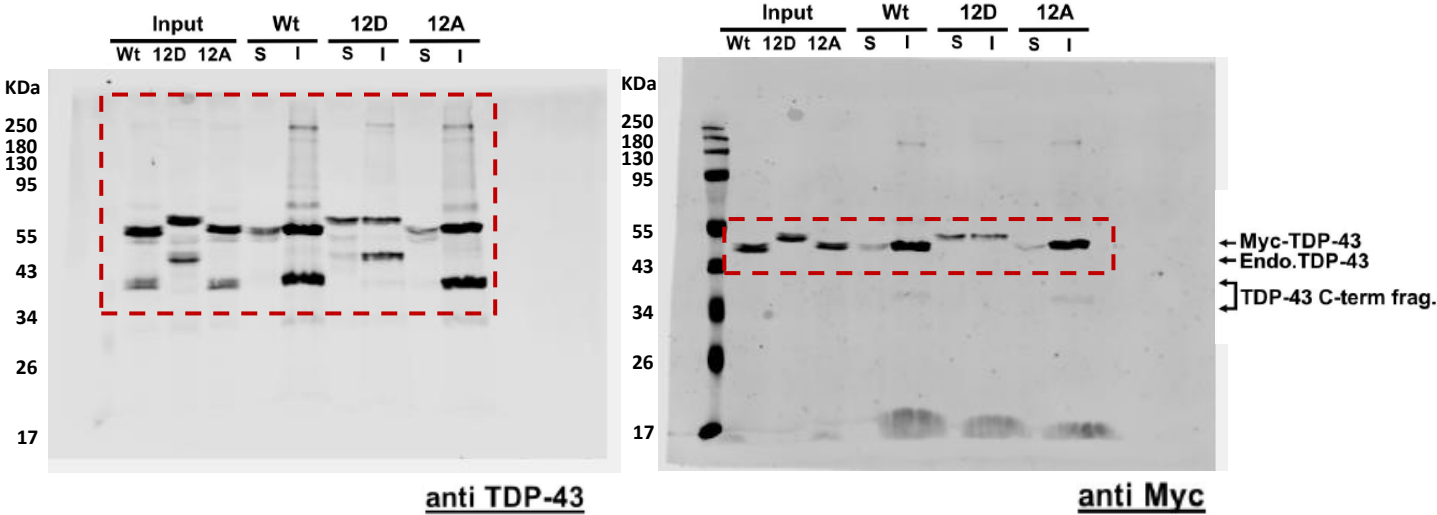

Fig. 6C

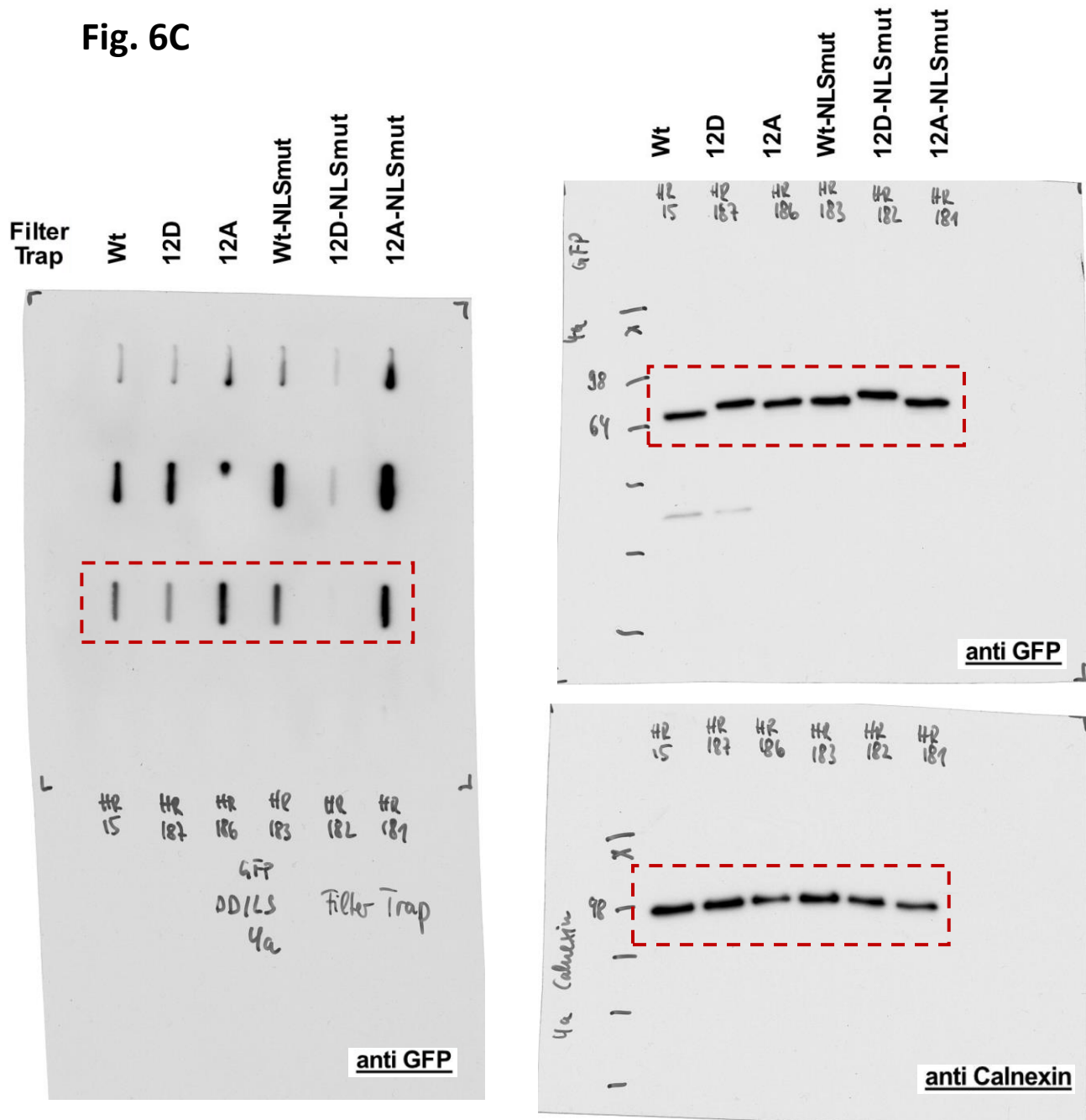

Supplement: Supplementary file 12 — Source Data for Figure 6 [file EMBJ-41-e108443-s008.pdf]
