# Supplementary material for: Disease‐linked TDP‐43 hyperphosphorylation suppresses TDP‐43 condensation and aggregation
Source: EMBO J. 2022 Feb 3;41(8):e108443. doi: 10.15252/embj.2021108443 (PMC9016352; doi:10.15252/embj.2021108443)
Supplement: Supplementary file 13 [file EMBJ-41-e108443-s013.pdf]

THE EMBO JOURNAL

Disease-linked TDP-43 hyperphosphorylation suppresses TDP-43 condensation and aggregation

Lara Aletta Gruijs da Silva, Francesca Simonetti, Saskia Hutten, Henrick Riemenschneider, Erin Sternburg, Lisa Pietrek, Jokob Gebel, Volker Dötsch, Dieter Edbauer, Gerhard Hummer, Lukas Stelzl, and Dorothee Dormann
DOI: 10.15252/embj.2021108443

Corresponding author(s): Dorothee Dormann (ddormann@uni-mainz.de)

Review Timeline:

Submission Date:	15th Apr 21
Editorial Decision:	25th May 21
Revision Received:	28th Oct 21
Editorial Decision:	15th Dec 21
Revision Received:	23rd Dec 21
Accepted:	4th Jan 22

Editor: Karin Dumstrei

Transaction Report:

(Note: With the exception of the correction of typographical or spelling errors that could be a source of ambiguity, letters and reports are not edited. Depending on transfer agreements, referee reports obtained elsewhere may or may not be included in this compilation. Referee reports are anonymous unless the Referee chooses to sign their reports.)

1st Editorial Decision 25th May 2021

Dear Dorothee,

Thank you for submitting your manuscript to The EMBO Journal. Your study has now been seen by three referees and their comments are provided below.

As you can see from the comments, the referees find the analysis interesting and suitable for publication here. I am therefore happy to invite you to submit a suitably revised manuscript. The referee raise a number of different issues that shouldn't be too difficult to sort out. I think it might be helpful to discuss the raised points further and I am available to do so via email or video.

When preparing your letter of response to the referees' comments, please bear in mind that this will form part of the Review Process File, and will therefore be available online to the community. For more details on our Transparent Editorial Process, please visit our website: https://www.embopress.org/page/journal/14602075/authorguide#transparentprocess

Please see the attached PDF for helpful tips when you submit the revised version.

I thank you for the opportunity to consider your work for publication. I look forward to discussing the revisions further.

with best wishes

Karin

Karin Dumstrei, PhD
Senior Editor
The EMBO Journal

Revision to The EMBO Journal should be submitted online within 90 days. If you need an extension please contact the editor; please click on the link below to submit the revision online before 23rd Aug 2021:

https://emboj.msubmit.net/cgi-bin/main.plex

Referee #1:

"Disease-linked TDP-43 hyperphosphorylation suppresses TDP-43 condensation and aggregation", by da Silva et al. 2021, EMBOJ

The RNA-binding protein TDP-43, is the major type of aggregated protein in ALS and FTD, two neurodegenerative disorders. Several C -terminal serine residues of TDP-43 are hyperphosphorylated in disease. In this work, da Silva and colleagues show that phosphorylation of TDP-43 mitigates its aggregation using in vitro and in silico approaches. Da Silva demonstrate that hyperphosphorylation reduces TDP-43 propensity to phase separate and aggregate. These findings are interesting, in part, because TDP-43 phosphorylation was initially assumed to promote, not inhibit, pathological aggregation. Authors suggest that TDP-43 hyperphosphorylation counteract aggregation.

Major concerns

In vitro analysis and simulations (5 items).
(1) This reviewer is not an expert in analysis and simulations.
(2) Quantify rigorously the multiscale simulations of TDP-43 LCD, and the Differences in subcellular behavior of Wt versus 12D TDP-43 (Figure 6D), which is not supported by any quantification.
(3) Do simulations predict that 12D proteins are less aggregation-prone, as was experimentally demonstrated in Figure 2. **discuss** how well does the model reflect the actual protein behavior?
(4) In simulations (Figure 3) test actual phosphorylated TDP-43 in parallel to mimetics.

Cell biology (8 items)

(1) Definite experiments may resolve literature conflicts at the heart of the manuscript:
There is a wealth of literature about the impact of TDP-43 phosphorylation on its biophysical properties with implications to disease. As authors correctly state, even the idea that phosphorylated TDP-43 reduce aggregation propensity and toxicity was tested in cell lines and Drosophila (Brady, Meng et al., 2011, Li, Yeh et al., 2011). Therefore, additional assessment of this well-studied topic should not be based on additional far approximations. The resolution to the conflicts in literature must depend on

more definitive experimental means. Here, a primary concern that the authors should address in revisions is related to in-cell modeling of TDP-43. Over expression data should be substantiated by endogenous knock-in of the modified residues into the endogenous TARDBP gene.

(2) Shortcoming of modeling phosphorylation through "phosphomimetic" residues:
Significant shortcoming in modeling phosphorylation through "phosphomimetic" residue substitution should be acknowledged in the case that the phosphorylation sites serve particular mechanisms of protein- protein interactions. Some phosphomimetic mutants are unable to recapitulate the binding to adapter proteins (e.g., Durocher et al., Mol Cell 4, Yaffe et al., Cell 91, 961-971 387-394). This should be briefly mentioned, although most in the field do not believe that disordered domains enable precise "binding pocket" type of interactions.
More relevant to is the fact that multisite phosphorylation (=large number of phosphorylation sites) can produce critical changes to protein function through bulk electrostatics. This limitations should be acknowledged in the **discussion**.

This reviewer suggests the authors to consider if they wish to **discuss** the derived hypothesis that the number of phosphorylation sites, but not the exact position of the negative charges is critical for changed activity. Furthermore, an experimental under-appreciation of true charge results from introduction of aspartate substitutions (−1) that does not match the charge introduced by Ser (−2). See also (Strickfaden 10.1016/j.cell.2006.12.032; Perlman 10.1016/j.cell.2011.08.052; McLaughlin & Aderem 10.1016/s0968-0004(00)89042-8).

(3) Phosphorylation protective outcome:
The 'protective' nature of TDP-43 phosphorylation in cells, is not appropriately supported by data. Particularly, there is a leap of faith in connecting biophysical properties of overexpressed protein to patho-physiology. Therefore, the authors should play down the interpretation that phosphorylation is a protective cellular mechanism that counteracts aberrant TDP-43 deleterious outcome, unless they explicitly describe the 'protected' property (by showing what are the downstream implications. For example obvious hypotheses may include differential clearance of different forms of TDP-43 or even further downstream differential death of cells).

(4) protein-protein interactions:
Substantiate experimental evidence in-cells that phosphorylated TDP-43 is less prone to engage in protein interactions, which is currently based primarily on simulations of mimetics. For example, authors can perform TDP-43 pulldown followed by proteomics, comparing unphosphorylated / phosphorylated TDP-43.

(5) Phosphorylation impact on TDP-43 cellular localization:
Reduce overstatement when concluding that hyperphosphorylation of TDP-43 does not impair subcellular localization or RNA regulation, as this is a negative result, tested in 'simple' HeLa cells. This is underscored by several reports that observed endogenous cytoplasmic TDP-43 granules in neurons (Alami et al., 2014 10.1016/j.neuron.2013.12.018; Gopal et al., 2017 10.1073/pnas.1614462114; Wang et al., 2008 10.1111/j.1471-4159.2007.05190.x).

(6) Phosphorylation impact of RNA biology:
TDP-43 targets hundreds of mRNAs and regulates their processing in different ways. Particularly, phosphorylation interferes with splicing function (Li et al., 2017 10.1074/jbc.M116.753913). Thus, it is advised that the preliminary analysis of RNA regulation in the original submission should be either taken out of the revised manuscript. An alternative is to perform an unbiased transcriptomic analysis that meets current standards in the field in neurons.

(7) Concern about TDP-43 liquidity in the overexpression system:
Based on manuscript-associated movies, the condensates remain defined entities and do not fuse to form a single mass with united center of gravity, as biomolecular condensates often do. Based on Ghosh and Zhou (10.1101/2020.06.22.164897) and Alberti (10.1016/j.cell.2018.12.035), this may suggest that wild-type TDP-43 modeled by the authors create biomolecular condensates that are less liquidous than most condensates in the cell, including endogenous TDP-43. In this context, the manuscript would benefit from assays showing phase separation behavior and stability of the protein variants over time, such as live cell imaging and FRAP.

(8) Figure 1B: add phospho-specific staining since western blot uses antibodies against all forms of TDP-43, not specifically phospho-TPD-43, while it is interpreted as phosphorylated TDP-43 based on the higher molecular weight and the experimental conditions.

Minor comments (6 items):
(1) Lines should better be numbered.
(2) The term "Velcro balls" is not scientific, not well defined and misleading, in the sense that Velcro has two complementary components (velours ('velvet') & croché ('hook'), whereas TDP-43 overexpression is homotypic.
(3) Introduction should provide a brief explanation of amino acid substitution phospho- mimetic properties and limitations.
(4) The dexamethasone-induced nuclear translocation system should be introduced and cited (from another Dormann's paper).
(5) "coarse-grained and atomistic simulations" is not sufficiently explained.

(6) Cite and **discuss** Gasset-Rosa et al., 2019, (10.1016/j.neuron.2019.02.038) which demonstrated recruitment of TDP-43 phosphorylated at serines 409 and 410 into cytoplasmic TPD-43 droplets, that converted to a gel/solid-like state over time.

Referee #2:

This is a very nice study in which the authors complete a systematic and rigorous investigation into the impact of phosphorylation on the C-terminal tail of TDP-43. Although substantial changes are observed in vitro the readout, the authors do not observe a major impact on cellular function. Far from a 'null result', this is a critical and detailed investigation that should be used as a key example that observations in vitro need not report on relevant phenomenon in vivo. For example, I would have put good money on phosphorylation altering nuclear:cytosolic partitioning or RNA binding as assessed by EMSA - the fact that the 12D is equivalent to WT is, in my mind, a much more surprising result. I have a few minor comments but, frankly, the paper could probably be published as is, this is a tremendous amount of work and while there are of course always additional things that could be done, I do not consider additional experiments necessary.

In separate studies, Crabtree et al. and Bremer et al. recently showed that the net charge of IDR-containing RNA binding proteins tunes their driving force for assembly [1,2]. The authors should (1) calculate how the net charge on the protein is altered as a function of phosphorylation (2) consider if this aligns with the results observed by Bremer and Crabtree.

Schmidt et al reported that the distribution of hydrophobic/aromatic residues in TDP-43 appeared critical for its self-assembly [3]. The authors should consider how phosphosite placement may influence this, and if/how the residues examined by Schmidt interact as assessed by the molecular dynamics simulations. There are also parallels with the Schmidt et al work as observed here (i.e. there, loss of phase separation also did not influence splicing function). The authors may wish to comment/consider these parallels.

Some comment or explanation for the buffer dependence of reported HEPES dependence of assembly in the 12D constructed would be good (although the authors state no visible condensates for the 12D construction EV4, yet, there appear to be condensates?).

How might phosphorylation relate to the various structural modes of TDP-43 assembly proposed (RACS, helix-interaction, transient aromatic/hydrophobic contacts) - which of these does phosphorylation modulate?

1. Crabtree MD, Holland J, Kompella P, Babl L, Turner N, Baldwin AJ, et al. Repulsive electrostatic interactions modulate dense and dilute phase properties of biomolecular condensates. Cold Spring Harbor Laboratory. 2020. p. 2020.10.29.357863. doi:10.1101/2020.10.29.357863

2. Bremer A, Farag M, Borcherds WM, Peran I, Martin EW, Pappu RV, et al. Deciphering how naturally occurring sequence features impact the phase behaviors of disordered prion-like domains. Cold Spring Harbor Laboratory. 2021. p. 2021.01.01.425046. doi:10.1101/2021.01.01.425046

3. Schmidt HB, Barreau A, Rohatgi R. Phase separation-deficient TDP43 remains functional in splicing. Nat Commun. 2019;10: 4890.

Referee #3:

In this manuscript, da Silva et al. propose a protective role of the phosphorylation in the C-terminal LCD of TDP-43 against aggregate formation. TDP-43 is a major component and hyperphosphorylated in the aggregates of ALS and FTD neurons. It has been suggested that the hyperphosphorylation is involved in aggregate formation and disease progression. Here, authors studied the role of hyperphosphorylation in liquid-liquid phase separation (LLPS) of TDP-43 in vitro and in cultured cells, and found that the phosphorylation by CK1d or phosphorylation mimicking mutations suppressed the condensation of TDP-43 by increasing liquid-like behavior. Mutations also decreased the association of TDP-43 with stress granules (SG) and nuclear bodies in cultured cells. They indicate higher solvation of phospho-mimic amino acids by simulation. While these are all in vitro and cellular experiments, the results suggest a novel idea, which would lead a new direction of the research. Although the proposal should be tested in the future, the manuscript thus includes potential to be reported. Specific comments are described below.

Major comments:
TDP-43 aggregates are found in the cytoplasm of neurons. There is only one experiment using neurons in Fig. 6D, which shows the diffuse distribution of TDP-43 12D. The association of TDP-43 12D with SGs should be examined in neurons.

Need negative controls without expression of any TDP-43 variants in Fig. 5D, F, H, and Fig. EV8A.

In general, legends of figures are not described properly. The legends should explain things shown there but not the results. For example, in Fig. EV3 authors do not mention what is background, what is PrD like, what is NAP Vit and what 1 or 0 on the vertical scale. Moreover, it is known if the graphs are full length TDP-43, and where is LCD. If I read correctly the graphs, the right side of PrD like of WT changes the background level by D mutations. If so, why only the C-terminal region but not the whole LCD is converted?
Legends of Fig. 3, EV6 should also be written kindly to readers not familiar to those simulations.

Minor comments:
1. Fig. 1C, Fig. 6B, and Fig. EV4C. S/C or S/I ratio should be represented by the S/(S+C) ratio.
2. Fig. 1C. Which of cleaved TDP-43 or TDP-43-MBP-His6? Indicate in the legend. Why is TDP-43-MBP found in the fraction of the condensate? Is TDP-43-MBP pelleted with aggregated TDP-43?
3. What is a liquid like behavior (page 6, line 12)? Does it represent the dynamic behavior. Why does TDP-43 12D still retain condensates but not dissolve into solution if it has a liquid-like property?
4. I wonder if large condensates of 12D sediment so quickly that they can escape from the turbidity assay. The size of those condensates is not shown in Fig. 1H.
5. Fig. 2D and E. TDP-43 wt and 5D form small sizes of aggregates at 24 hours under a confocal microscopy, at that time they form only oligomers in SDD-AGE assay. Do all TDP-43 variants form large aggregates at 10 days when they show aggregates in SDD-AGE?
6. The nuclear localization assay is not described clearly. What is GCR? How does hormone treatment induce the nuclear localization?
7. Fig. 4C. CTF of endogenous TDP-43 can be faintly seen at around 30 kDa. Why CTF of Myc-TDP-43 move below the endogenous CTF whereas full length TDP-43 appear above endogenous TDP-43? Is it possible that 12D affect the cleaving site?
8. What is bkgr in Fig. EV9B?
9. Discussion. Authors do not explain clearly why hyperphosphorylated TDP-43 is enriched in its aggregates, even if hyperphosphorylated TDP-43 is proteolyzed quickly in normal condition.

Point-by point response to reviewers' comments:

Referee #1:

"Disease-linked TDP-43 hyperphosphorylation suppresses TDP-43 condensation and aggregation", by da Silva et al. 2021, EMBOJ

The RNA-binding protein TDP-43, is the major type of aggregated protein in ALS and FTD, two neurodegenerative disorders. Several C -terminal serine residues of TDP-43 are hyperphosphorylated in disease. In this work, da Silva and colleagues show that phosphorylation of TDP-43 mitigates its aggregation using in vitro and in silico approaches. Da Silva demonstrate that hyperphosphorylation reduces TDP-43 propensity to phase separate and aggregate. These findings are interesting, in part, because TDP-43 phosphorylation was initially assumed to promote, not inhibit, pathological aggregation. Authors suggest that TDP-43 hyperphosphorylation counteract aggregation.

Major concerns

In vitro analysis and simulations (5 items).
(1) This reviewer is not an expert in analysis and simulations.
(2) Quantify rigorously the multiscale simulations of TDP-43 LCD, and the Differences in subcellular behavior of Wt versus 12D TDP-43 (Figure 6D), which is not supported by any quantification.
To address this comment, we turned to an even more coarse-grained simulation method, the well-established hydrophobicity scale model (HPS) of Mittal et al. (Digon et al. PLoS Comput Biol, 2018) to rigorously quantify the differences in the phase behavior of Wt versus 12D TDP-43. By virtue of simplifying the description of the proteins even further, this approach enabled us to compute thermodynamic quantities from well-converged simulations. We have now added this quantification to the manuscript and now say on p. 8, line 24ff: "*Indeed, computing the excess free energy of transfer ΔG_{trans} from the density profile (Appendix Fig. S5D), which reports how favorable it is to move one chain from dilute solution at the saturation density to the dense phase of the condensate, showed that 12D LCDs are less prone to interact with each other in a condensate than Wt LCDs (Appendix Table S1).*"

(3) Do simulations predict that 12D proteins are less aggregation-prone, as was experimentally demonstrated in Figure 2. discuss how well does the model reflect the actual protein behavior?
Indeed, coarse-grained simulations with the HPS model predict that 12D mutant proteins are less prone to form condensates, as discussed above under point 2. We write "*In line with experiments on full-length TDP-43 (Fig. 2), 12D LCD phase-separated (Fig 3F,G), but more protein remained in the dilute phase compared to Wt.*" It is of course a simplification to compare simulations of LCDs with experiments on the full-length protein, but we consider it a good starting point, as we capture key experimental trends in our simulations. Our simulations with TDP-43 LCDs also capture that the 12A variant is considerably more prone to phase separation than Wt in experiments on full-length TDP-43. Importantly, phosphorylated LCD (with 12 serine residues phosphorylated, 12pS) phase-separates much less than the unmodified Wt LCD (new Fig. 3F-H), which is line with our experiments on phosphorylated full-length TDP-43 (Fig. 1 B,C).

(4) In simulations (Figure 3) test actual phosphorylated TDP-43 in parallel to mimetics.

We followed the reviewer's suggestion to elucidate this important point and have analyzed phosphorylated TDP-43 (12pS) in parallel to phosphomimetics in coarse-grained simulations with the HPS model (new Fig. 3F-H). We find that "… *(1) phosphorylation may indeed dissolve condensates and (2) that phosphorylation may have an even stronger effect than phosphomicking substitutions due to the larger negative charge of phospho-serine compared to aspartate"* (p. 8, last paragraph).

Cell biology (8 items)

(1) Definite experiments may resolve literature conflicts at the heart of the manuscript: There is a wealth of literature about the impact of TDP-43 phosphorylation on its biophysical properties with implications to disease. As authors correctly state, even the idea that phosphorylated TDP-43 reduce aggregation propensity and toxicity was tested in cell lines and Drosophila (Brady, Meng et al., 2011, Li, Yeh et al., 2011). Therefore, additional assessment of this well-studied topic should not be based on additional far approximations. The resolution to the conflicts in literature must depend on more definitive experimental means. Here, a primary concern that the authors should address in revisions is related to in-cell modeling of TDP-43. Over expression data should be substantiated by endogenous knock-in of the modified residues into the endogenous TARDBP gene.
We agree that it is important to substantiate our cellular overexpression data with additional data. Obtaining a cell line with endogenous knock-in of the modified residues was not possible within the revision time frame, however we have generated stable inducible FlpIn U2OS cell lines, in which myc-TDP-43 WT / 12D or 12A expression can be induced/titrated by doxycycline addition. These lines have a more homogenous expression pattern compared to transiently transfected cells, avoid high overexpression and show equal expression levels of the different TDP-43 variants (new figure EV4C, D). We replicated some of our results in these stable lines, i.e. we show that phosphomimetic TDP-43 12D does not have a deficiency in *TARDBP* autoregulation (new Fig. EV4D, E) and fails to condense into nuclear bodies (constitutively present in U2OS cells or induced by arsenite stress) but has a completely diffuse localization pattern, in contrast to TDP-43 WT and 12A (new Fig. EV4C and EV5C, D).
In addition, we would like to emphasize that in addition to our cellular experiments, where we obtained consistent results in three different cell types (HeLa cells, U2OS cells and rat primary neurons), we used completely different, complementary experimental approaches (*in vitro* experiments with purified TDP-43 and *in silico* experiments / simulations), which are in agreement with the cellular data and clearly demonstrate that phosphorylated/phosphomimetic TDP-43 is more soluble and less prone to phase separate/aggregate. Such complementary *in vitro/in silico* experiments have not been used in any previous studies. Thus, our conclusions are not based on "far approximations", but on consistent results in a variety of different experimental model systems.

(2) Shortcoming of modeling phosphorylation through "phosphomimetic" residues: Significant shortcoming in modeling phosphorylation through "phosphomimetic" residue substitution should be acknowledged in the case that the phosphorylation sites serve particular mechanisms of protein- protein interactions. Some phosphomimetic mutants are unable to recapitulate the binding to adapter proteins (e.g., Durocher et al., Mol Cell 4, Yaffe et al., Cell 91, 961-971 387-394). This should be briefly mentioned, although most in the field do not believe that disordered domains enable precise "binding pocket" type of interactions. More relevant to is the fact that multisite phosphorylation (=large number of phosphorylation sites) can produce critical changes to protein function through bulk electrostatics. This limitations should be acknowledged in the **discussion**.
This reviewer suggests the authors to consider if they wish to **discuss** the derived

hypothesis that the number of phosphorylation sites, but not the exact position of the negative charges is critical for changed activity. Furthermore, an experimental under-appreciation of true charge results from introduction of aspartate substitutions (−1) that does not match the charge introduced by Ser (−2). See also (Strickfaden 10.1016/j.cell.2006.12.032; Pearlman 10.1016/j.cell.2011.08.052; McLaughlin & Aderem 10.1016/s0968-0004(00)89042-8).
We agree that it is important to acknowledge the potential shortcomings of phosphomimetic mutations and therefore have included a sentence on this issue in the results (p. 3, line 23ff) and discussion (page 11, line 10ff). We also discuss the possibility that multisite phosphorylation may regulate TDP-43 through bulk electrostatics and cite some of the suggested literature (page 11, line 17ff).
We thank the reviewer for pointing out that phosphomimetic mutations are an experimental under-appreciation of true charge (aspartate = -1; phosphorylation = -2) and now mention this point in the results (p. 5, line 25ff) and discussion (page 11, line 13ff). This is supported by our new simulations data, where we directly compared TDP-43 LCD wild-type (WT), 12 phosphomimetic S-to-D mutations vs. 5 or 12 serines phosphorylated (5pS or 12pS) (new Fig. 3F-H), which revealed that 12pS has an even lower tendency to phase separate than the 12D variant, supporting the idea that phosphomimetic aspartate mutations under-estimate the effect of phospho groups.

(3) Phosphorylation protective outcome:
The 'protective' nature of TDP-43 phosphorylation in cells, is not appropriately supported by data. Particularly, there is a leap of faith in connecting biophysical properties of overexpressed protein to patho-physiology. Therefore, the authors should play down the interpretation that phosphorylation is a protective cellular mechanism that counteracts aberrant TDP-43 deleterious outcome, unless they explicitly describe the 'protected' property (by showing what are the downstream implications. For example obvious hypotheses may include differential clearance of different forms of TDP-43 or even further downstream differential death of cells).
We acknowledge that the “protective nature” of C-terminal TDP-43 phosphorylation at this point remains a hypothesis/speculation, based on our finding that TDP-43 phosphorylation disrupts condensation / aggregation, but does not interfere with basic RNA processing functions or nuclear localization of TDP-43. Our efforts to gain further insights into downstream implications, e.g. clearance rates or cytotoxicity, did not yield conclusive results and would require more in-depth evaluation that would go beyond the scope of this manuscript. Therefore, we followed the reviewer’s advice and toned down our interpretation that “TDP-43 phosphorylation could be a protective rather than a pathogenic modification” (i.e. we have this sentence from the discussion, or by making clear that this is a speculation (e.g. we say “we speculate…”, in the abstract, end of introduction or discussion).

(4) protein-protein interactions:
Substantiate experimental evidence in-cells that phosphorylated TDP-43 is less prone to engage in protein interactions, which is currently based primarily on simulations of mimetics. For example, authors can perform TDP-43 pulldown followed by proteomics, comparing unphosphorylated / phosphorylated TDP-43.
We agree that it will be highly interesting to investigate whether phosphorylation of TDP-43 alters additional protein-protein interactions (apart from TDP-43 self-self interaction). However, a full proteomic analysis including validation and functional follow-up would go beyond the scope of this current manuscript, hence we will perform a full proteomics analysis as part of a follow-up study. We added a sentence to the discussion acknowledging that *“Further research into the functional consequences of C-terminal TDP-43 phosphorylation, e.g. how it affects global protein or RNA interactions, TDP-43 stability or the introduction of*

additional PTMs, is needed to understand the role of TDP-43 phosphorylation in physiology and pathology" (page 12, line 15ff).

(5) Phosphorylation impact on TDP-43 cellular localization:
Reduce overstatement when concluding that hyperphosphorylation of TDP-43 does not impair subcellular localization or RNA regulation, as this is a negative result, tested in 'simple' HeLa cells. This is underscored by several reports that observed endogenous cytoplasmic TDP-43 granules in neurons (Alami et al., 2014 10.1016/j.neuron.2013.12.018; Gopal et al., 2017 10.1073/pnas.1614462114; Wang et al., 2008 10.1111/j.1471-4159.2007.05190.x).
To address the reviewer's concern, we have re-investigated the steady state localization of GFP-tagged TDP-43 Wt, 12 D and 12A in primary rat neurons and did not observe any obvious alteration in sublocalization, i.e. all variants were nuclear (new Appendix Fig. S8). Moreover, another cell type (U2OS cells with inducible expression of TDP-43 WT, 12D and 12A) also shows no defects in nuclear localization or TDP-43 autoregulation (new Fig. EV4C-E). Hence, the observed negative effects are not limited to HeLa cells.
However, to acknowledge the limitations of our study, we now point out that *"an effect on other RNA targets / RNA processing events or intracellular transport in other cell types cannot be excluded"* (p. 9, line 34ff). In the discussion we point out that *"Further research into the functional consequences of C-terminal TDP-43 phosphorylation, e.g. how it affects global protein or RNA interactions, TDP-43 stability or the introduction of additional PTMs, is needed to understand the role of TDP-43 phosphorylation in physiology and pathology"* (p.12, line 15ff).

(6) Phosphorylation impact of RNA biology:
TDP-43 targets hundreds of mRNAs and regulates their processing in different ways. Particularly, phosphorylation interferes with splicing function (Li et al., 2017 10.1074/jbc.M116.753913). Thus, it is advised that the preliminary analysis of RNA regulation in the original submission should be either taken out of the revised manuscript. An alternative is to perform an unbiased transcriptomic analysis that meets current standards in the field in neurons.
Indeed, the impact of TDP-43 phosphorylation on RNA processing is an interesting topic that warrants further in-depth investigation. The mentioned paper (Li et al., 2017) has studied MEK-induced phosphorylation of threonine 153 and tyrosine 155 (p-T153/Y155) and found that it alters TDP-43-regulated splicing and GU-rich RNA-binding, however the effect of C-terminal hyperphosphorylation on RNA-binding/regulation has not been examined so far. We agree that our analysis of RNA regulation should be extended by a full transcriptomic analysis / CLIP studies, however this analysis incl. validation would be beyond the scope of this manuscript.
We now acknowledge the limitations of our study and say that *"an effect on other RNA targets / RNA processing events or intracellular transport in other cell types cannot be excluded"* (p. 9, line 34ff), and in the discussion we added that *"Further research into the functional consequences of C-terminal TDP-43 phosphorylation, e.g. how it affects global protein or RNA interactions, TDP-43 stability or the introduction of additional PTMs, is needed to understand the role of TDP-43 phosphorylation in physiology and pathology"* (p. 12, line 15ff).

(7) Concern about TDP-43 liquidity in the overexpression system:
Based on manuscript-associated movies, the condensates remain defined entities and do not fuse to form a single mass with united center of gravity, as biomolecular condensates often do. Based on Ghosh and Zhou (10.1101/2020.06.22.164897) and Alberti (10.1016/j.cell.2018.12.035), this may suggest that wild-type TDP-43 modeled by the authors create biomolecular condensates that are less liquidous than most condensates in the cell,

including endogenous TDP-43. In this context, the manuscript would benefit from assays showing phase separation behavior and stability of the protein variants over time, such as live cell imaging and FRAP

The manuscript-associated movies were not collected from cells overexpressing TDP-43, but from purified TDP-43 protein (WT vs. 5D vs. 12D) in Hepes buffer, imaged by spinning disc confocal microscopy (described in the results on p.6, last paragraph). Indeed the movies show that TDP-43 WT does not form liquid-like condensates, whereas at least some slow fusion events are observed for 5D and 12D. Indeed, it seems likely that TDP-43 *in vitro* (without additional cellular factors, e.g. RNA) is more solid-like than TDP-43 condensates in cells, however it was not the purpose of our study to compare the *in vitro* and *in cell* behavior of TDP-43, but to compare the phase separation behavior of TDP-43 WT with phosphomimetic TDP-43.

We cannot compare the dynamics of the different protein variants in cells by live cell imaging or FRAP, as TDP-43 12D remains dispersed and fails to condense into membrane-less organelles (see Fig. 5 and 6) – so unfortunately this experiment could not be done.

(8) Figure 1B: add phospho-specific staining since western blot uses antibodies against all forms of TDP-43, not specifically phospho-TPD-43, while it is interpreted as phosphorylated TDP-43 based on the higher molecular weight and the experimental conditions.

Western blots with two different phospho-specific antibodies are shown in the expanded view figure EV1A (anti-pS409/410 and anti-pS403/404). Both phospho-specific antibodies selectively recognize the higher molecular weight band in the +CK1δ + ATP condition.

Minor comments (6 items):

(1) Lines should better be numbered.

We have now introduced line numbers in the main manuscript body.

(2) The term "Velcro balls" is not scientific, not well defined and misleading, in the sense that Velcro has two complementary components (velours ('velvet') & croché ('hook'), whereas TDP-43 overexpression is homotypic.

We have now rephrased the sentence and say *“condensates stuck to each other in a chain-like arrangement”* (p. 6, line 40).

(3) Introduction should provide a brief explanation of amino acid substitution phospho-mimetic properties and limitations.

We have added a brief explanation and the limitations to the results when we first talk about the phosphomimetic substitutions (p. 5, line 23ff) and also mention this topic again in the discussion (p. 11, line 10ff).

(4) The dexamethasone-induced nuclear translocation system should be introduced and cited (from another Dormann's paper).

In addition to citing our published study that utilized the dexamethasone-induce nuclear transport system (Hutten Usluer et al., Cell Reports 2020), we now briefly explain how the system works (p. 9, line 9ff): *“In this assay, a protein-of-interest harboring a nuclear localization signal (NLS) is fused to a tandem EGFP and two hormone binding domains of the glucocorticoid receptor (GCR), which retains the reporter protein in the cytoplasm. Upon addition of a steroid hormone (dexamethasone), the reporter protein is released from the cytoplasm and imported into the nucleus, by virtue of the NLS in the protein-of-interest. We examined reporters containing the different TDP-43 variants (Wt, 12D, 12A) and found that their import rates were indistinguishable (Fig. 4A, B).”*

(5) "coarse-grained and atomistic simulations" is not sufficiently explained.
We now preface our simulation results with a short explanation of atomistic and coarse-grained simulations (p. 7, line 34ff). "*In coarse-grained simulations we can access the relevant long time and large length scales to characterize phase behavior, while in atomistic simulations we can resolve the interactions of condensates with high resolution and high accuracy (Benayad et al., J. Chem Theory Comput, 2021, Dignon et al, PLoS Comput. Biol, 2018, Pietrek et al., J. Chem Theory Comput, 2020).*"

(6) Cite and **discuss** Gasset-Rosa et al., 2019, (10.1016/j.neuron.2019.02.038) which demonstrated recruitment of TDP-43 phosphorylated at serines 409 and 410 into cytoplasmic TPD-43 droplets, that converted to a gel/solid-like state over time.
Thanks for pointing out this finding, which we believe is in line with the idea that TDP-43 phosphorylation follows TDP-43 insolubility. The study reports that seeding of neuronal SHSY5Y cells with amyloid-like fibrils leads to the conversion of liquid like-TDP-43 droplets into phosphorylated cytoplasmic TDP-43 aggregates (phospho-409/410-positive). When exactly phosphorylation arises in this cell model has not been elucidated, but phosphorylation is detected at a late stage (30 days after fibril addition, earlier time points are not shown).
We have added a sentence on these findings by Gasset-Rosa et al. to our discussion (p. 12, line 11ff): "*In line with these findings, treatment of neuron-like cells with amyloid-like particles triggers a solidification of initially liquid-like cytoplasmic TDP-43 droplets, along with C-terminal TDP-43 phosphorylation (Gasset-Rosa et al., Neuron 2019)".*

Referee #2:

This is a very nice study in which the authors complete a systematic and rigorous investigation into the impact of phosphorylation on the C-terminal tail of TDP-43. Although substantial changes are observed in vitro the readout, the authors do not observe a major impact on cellular function. Far from a 'null result', this is a critical and detailed investigation that should be used as a key example that observations in vitro need not report on relevant phenomenon in vivo. For example, I would have put good money on phosphorylation altering nuclear:cytosolic partitioning or RNA binding as assessed by EMSA - the fact that the 12D is equivalent to WT is, in my mind, a much more surprising result. I have a few minor comments but, frankly, the paper could probably be published as is, this is a tremendous amount of work and while there are of course always additional things that could be done, I do not consider additional experiments necessary.
We thank the reviewer for the positive comments and appreciation of our work and results.

In separate studies, Crabtree et al. and Bremer et al. recently showed that the net charge of IDR-containing RNA binding proteins tunes their driving force for assembly [1,2]. The authors should (1) calculate how the net charge on the protein is altered as a function of phosphorylation (2) consider if this aligns with the results observed by Bremer and Crabtree.
Thanks for pointing out these interesting manuscripts! As suggested, we have calculated the net charge of TDP-43 at physiological pH and how it's altered by phosphomimetic mutations / phosphorylation and discuss how this aligns with the results by Bremer and Crabtree (p. 11, line 21ff): *"Indeed, two recent studies showed that the net charge of IDR-containing RBPs tunes their driving force for assembly (Crabtree et al, 2021; Bremer et al., 2021). For instance, reducing the net charge of the disordered region of Ddx4 promotes its phase separation (Crabtree 2021), and increasing the net charge of the low complexity region of hnRNP-A1 reduces its phase separation, likely due to repulsive electrostatic long-range interactions (Bremer et al., 2021). At physiological pH, TDP-43 has a net charge of -4.1,*

phosphomimetic 12D TDP-43 has a net charge of -16.1 and 12x phosphorylated TDP-43 has a net charge of -28.1. In line with the aforementioned studies, it seems likely that the strong increase in negative net charge in phosphomimitic/phosphorylated TDP-43 is responsible for the reduced propensity to self-assemble into condensates."

Schmidt et al reported that the distribution of hydrophobic/aromatic residues in TDP-43 appeared critical for its self-assembly [3]. The authors should consider how phosphosite placement may influence this, and if/how the residues examined by Schmidt interact as assessed by the molecular dynamics simulations. There are also parallels with the Schmidt et al work as observed here (i.e. there, loss of phase separation also did not influence splicing function). The authors may wish to comment/consider these parallels.
Thanks for pointing out this study, which we now cite when we mention the importance of aromatic residues in driving phase separation (p. 8, line 5 and discussion p.11, line 34). From our simulations, it appears that the interactions between aromatic residues are not strongly affected by C-terminal serine phosphorylation, as shown in the contact maps (Fig. EV3). We now point this out in the discussion (p. 11, line 37ff): "… *the contact maps extracted from the simulations of the TDP-43 LCD show that most interactions, including aromatic interactions, are not strongly affected by C-terminal phosphomimetics and that mainly interactions of serines with nearby residues are reduced. However more work is required to understand how post-translational modifications modulate aromatic "sticker"-"sticker" interactions on the molecular scale."*
We now also comment on the similarities to the Schmidt et al., 2019 finding that phase separation-deficient TDP-43 remains functional in splicing (p. 13, line 1f), which is in line with our findings for the 12D mutant. ("*In line with our findings, an earlier study found that phase separation-deficient TDP-43 remains competent in splicing regulation (Schmidt et al., Nat Comm 2019)".*

Some comment or explanation for the buffer dependence of reported HEPES dependence of assembly in the 12D constructed would be good (although the authors state no visible condensates for the 12D construction EV4, yet, there appear to be condensates?
We have rephrased the results text to former figure EV4 (now EV2) and now state more correctly that "*12D formed only very few, small condensates in phosphate buffer" (*p. 6).
We also try to give an explanation for the buffer dependence and speculate why only very few 12D condensates form in phosphate buffer (p. 6, line 29) *"…possibly because the ions in phosphate buffer may screen certain attractive interactions between TDP-43 molecules and disfavor phase separation*."

How might phosphorylation relate to the various structural modes of TDP-43 assembly proposed (RACS, helix-interaction, transient aromatic/hydrophobic contacts) - which of these does phosphorylation modulate?
Indeed, different structural modes and interactions have been proposed for TDP-43 assembly, and we agree that it is interesting to speculate on how phosphorylation might affect these different modes. We had already commented on a recent cryo-EM structure of the TDP-43-LCD by Li, Babinchak et al., 2021, showing that several C-terminal serines are buried inside the fibril structure, hence we suggested that phosphorylation might disrupt this amyloid fibril structure (p. 11, line 41ff). We have now expanded this paragraph and also comment on other interaction modes (helix interactions, aromatic/hydrophobic interactions) (p. 11, line 31ff): *"Various modes of TDP-43 assembly have been proposed, including homotypic interactions of an a-helical structure in the conserved region (CR) of the LCD (Conicella 2016, Conicella 2020) and interactions between aromatic/aliphatic residues in the LCD (Li et al., 2018; Schmidt et al., 2020; Laurents et al., 2021). All phosphomimetic mutations examined in our study are outside of the a-helix / CR (aa. 321 – 360) (Fig. 1D),*

hence they are unlikely to interfere with helix-helix interactions. In line with this hypothesis, the contact maps extracted from the simulations of the TDP-43 LCD show that most interactions, including aromatic interactions, are not strongly affected by C-terminal phosphorylation and that mainly interactions of serines with nearby residues are reduced. However, more work is required to understand how post-translational modifications modulate aromatic "sticker"-"sticker" interactions on the molecular scale. C-terminal phosphorylation may affect amyloid-like fibril formation: A recent cryo-EM structure of solid TDP-43 LCD fibrils showed that several C-terminal serines are buried inside the fibril structure (Li, Babinchak et al., 2021), hence phosphorylation could disrupt the amyloid fibril structure, in line with our experimental findings that TDP-43 aggregate formation is strongly reduced."

1. Crabtree MD, Holland J, Kompella P, Babl L, Turner N, Baldwin AJ, et al. Repulsive electrostatic interactions modulate dense and dilute phase properties of biomolecular condensates. Cold Spring Harbor Laboratory. 2020. p. 2020.10.29.357863. doi:10.1101/2020.10.29.357863
2. Bremer A, Farag M, Borcherds WM, Peran I, Martin EW, Pappu RV, et al. Deciphering how naturally occurring sequence features impact the phase behaviors of disordered prion-like domains. Cold Spring Harbor Laboratory. 2021. p. 2021.01.01.425046. doi:10.1101/2021.01.01.425046
3. Schmidt HB, Barreau A, Rohatgi R. Phase separation-deficient TDP43 remains functional in splicing. Nat Commun. 2019;10: 4890.

Referee #3:

In this manuscript, da Silva et al. propose a protective role of the phosphorylation in the C-terminal LCD of TDP-43 against aggregate formation. TDP-43 is a major component and hyperphosphorylated in the aggregates of ALS and FTD neurons. It has been suggested that the hyperphosphorylation is involved in aggregate formation and disease progression. Here, authors studied the role of hyperphosphorylation in liquid-liquid phase separation (LLPS) of TDP-43 in vitro and in cultured cells, and found that the phosphorylation by CK1d or phosphorylation mimicking mutations suppressed the condensation of TDP-43 by increasing liquid-like behavior. Mutations also decreased the association of TDP-43 with stress granules (SG) and nuclear bodies in cultured cells. They indicate higher solvation of phospho-mimic amino acids by simulation. While these are all in vitro and cellular experiments, the results suggest a novel idea, which would lead a new direction of the research. Although the proposal should be tested in the future, the manuscript thus includes potential to be reported. Specific comments are described below.

Major comments:
TDP-43 aggregates are found in the cytoplasm of neurons. There is only one experiment using neurons in Fig. 6D, which shows the diffuse distribution of TDP-43 12D. The association of TDP-43 12D with SGs should be examined in neurons.
As suggested, we have now examined the association of cytosolically mislocalized TDP-43 12D vs. 12A and WT with SGs in primary rat neurons. This experiment is shown in new Fig. 6E, it shows that also in rat neurons the phosphomimetic 12D mutant is hardly recruited into heat-shock induced SGs and remains dispersed in the cytoplasm, while TDP-43 WT and 12A are prominently recruited into SGs. This provides further evidence that phosphorylation prevents TDP-43 condensation / phase transitions in neurons.

Need negative controls without expression of any TDP-43 variants in Fig. 5D, F, H, and Fig. EV8A.
We have now included the negative controls to these corresponding figures in Appendix Fig. S6, i.e. we show staining of endogenous TDP-43 (in control and TDP-43 siRNA-treated cells), without expression of any TDP-43 variant.

In general, legends of figures are not described properly. The legends should explain things shown there but not the results. For example, in Fig. EV3 authors do not mention what is background, what is PrD like, what is NAP Vit and what 1 or 0 on the vertical scale. Moreover, it is known if the graphs are full length TDP-43, and where is LCD. If I read correctly the graphs, the right side of PrD like of WT changes the background level by D mutations. If so, why only the C-terminal region but not the whole LCD is converted? Legends of Fig. 3, EV6 should also be written kindly to readers not familiar to those simulations.
We apologize that our legends were not clear. We have made an effort to explain in more detail what is shown in each figure (we prefer to also state the major result for clarity). In particular in Fig. EV3 (now Appendix Fig. S2), we have included a scheme of TDP-43, to make clear that the model shows full-length TDP-43 and where the LCD is located, and we have explained better what is shown in the figure.
We also have rephrased the legend of Fig. 3 and corresponding extended/supplementary figures in an attempt to make the simulations more amenable to readers not familiar with this kind of data.

Minor comments:
1. Fig. 1C, Fig. 6B, and Fig. EV4C. S/C or S/I ratio should be represented by the S/(S+C) ratio.
We have made the requested change and now display the S/S+C (total) ration instead of S/C ratio in the mentioned figures (now Fig. 1C, Fig EV2C and Fig. 6B).

2. Fig. 1C. Which of cleaved TDP-43 or TDP-43-MBP-His6? Indicate in the legend. Why is TDP-43-MBP found in the fraction of the condensate? Is TDP-43-MBP pelleted with aggregated TDP-43?
The quantification shown in Fig. 1C is cleaved TDP-43 (now indicated in the legend). The reviewer is correct that the remaining uncleaved TDP-43-MBP tends to co-fractionate with TDP-43, due to self-self interactions of TDP-43 molecules.

3. What is a liquid like behavior (page 6, line 12)? Does it represent the dynamic behavior. Why does TDP-43 12D still retain condensates but not dissolve into solution if it has a liquid-like property?
The term “liquid-like” is commonly used to describe condensates formed by liquid-liquid phase separation that behave like a liquid, i.e. they fuse with one another and then relax back into a round sphere (like water droplets), see e.g. Patel et al., Cell 2015 for a classical paper that uses this term. Indeed it indicates a dynamic behavior of the molecules. In contrast, molecules in viscous or solid-like condensates show a less dynamic behavior.

TDP-43 12D, at least in Hepes buffer, still forms condensates by liquid liquid phase separation, i.e. it still can weakly self-interact to form dynamic, liquid-like condensates. In phosphate buffer, these self-interactions are more strongly reduced (possibly due to the charged buffer molecules weaking the interactions, see our comment on p. 6, line 29ff) – under these conditions, 12D hardly forms any condensates but remains largely dispersed in solution (shown in Fig. EV2D).

4. I wonder if large condensates of 12D sediment so quickly that they can escape from the turbidity assay. The size of those condensates is not shown in Fig. 1H.
Indeed, this is what we assume, hence the turbidity assay shown in Fig. 1E shows quite low absorption values for 12D (we already had made a remark about this on p.6, line 22 ff (“*Note that these few large condensates most likely escape detection in the turbidity assay due to rapid sedimentation during the assay”*).
The size of the condensates can be appreciated from the bright field microscopy images shown in Fig. 1F, a size quantification is shown in Fig. 1H.

5. Fig. 2D and E. TDP-43 wt and 5D form small sizes of aggregates at 24 hours under a confocal microscopy, at that time they form only oligomers in SDD-AGE assay. Do all TDP-43 variants form large aggregates at 10 days when they show aggregates in SDD-AGE?
As explained in the results and methods, the confocal microscopy assay and the SDD-AGE assay shown in Fig. 2D and E were carried out under slightly different conditions: For the microscopy assay, the MBP-tag was cleaved off by TEV, which leads rapid formation of small aggregates (within a few hours). When analyzed by SDD-AGE under the same assay conditions, a high molecular weight smear is seen immediately after TEV addition, but no distinct oligomeric species can be distinguished (see Appendix Fig. S3, right gel).
For the SDD-AGE assay shown in Fig. 2E, the MBP tag was not cleaved off, and under these assay conditions TDP-43-MBP forms distinct oligomers within 1-2 days and high molecular weight species only after 3 days (see Appendix Fig. S3, left gel and Fig. 2E). Therefore, the assays shown in Fig. 2D and E cannot be directly compared.

To clarify that the two assays were carried out under different assays conditions, we have added a scheme above the images/gels, illustrating how the assays were carried out (Fig. 2D/E).

6. The nuclear localization assay is not described clearly. What is GCR? How does hormone treatment induce the nuclear localization?
As outlined above under reviewer 1’s comments, we have now included some explanatory sentences (p. 9, line 9ff): *“In this assay, a protein-of-interest harboring a nuclear localization signal (NLS) is fused to a tandem EGFP and two hormone binding domains of the glucocorticoid receptor (GCR), which retains the reporter protein in the cytoplasm. Upon addition of a steroid hormone (dexamethasone), the reporter protein is released from the cytoplasm and imported into the nucleus, by virtue of the NLS in the protein-of-interest. We examined reporters containing the different TDP-43 variants (Wt, 12D, 12A) and found that their import rates were indistinguishable (Fig. 4A, B).”*

7. Fig. 4C. CTF of endogenous TDP-43 can be faintly seen at around 30 kDa. Why CTF of Myc-TDP-43 move below the endogenous CTF whereas full length TDP-43 appear above endogenous TDP-43? Is it possible that 12D affect the cleaving site?
The faint band around 30 kDa is most likely an unspecific band that comes up with the TDP-43 antibody, but not a CTF of endogenous TDP-43 (we have now added an asterisk to the band and labelled it as “unspecific band” in the legend to clarify this point). We usually do not see CTFs of endogenous TDP-43 in HeLa cells under normal conditions, our previous publication shows that 35 and 25 kDa CTFs only arise after caspase activation but not in untreated cells (Dormann et al., J Neurochem 2009).

8. What is bkgr in Fig. EV9B?
bkgr = background; we now define this abbreviation in the legend of this figure (now Fig. EV5B). It was obtained by omitting recombinant TDP-43-MBP, but otherwise maintaining equal processing and analysis conditions. Hence the obtained values correspond likely to

autofluorescence/photomultiplier tube noise/channel crosstalk, or bleed-through, or unspecific staining of the MBP antibody.

9. Discussion. Authors do not explain clearly why hyperphosphorylated TDP-43 is enriched in its aggregates, even if hyperphosphorylated TDP-43 is proteolyzed quickly in normal condition.
Indeed it remains an open question why hyperphosphorylated TDP-43 is enriched in aggregates in ALS/FTD patients, even though we clearly find that hyperphosphorylation antagonizes TDP-43 condensation / aggregation. In our original discussion, we meant to speculate that hyperphosphorylated TDP-43 may arise constitutively to keep TDP-43 soluble, but then is quickly proteolyzed and therefore evades detection under normal conditions, so only if protein degradation is impaired (e.g. in the ageing brain) it may accumulate and then aggregate due to elevated TDP-43 levels. We attempted to experimentally address this hypothesis during the revision period, but unfortunately did not obtain conclusive results. As our speculation seemed to raise confusion, we have removed this section from the discussion. Instead, we now point out the open questions that remain to be clarified in the future and discuss several studies that indicate that TDP-43 phosphorylation follows TDP-43 insolubility (p. 12, line 6ff):
"Under what conditions C-terminal TDP-43 phosphorylation arises in cells and which form of TDP-43 (soluble, phase separated, aggregated) is phosphorylated is still unknown. Interestingly, we and others previously found that C-terminal TDP-43 phosphorylation follows TDP-43 insolubility, suggesting that phosphorylation arises downstream of TDP-43 aggregation (Brady et al., 2011, Dormann, Capell et al., 2009, Zhang, Fan et al., 2019). In line with these findings, treatment of neuron-like cells with amyloid-like particles triggers a solidification of initially liquid-like cytoplasmic TDP-43 droplets, along with C-terminal TDP-43 phosphorylation (Gasset-Rosa Neuron 2019). Why phosphorylated TDP-43 aggregates nevertheless persist and are not readily disassembled after phosphorylation remains to be investigated. Further research into the functional consequences of C-terminal TDP-43 phosphorylation, e.g. how it affects global protein or RNA interactions, TDP-43 stability or the introduction of additional PTMs, is needed to understand the role of TDP-43 phosphorylation in physiology and pathology."

1st Revision - Editorial Decision 15th Dec 2021

Dear Dorothee,

Thanks for submitting your revised manuscript to The EMBO Journal. Your manuscript has now been seen by referee # 3. As you can see below, the referee appreciates the introduced changes and supports publication here.

I am therefore very pleased to accept the manuscript. Before sending you the formal acceptance letter there are just a few things to sort out.

The contributions of Lisa Pietrek is missing in the author contribution section.

The reference list: for articles with more than 10 authors the author list should be cut after 10 authors followed by et al.

Please double check that all the grants are listed in the MS.

Fig 1G, Fig EV3A+B and Appendix Fig 5A+B callouts are missing.

The Materials & Methods section should follow the Discussion section.

The movies need to be zipped with their legends. The legends should be removed from the manuscript file.

Our publisher has also done their pre-publication check on your manuscript. When you log into the manuscript submission system you will see the file "Data Edited Manuscript file". Please take a look at the word file and the comments regarding the figure legends and respond to the issues.

We encourage the publication of source data, particularly for electrophoretic gels and blots, with the aim of making primary data more accessible and transparent to the reader. It would be great if you could provide me with a PDF file per figure that contains the original, uncropped and unprocessed scans of all or key gels used in the figure? The PDF files should be labelled with the appropriate figure/panel number and should have molecular weight markers; further annotation could be useful but is not essential. The PDF files will be published online with the article as supplementary "Source Data" files.

That should be all. You can use the link below to upload the files. Let me know if you have any further questions.

Best Karin

Karin Dumstrei, PhD
Senior Editor
The EMBO Journal

Guide For Authors: https://www.embopress.org/page/journal/14602075/authorguide

We realize that it is difficult to revise to a specific deadline. In the interest of protecting the conceptual advance provided by the work, we recommend a revision within 3 months (15th Mar 2022). Please discuss the revision progress ahead of this time with the editor if you require more time to complete the revisions. Use the link below to submit your revision:

https://emboj.msubmit.net/cgi-bin/main.plex

Referee #3:

These authors have responded my previous comments properly. Therefore, I have no more comments.

2nd Revision - Editorial Decision 4th Jan 2022

Dear Dorothee,

Thank you for submitting your revised manuscript to The EMBO Journal. I have now had a chance to take a careful look at everything and all looks good!

I am therefore very pleased to accept the manuscript for publication here.

With best wishes

Karin

Karin Dumstrei, PhD
Senior Editor
The EMBO Journal

Please note that it is EMBO Journal policy for the transcript of the editorial process (containing referee reports and your response letter) to be published as an online supplement to each paper. If you do NOT want this, you will need to inform the Editorial Office via email immediately. More information is available here:
https://www.embopress.org/page/journal/14602075/authorguide#transparentprocess

Your manuscript will be processed for publication in the journal by EMBO Press. Manuscripts in the PDF and electronic editions of The EMBO Journal will be copy edited, and you will be provided with page proofs prior to publication. Please note that supplementary information is not included in the proofs.

Please note that you will be contacted by Wiley Author Services to complete licensing and payment information. The 'Page Charges Authorization Form' is available here: https://www.embopress.org/pb-assets/embo-site/tej_apc.pdf

Should you be planning a Press Release on your article, please get in contact with embojournal@wiley.com as early as possible, in order to coordinate publication and release dates.

If you have any questions, please do not hesitate to call or email the Editorial Office. Thank you for your contribution to The EMBO Journal.

Yours sincerely,

** Click here to be directed to your login page: https://emboj.msubmit.net

EMBO PRESS

YOU MUST COMPLETE ALL CELLS WITH A PINK BACKGROUND ↓

PLEASE NOTE THAT THIS CHECKLIST WILL BE PUBLISHED ALONGSIDE YOUR PAPER

Corresponding Author Name: Dorothee Dormann
Journal Submitted to: The EMBO Journal
Manuscript Number: EMBOJ-2021-108443R

Reporting Checklist For Life Sciences Articles (Rev. June 2017)

This checklist is used to ensure good reporting standards and to improve the reproducibility of published results. These guidelines are consistent with the Principles and Guidelines for Reporting Preclinical Research issued by the NIH in 2014. Please follow the journal's authorship guidelines in preparing your manuscript.

A- Figures

1. Data

The data shown in figures should satisfy the following conditions:

- the data were obtained and processed according to the field's best practice and are presented to reflect the results of the experiments in an accurate and unbiased manner.
- figure panels include only data points, measurements or observations that can be compared to each other in a scientifically meaningful way.
- graphs include clearly labeled error bars for independent experiments and sample sizes. Unless justified, error bars should not be shown for technical replicates.
- if n< 5, the individual data points from each experiment should be plotted and any statistical test employed should be justified
- Source Data should be included to report the data underlying graphs. Please follow the guidelines set out in the author ship guidelines on Data Presentation.

2. Captions

Each figure caption should contain the following information, for each panel where they are relevant:

- a specification of the experimental system investigated (eg cell line, species name).
- the assay(s) and method(s) used to carry out the reported observations and measurements
- an explicit mention of the biological and chemical entity(ies) that are being measured.
- an explicit mention of the biological and chemical entity(ies) that are altered/varied/perturbed in a controlled manner.

- the exact sample size (n) for each experimental group/condition, given as a number, not a range;
- a description of the sample collection allowing the reader to understand whether the samples represent technical or biological replicates (including how many animals, litters, cultures, etc.).
- a statement of how many times the experiment shown was independently replicated in the laboratory.
- definitions of statistical methods and measures:
  - common tests, such as t-test (please specify whether paired vs. unpaired), simple χ2 tests, Wilcoxon and Mann-Whitney tests, can be unambiguously identified by name only, but more complex techniques should be described in the methods section;
  - are tests one-sided or two-sided?
  - are there adjustments for multiple comparisons?
  - exact statistical test results, e.g., P values = x but not P values < x;
  - definition of 'center values' as median or average;
  - definition of error bars as s.d. or s.e.m.

Any descriptions too long for the figure legend should be included in the methods section and/or with the source data.

In the pink boxes below, please ensure that the answers to the following questions are reported in the manuscript itself. Every question should be answered. If the question is not relevant to your research, please write NA (non applicable). We encourage you to include a specific subsection in the methods section for statistics, reagents, animal models and human subjects.

USEFUL LINKS FOR COMPLETING THIS FORM

http://www.antibodypedia.com
http://1degreebio.org
http://www.equator-network.org/reporting-guidelines/improving-bioscience-research-repo
http://grants.nih.gov/grants/olaw/olaw.htm
http://www.mrc.ac.uk/Ourresearch/Ethicsresearchguidance/Useofanimals/index.htm
http://ClinicalTrials.gov
http://www.consort-statement.org
http://www.consort-statement.org/checklists/view/32-consort/66-title
http://www.equator-network.org/reporting-guidelines/reporting-recommendations-for-tun
http://datadryad.org
http://figshare.com
http://www.ncbi.nlm.nih.gov/gap
http://www.ebi.ac.uk/ega
http://biomodels.net/
http://biomodels.net/miriam/
http://jjj.biochem.sun.ac.za
https://osp.od.nih.gov/biosafety-biosecurity-and-emerging-biotechnology/
http://www.selectagents.gov/

B- Statistics and general methods

	Please fill out these boxes ↓ (Do not worry if you cannot see all your text once you press return)
1.a. How was the sample size chosen to ensure adequate power to detect a pre-specified effect size?	No estimation of statistical power was used to pre-determine sample size. Sample size was estimated based on previous experience with the assay.
1.b. For animal studies, include a statement about sample size estimate even if no statistical methods were used.	NA
2. Describe inclusion/exclusion criteria if samples or animals were excluded from the analysis. Were the criteria pre-established?	NA
3. Were any steps taken to minimize the effects of subjective bias when allocating animals/samples to treatment (e.g. randomization procedure)? If yes, please describe.	NA
For animal studies, include a statement about randomization even if no randomization was used.	NA
4.a. Were any steps taken to minimize the effects of subjective bias during group allocation or/and when assessing results (e.g. blinding of the investigator)? If yes please describe.	NA
4.b. For animal studies, include a statement about blinding even if no blinding was done	NA
5. For every figure, are statistical tests justified as appropriate?	Yes
Do the data meet the assumptions of the tests (e.g., normal distribution)? Describe any methods used to assess it.	Yes. Normal distribution was tested using the Shapiro-Wilk test and normal Q-Q plot.

Is there an estimate of variation within each group of data?	Yes
Is the variance similar between the groups that are being statistically compared?	Yes, using the Brown-Forsythe test.

C- Reagents

6. To show that antibodies were profiled for use in the system under study (assay and species), provide a citation, catalog number and/or clone number, supplementary information or reference to an antibody validation profile. e.g., Antibodypedia (see link list at top right), 1DegreeBio (see link list at top right).	The catalog number of each primary antibodies used for WB and IF are provided in the materials and methods part when mentioned.
7. Identify the source of cell lines and report if they were recently authenticated (e.g., by STR profiling) and tested for mycoplasma contamination.	NA

* for all hyperlinks, please see the table at the top right of the document

D- Animal Models

8. Report species, strain, gender, age of animals and genetic modification status where applicable. Please detail housing and husbandry conditions and the source of animals.	NA
9. For experiments involving live vertebrates, include a statement of compliance with ethical regulations and identify the committee(s) approving the experiments.	NA
10. We recommend consulting the ARRIVE guidelines (see link list at top right) (PLoS Biol. 8(6), e1000412, 2010) to ensure that other relevant aspects of animal studies are adequately reported. See author guidelines, under 'Reporting Guidelines'. See also: NIH (see link list at top right) and MRC (see link list at top right) recommendations. Please confirm compliance.	NA

E- Human Subjects

11. Identify the committee(s) approving the study protocol.	NA
12. Include a statement confirming that informed consent was obtained from all subjects and that the experiments conformed to the principles set out in the WMA Declaration of Helsinki and the Department of Health and Human Services Belmont Report.	NA
13. For publication of patient photos, include a statement confirming that consent to publish was obtained.	NA
14. Report any restrictions on the availability (and/or on the use) of human data or samples.	NA
15. Report the clinical trial registration number (at ClinicalTrials.gov or equivalent), where applicable.	NA
16. For phase II and III randomized controlled trials, please refer to the CONSORT flow diagram (see link list at top right) and submit the CONSORT checklist (see link list at top right) with your submission. See author guidelines, under 'Reporting Guidelines'. Please confirm you have submitted this list.	NA
17. For tumor marker prognostic studies, we recommend that you follow the REMARK reporting guidelines (see link list at top right). See author guidelines, under 'Reporting Guidelines'. Please confirm you have followed these guidelines.	NA

F- Data Accessibility

18: Provide a "Data Availability" section at the end of the Materials & Methods, listing the accession codes for data generated in this study and deposited in a public database (e.g. RNA-Seq data: Gene Expression Omnibus GSE39462, Proteomics data: PRIDE PXD000208 etc.) Please refer to our author guidelines for 'Data Deposition'.<br><br>Data deposition in a public repository is mandatory for:<br>a. Protein, DNA and RNA sequences<br>b. Macromolecular structures<br>c. Crystallographic data for small molecules<br>d. Functional genomics data<br>e. Proteomics and molecular interactions	NA
19. Deposition is strongly recommended for any datasets that are central and integral to the study; please consider the journal's data policy. If no structured public repository exists for a given data type, we encourage the provision of datasets in the manuscript as a Supplementary Document (see author guidelines under 'Expanded View' or in unstructured repositories such as Dryad (see link list at top right) or Figshare (see link list at top right).	
20. Access to human clinical and genomic datasets should be provided with as few restrictions as possible while respecting ethical obligations to the patients and relevant medical and legal issues. If practically possible and compatible with the individual consent agreement used in the study, such data should be deposited in one of the major public access-controlled repositories such as dbGAP (see link list at top right) or EGA (see link list at top right).	NA
21. Computational models that are central and integral to a study should be shared without restrictions and provided in a machine-readable form. The relevant accession numbers or links should be provided. When possible, standardized format (SBML, CellML) should be used instead of scripts (e.g. MATLAB). Authors are strongly encouraged to follow the MIRIAM guidelines (see link list at top right) and deposit their model in a public database such as Biomodels (see link list at top right) or JWS Online (see link list at top right). If computer source code is provided with the paper, it should be deposited in a public repository or included in supplementary information.	

G- Dual use research of concern

22. Could your study fall under dual use research restrictions? Please check biosecurity documents (see link list at top right) and list of select agents and toxins (APHIS/CDC) (see link list at top right). According to our biosecurity guidelines, provide a statement only if it could.	NA
